# *Plasmodium vivax* Sporozoite Challenge in Malaria-Naïve and Semi-Immune Colombian Volunteers

**DOI:** 10.1371/journal.pone.0099754

**Published:** 2014-06-25

**Authors:** Myriam Arévalo-Herrera, David A. Forero-Peña, Kelly Rubiano, José Gómez-Hincapie, Nora L. Martínez, Mary Lopez-Perez, Angélica Castellanos, Nora Céspedes, Ricardo Palacios, José Millán Oñate, Sócrates Herrera

**Affiliations:** 1 Malaria Vaccine and Drug Development Center (MVDC), Cali, Colombia; 2 School of Health, Universidad del Valle, Cali, Colombia; 3 Caucaseco Scientific Research Center (CSRC), Cali, Colombia; 4 Meridional R&D, São Paulo, Brazil; 5 Centro Médico Imbanaco, Cali, Colombia; University of California Los Angeles, United States of America

## Abstract

**Background:**

Significant progress has been recently achieved in the development of *Plasmodium vivax* challenge infections in humans, which are essential for vaccine and drug testing. With the goal of accelerating clinical development of malaria vaccines, the outcome of infections experimentally induced in naïve and semi-immune volunteers by infected mosquito bites was compared.

**Methods:**

Seven malaria-naïve and nine semi-immune Colombian adults (n = 16) were subjected to the bites of 2–4 *P. vivax* sporozoite-infected *Anopheles* mosquitoes. Parasitemia levels, malaria clinical manifestations, and immune responses were assessed and compared.

**Results:**

All volunteers developed infections as confirmed by microscopy and RT-qPCR. No significant difference in the pre-patent period (mean 12.5 and 12.8 days for malaria-naïve and malaria-exposed, respectively) was observed but naïve volunteers developed classical malaria signs and symptoms, while semi-immune volunteers displayed minor or no symptoms at the day of diagnosis. A malaria-naïve volunteer developed a transient low submicroscopic parasitemia that cured spontaneously. Infection induced an increase in specific antibody levels in both groups.

**Conclusion:**

Sporozoite infectious challenge was safe and reproducible in semi-immune and naïve volunteers. This model will provide information for simultaneous comparison of the protective efficacy of *P. vivax* vaccines in naïve and semi-immune volunteers under controlled conditions and would accelerate *P. vivax* vaccine development.

**Trial Registration:**

clinicaltrials.gov NCT01585077

## Introduction

Despite multiple technical and financial constraints for *Plasmodium vivax* malaria research, significant efforts have been invested and progress has been achieved towards development of an effective *P. vivax* vaccine. Two *P. vivax* parasite antigens, the circumsporozoite (CS) protein [1], [2] and the oocyst/ookinete Pvs25 protein [3], [4], have reached clinical development and have been tested in Phase I vaccine trials. Several others have been or are currently under preclinical testing [1], [5]-[8]. Furthermore, successful efforts are being made on the discovery of novel *P. vivax* antigens that could be proposed for vaccine development [9].

As with *P. falciparum*, the *P. vivax* CS protein is among the most promising vaccine candidates. *P. vivax* CS-derived subunit vaccine formulations based on Long Synthetic Peptides (LSP) formulated in Montanide adjuvant have been shown to be safe, well-tolerated and immunogenic in malaria-naïve volunteers [1], [10]–[12], and therefore have enabled progression to protective efficacy trials.

The protective efficacy of malaria vaccine candidates that have proven to be safe and immunogenic in Phase I trials, can be tested in malaria-naïve volunteers in Phase IIa trials [13], [14]. Such testing is usually performed in small numbers of volunteers who are vaccinated and then exposed to experimental parasite challenge with either infectious sporozoites [15]–[18] or asexual blood stages to assess the vaccine capacity to prevent infection or reduce its clinical manifestations [19].

Because of the constraints to grow *P. vivax* in culture [20], production of infected mosquitoes for sporozoite challenge trials should be carried out in malaria-endemic areas where parasites are readily accessible. Additionally, Phase IIb trials are significantly more expensive and logistically more difficult than Phase I and Phase IIa trials, which often times delays and limits the progress of malaria vaccine clinical development.

Taking advantage of experience provided by two previous *P. vivax* challenge trials in naïve volunteers [21], [22], a randomized, open-label clinical trial was carried out under laboratory conditions in a small number of semi-immune and malaria-naïve volunteers with the aim of comparing the infection outcome and antibody responses elicited. It was also designed to determine the feasibility and advantages of assessing vaccine protective efficacy in a smaller number of well-characterized volunteers.

## Materials and Methods

### Ethics statement

This trial was conducted according to ICH E-6 Guidelines for Good Clinical Practices [23] and the protocol was approved by Institutional Review Boards (IRB) of the Malaria Vaccine and Drug Development Center–MVDC (CECIV, Cali) and Centro Médico Imbanaco (Cali). Written informed consent (IC) was obtained from each volunteer at enrollment and from *P. vivax*-infected donors. A separate IC was obtained from each volunteer for HIV screening. The clinical trial was registered on clinicaltrials.gov, registry number NCT01585077. The protocol for this trial and the supporting CONSORT checklist are available as supporting information (Checklist S1 and Protocol S1).

### Study participants

**Figure 1:**
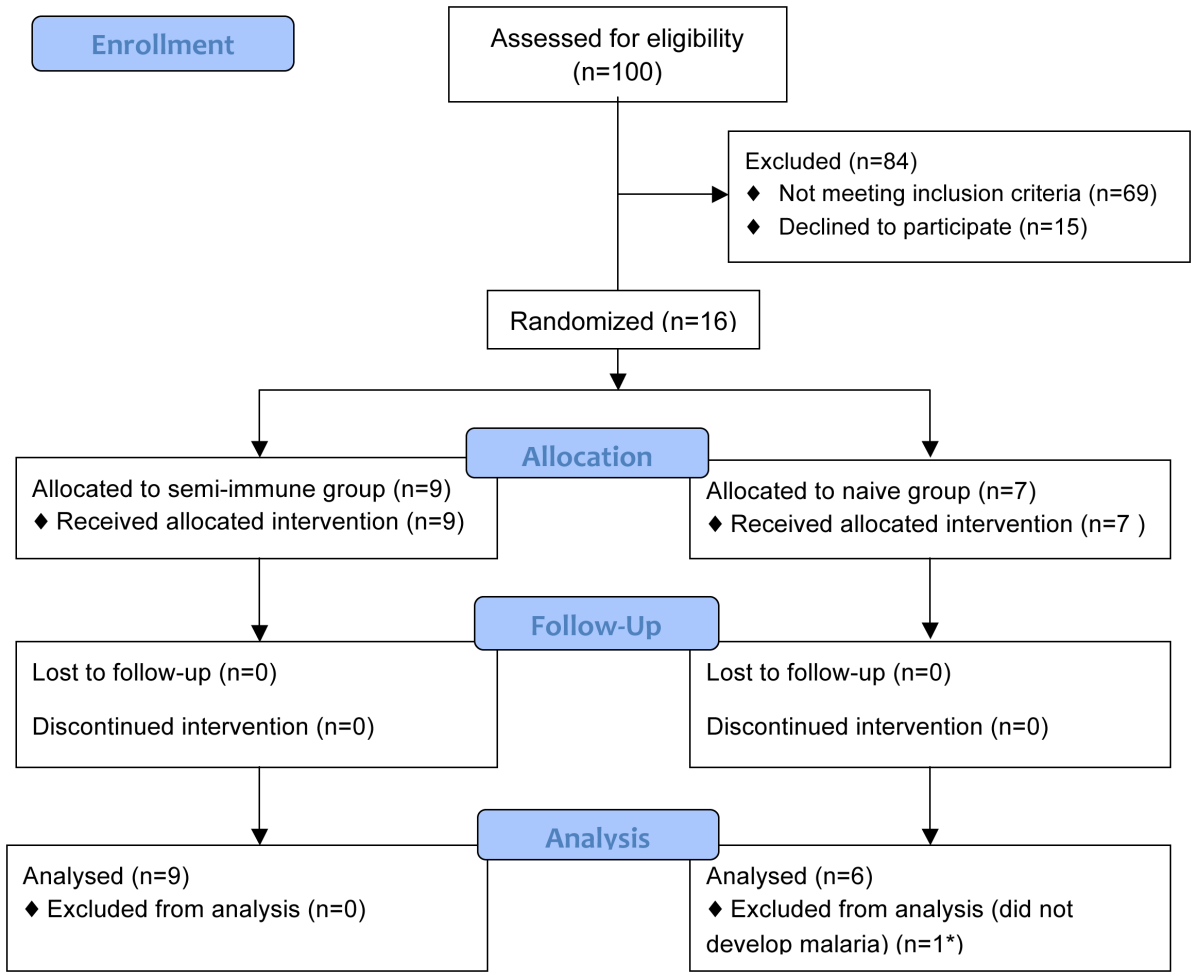
Flow chart of study design and volunteers recruitment.

Sixteen healthy, Duffy-positive (Fy+) male and female volunteers, 18–45 years of age (seven malaria-naïve and nine previously exposed semi-immune volunteers), were recruited for the study (Figure 1). Previous exposure to malaria was confirmed by clinical history as well as by the presence of antibodies against *P. vivax* blood stages by the indirect fluorescent antibody test (IFAT) as described below. Duffy-positive phenotype (Fy+) was confirmed by DNA genotyping [24]. Additionally, six *P. vivax-*infected patients were recruited to serve as a potential parasite donor for mosquito infection.

### Recruitment of study participants

Malaria-naïve volunteers were recruited in Cali (Colombia), a non-endemic city; those with previous malaria experience were recruited in Buenaventura, a malaria-endemic area on the Colombian Pacific Coast. Volunteers were extensively informed about the risks of participation and were provided sufficient opportunity to read the IC forms. Before signing the written consent, all volunteers had to pass an oral or written exam concerning the trial and its risks as described elsewhere [22]. In addition, all were informed about their right to withdraw voluntarily from the study at any time. Exclusion criteria included pregnancy, abnormal laboratory test values, hemoglobin pathology, glucose-6-phosphate dehydrogenase (G6PDH) deficiency, positive for blood bank infectious diseases (syphilis, HIV, Chagas disease, HTLV 1–2, and hepatitis B and hepatitis C) (Table S1), or have any condition that would increase the risk of an adverse outcome, as described in previous studies [21], [22].

### Blood donation

Thirty-five mL of whole blood was collected by venipuncture (Vacutainer tubes, Becton Dickinson, NJ, USA) from six patients diagnosed with *P. vivax* infection after signing a written IC at the out-patient malaria clinic of INSALPA (Buenaventura), and one of them was selected as a parasite donor based on laboratory results and the infection rate of the blood-feed mosquito batch.

Blood samples were collected and distributed as follows: 30 mL (sodium heparin tubes) for mosquito infection [25], and 5 mL (tubes without anticoagulant) for routine screening of common infectious agents (Table S1). Additionally, *P. vivax* mono-infection was confirmed by real-time quantitative polymerase chain reaction (RT-qPCR) as previously reported [26], [27].

### Mosquito infection

*Anopheles albimanus* mosquitoes were reared and infected at the MVDC insectary in Cali as previously described [25]. Batches of fed mosquitoes (∼75) were dissected and microscopically examined for the presence of oocysts in the midgut (day 7) and sporozoites in salivary glands (day 14). Each mosquito's salivary glands were dissected and observed by microscopy at 40X magnification. To estimate the number of sporozoites in salivary glands (sporozoite load), a gland index based on a log-scale was used from +1 (1-10 spz), +2 (1 1–100 spz), +3 (101–1,000 spz), and +4 (>1,000 spz) [26]. Only batches with >50% sporozoite infection rates were considered adequate for sporozoite challenge.

### Sporozoite challenge

Sporozoite challenge of all volunteers was carried out on the same day by exposing volunteers to bites of 2–4 mosquitoes of the same infected batch [21], [28]. Presence of blood in the mosquito's midgut and sporozoites in the salivary glands was confirmed by mosquito dissection after biting. Because a minimum of two infected, mosquito bite-dose was required to induce a reproducible infection [21], [22], more than a single biting cycle may have been necessary. Study participants were under direct observation for one hour after challenge to assess their response to mosquito bites and parasite challenge. Volunteers were then monitored by phone eight hours after challenge and once a day until day four. Thereafter, volunteers were evaluated daily for clinical manifestations and patent parasitemia in an outpatient clinic from days five to 21, and then every second day until day 28.

### Malaria diagnosis and patient follow-up

*P. vivax* infection in challenged volunteers and parasite donor was diagnosed by thick blood smears (TBS), which were examined independently by two experienced microscopists [29]. The criterion for a positive TBS was the identification of at least one morphologically normal malaria parasite confirmed by both microscopists. Presence of an exclusive *P. vivax* infection in the parasite donor was confirmed by *Plasmodium* RT-qPCR [27]. Parasite density was estimated in parasites/µL by counting the number of asexual parasites per 400 white blood cells (WBC) using WBC counts at day 16 after challenge; samples were considered negative after observation of 200 microscopic fields. RT-qPCR was performed for retrospective analysis in a thermal cycler (7,500 Applied Biosystems) as previously reported [27]. Each experiment included the test sample assayed in duplicate, non-infected DNA as negative control, and serial dilution of samples of known *P. vivax* and *P. falciparum* parasitemias for quantification [26]. A sample was considered negative if there was no increase in the fluorescent signal after a minimum of 40 cycles. Detection limit of this technique is one parasite/mL. As soon as parasites were detected by TBS, participants were treated orally with curative doses of chloroquine (1,500 mg chloroquine provided in three doses: 600 mg initially followed by 450 mg doses at 24 and 48 hours) and primaquine (30 mg dose given once per day for 14 days), according to the Colombian government guidelines for malaria treatment [30]. Likewise, during these daily visits, symptoms and signs of malaria were assessed. The severity of adverse events (AE) was scored from 1 to 4 (Grade 1 =  mild, Grade 2 =  moderate, Grade 3 =  severe, and Grade 4 =  life-threatening) as described elsewhere [31].

### Clinical laboratory tests

A comprehensive clinical laboratory screening confirmed the health status of the volunteers one month prior to challenge and at the time of diagnosis, and again at three weeks and four months after treatment was completed. Screening assays consisted of the following: automated whole blood cell counts, urine analysis by dipstick (Multistix 10 SG Reagent Strips, Siemens), blood chemistry tests for creatinine, blood urea nitrogen (BUN), glycemia, total bilirubin, direct bilirubin, transaminases (ALT and AST), coagulation tests, and C-reactive protein.

### Immunological assays

Specific antimalarial antibodies were determined both by IFAT and enzyme-linked immuno-sorbent assay (ELISA) using sera collected from the bleedings described above for clinical laboratory tests. IFAT were performed using *P. vivax* blood-stage antigen preparations derived from infected patients or sporozoites obtained from experimentally infected mosquitoes [32]. ELISA was used to determine the presence of IgG specific to the *P. vivax* circumsporozoite protein (*Pv*CS) and the merozoite surface protein-1 (*Pv*MSP-1) described elsewhere [33]. *Pv*CS corresponded to a chimeric synthetic polypeptide composed of the amino (N) flank, the VK210 and VK247 natural repeat variants, and the carboxyl (C) flanking sequences of the protein [34]; *Pv*MSP-1 corresponded to a recombinant fragment (348 aa) from the N region of the protein, namely r200L [8]. Antibody titers were estimated using serial two-fold dilutions of the test sera, beginning at 1∶200. Optical Density (OD) at 405 nm was measured using a BioTek ELISA Reader (BioTek, Winooski, VT). Results were considered positive when absorbance of the test sera was greater than the cut-off value. Cut-off values were calculated as three SD above the mean absorbance value at 405 nm of negative control sera. Results were expressed as a reactivity index, defined as OD values of test sample divided by the cut-off value.

### Statistical analysis

Study data were collected and managed using REDCap (Nashville, TN, USA) electronic data capture tools [35]; data were subsequently analyzed with the statistical software MATLAB, from MathWorks (Natick, MA, USA). Nominal variables were analyzed using descriptive statistics. Mann-Whitney U or the Kruskal-Wallis tests were used to compare naïve and semi-immune groups and time points, respectively. Fisher's exact test was used to compare proportions. A p value <0.05 was considered as statistically significant. Raw data used for this analysis are available upon request.

## Results

### Study population characteristics

All 16 volunteers were exposed to *P. vivax* sporozoite challenge during June 2013. No age differences were observed among volunteers (ten men and six women), with the mean age of 28 years (range: 19–38) (Table 1).

**Table 1:** Demographic characteristics of the study participants, challenge infective dose, pre-patent period and parasite density after challenge.

Group		Code	Gender	Age (years)	Mosquito bites^a^	Pre-patent period (days)^b^	Parasite density (parasites/µL)^c^	Onset of symptoms (days)^d^
						TBS	RT-qPCR	TBS	RT-qPCR	
		302	M	29	4	13	10	34	1	13
		304^e^	M	26	2	NA	NA	NA	NA	NA*^f^*
		306	M	38	3	13	9	95	1	10
Naïve		310	M	31	3	13	9	110	11	9
		314	F	34	4	12	9	10	24	10
		317	M	33	4	11	9	6	40	8
		319	M	22	4	13	9	38	25	13
										
		301	M	19	4	13	9	55	5	NA
		302	F	32	2	13	9	390	1	NA
		310	M	22	3	13	11	111	2	13
		324	F	36	4	12	11	34	5	8
Semi-immune		327	F	28	3	13	9	25	1	11
		341	F	34	3	13	9	216	1	10
		375	F	21	4	13	9	20	1	NA
		378	M	20	4	13	9	59	1	9
		381	M	37	3	13	9	39	4	10

Most volunteers were men (63%) and ages ranged between 19–38 years old. No significant differences were observed in the pre-patent period of both groups after the infectious challenge with 2-4 bites of infected mosquitoes.

a Number of infected mosquitoes fed on the arm of volunteer; ^b^Pre-patent period defined by positive thick blood smear (TBS); ^c^parasitemia measured at the pre-patent day; ^d^Onset of any symptom, usually weakness or malaise; ^e^No pre-patent period could be determined as the volunteer remained negative for malaria by TBS during the duration of the study. *^f^* no symptoms were reported for those volunteers. NA: Not applicable.

### Sporozoite challenge

The batch of *P. vivax*-infected mosquitoes used for the sporozoite challenge was fed on a blood sample that had a parasite density of 1,273 asexual parasites/µL and 199 gametocytes/µL. Approximately 80% of fed mosquitoes were determined to be infected, displaying an average of 409 sporozoites per salivary glands by day 14 post-blood feeding. Both groups of volunteers completed the biting process within a total period of about four hours. The majority of volunteers (15/16) completed the infective mosquito biting dose (3±1) in a single biting cycle, but one participant required two cycles (Table 1 and Table S2). Two volunteers developed minor, transient discomfort due to pruritus and erythema that disappeared within two days.

### Pre-patent period and parasitemia

**Figure 2:**
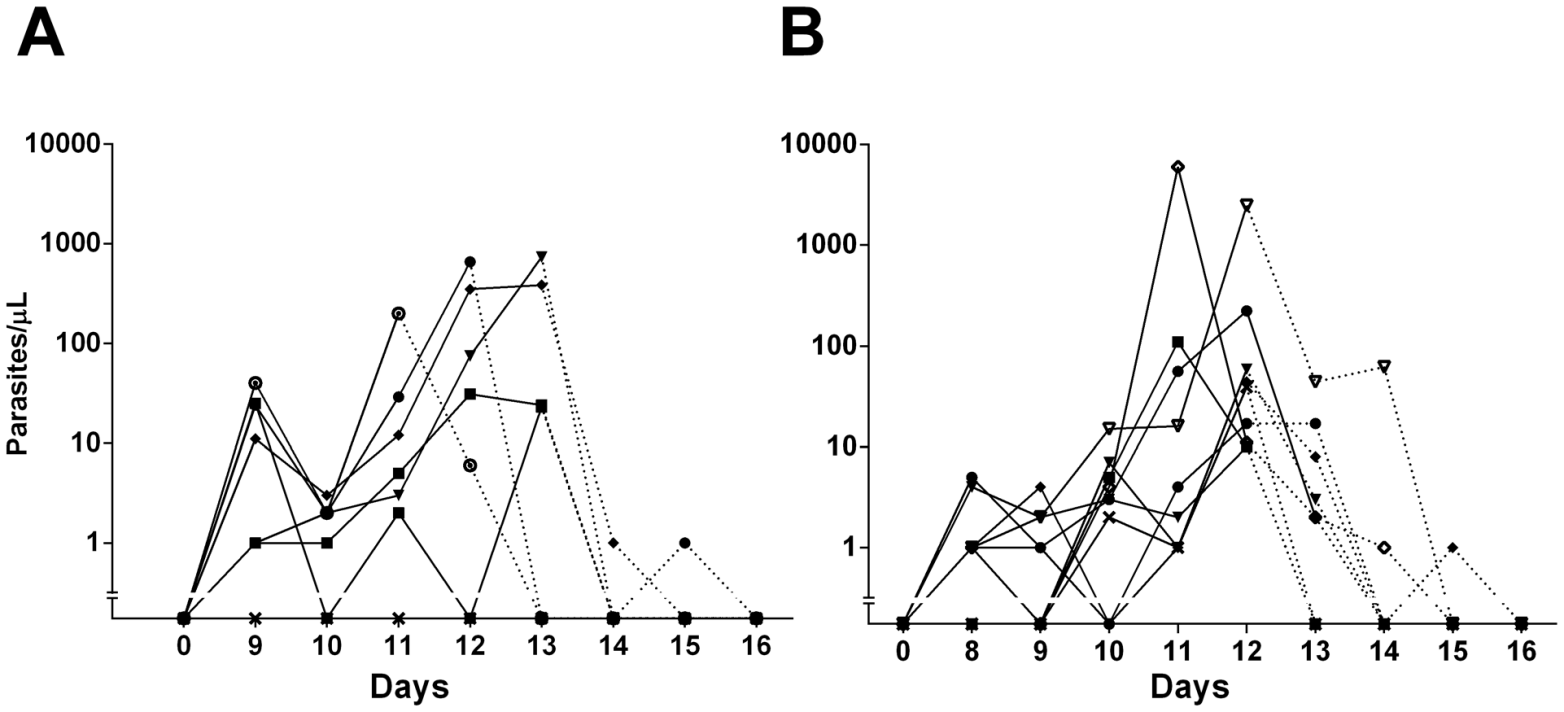
Course of parasitemia determined by RT-qPCR. Parasitemia determined between days 0 and 16 of post-challenge follow-up. Each point represents parasites/µL (Log_10_) in (A) naïve (n = 6) and (B) semi-immune (n = 9) volunteers. Solid lines represent pretreatment and dashed lines post-treatment.

All volunteers developed malaria infection as confirmed by TBS and RT-qPCR, with patent parasitemias developing between days 11 and 13 (mean: 12.7 days) after sporozoite challenge. In naïve volunteers the mean pre-patent period was 12.5±0.8 days (range: 11-13 days), whereas in semi-immune volunteers the period was 12.9±0.3 days (range: 12–13 days). Retrospective RT-qPCR analysis indicated shorter pre-patent periods, 9.2±0.4 and 9.4±0.9 days for naïve and semi-immune volunteers, without significant difference between groups. A naïve volunteer developed transient low-level parasitemia detectable by RT-qPCR by day nine and cured spontaneously in four days. High Performance Liquid Chromatography (HPLC) used for antimalarial drug detection [i.e., chloroquine diphosphate, sulfadoxin, pyrimethamine and mefloquine hydrochloride] was negative in this volunteer (data not shown). Median parasitemia determined by TBS was similar between naïve (36 parasites/µL; IQR 9.0–98.8) and semi-immune volunteers (55 parasites/µL; IQR 29.5–163.5; p = 0.288). RT-qPCR showed that naïve volunteers presented a non-significant higher median parasite density than semi-immune volunteers on day nine, at the time of the first positive RT-qPCR (17.5 parasites/µL; IQR 0.8-28.8 vs. 1.0 parasites/µL; IQR 0.5–2.5; p = 0.087). Median parasite density continued to be higher but not significant in naïve volunteers when parasitemia became patent by microscopy (293 parasites/µL; IQR 29.0–674.3 *vs.* 44; IQR 14.0–167.0 parasites/µL; p = 0.272) (Figure 2).

### Clinical follow-up

Signs and symptoms most frequently observed were fever (body temperature ≥38°C), pallor, headache, nausea, chills, and malaise; all of the aforementioned were more frequent and severe in naïve volunteers. Fever was present in 100% of the naïve and in 33% of the semi-immune volunteers (p = 0.01) (Table 2). Additionally, three semi-immune volunteers developed parasitemias without fever or other symptoms. The remaining semi-immune volunteers presented with symptoms, particularly fever and headache, but symptoms were less intense than those of the naïve group (p = 0.018 and p = 0.001, respectively) (Table 2). All volunteers cleared their parasitemias between 24 and 48 hours after initiating antimalarial treatment, except for a volunteer that remained parasite-positive for 72 hours as confirmed by TBS and RT-qPCR. All volunteers successfully recovered clinically within 2–3 days without any serious AE. None of the volunteers required hospitalization for malaria. A volunteer presented with severe diarrhea and abdominal pain (Grade 4) on day 18 and was diagnosed with parasitic gastroenteritis (hookworms and trichuriasis) and was held under observation for <24 hours in the hospital, requiring intravenous rehydration and pain management. All volunteers were treated with a full course of chloroquine and primaquine at the time of malaria diagnosis, except a volunteer (304N) who remained negative by TBS and was treated by protocol one month after challenge. Some of the AE associated with antimalarial treatment were more frequent in semi-immune as compared to naïve volunteers: insomnia (89% *vs* 67%; p = 0.143); nausea (78% *vs* 50%; p = 0.235); oropharyngeal dryness (89% *vs* 33%; p = 0.011); weakness (67% *vs* 17%; p = 0.041); and pruritus (44% *vs* 17%; p = 0.287).

**Table 2:** Adverse events associated with *P. vivax* infection: frequency, severity and duration in naïve and semi-immune subjects.

	Frequency (%)	Frequency (%)	Frequency (%)	Severity Proportion^b^	Severity Proportion^b^	Severity Proportion^b^	Duration Mean (days)	Duration Mean (days)	Duration Mean (days)
	Naïve	Semi-immune	p-value^a^		Naïve	Semi-immune	p-value^a^		Naïve	Semi-immune	p- value^c^
	(n = 6)	(n = 9)			(n = 6)	(n = 9)			(n = 6)	(n = 9)	
**Symptoms**											
Weakness	6 (100)	6 (67)	0.229		5/6	3/6	0.119		4.26	2.07	0.240
Malaise	6 (100)	5 (56)	0.103		4/6	3/5	0.315		3.97	2.46	0.247
Chills	6 (100)	5 (56)	0.103		3/6	1/5	0.235		2.42	1.13	0.091
Headache	6 (100)	5 (56)	0.103		5/6	0/5	**0.002**		4.8	2.71	0.429
Nausea	6 (100)	5 (56)	0.103		3/6	1/5	0.235		3.37	1.29	0.177
Myalgia	5 (83)	3 (33)	0.119		3/5	1/3	0.235		3.10	1.29	0.250
Arthralgia	4 (67)	2 (22)	0.136		2/4	½	0.525		2.15	1.48	0.800
Dyspnea	2 (33)	1 (11)	0.525		0/2	0/1	NA		1.29	0.10	0.562
Blurred vision	2 (33)	0 (0)	0.143		0/2	0/0	NA		1.90	0.00	NA
**Signs**											
Temperature ≥38°C	6 (100)	3 (33)	**0.028**		3/6	0/3	**0.044**		2.91	1.00	0.095
Tachycardia	4 (67)	2 (22)	0.136		0/4	0/2	NA		2.00	1.08	0.400
Pallor	3 (50)	0 (0)	**0.044**		0/3	0/0	NA		1.00	0.00	NA
Watery eyes	2 (33)	1 (11)	0.525		0/2	0/1	NA		1.19	1.54	0.762
Sweating	2 (33)	0 (0)	0.143		1/2	0/0	0.400		2.11	0.00	NA
Jaundice	1 (17)	0 (0)	0.400		0/1	0/0	NA		0.59	0.00	NA

a p value calculated by Fisher's exact test. Significant p values are shown in bold. ^b^Proportion of patients presenting severe (Grade 3) signs and symptoms as defined by FDA guidelines [31]. ^c^p value calculated by Mann-Whitney test. NA: not applicable. Significant p values are shown in bold.

Three months after treatment, a naïve volunteer presented with a *P. vivax* infection after being in a malaria-endemic area. The volunteer was treated with standard antimalarial therapy and successfully recovered, and has not presented a new infection as of today.

### Clinical laboratory follow-up

All baseline clinical laboratory tests were normal before sporozoite challenge; however some biochemistry laboratory results showed abnormal values at the time of malaria diagnosis in a few volunteers with no significant difference (Table 3). A naïve volunteer (310N) showed severe hyperbilirubinemia (Direct bilirubin: 1.76 mg/dL, normal value: 0.1–0.3 mg /dL) and moderate AST and ALT levels (120 IU/ml, 136 IU/ml respectively). Two more volunteers (314N and 341P) showed severe alteration in AST levels, (149 and 159 IU/ml, respectively) and ALT levels (237 IU/ml and 205 IU/ml, respectively). In terms of hematology tests, three naïve volunteers (302N, 306N and 310N) presented with moderate or severe thrombocytopenia (130×10^3^, 110×10^3^ and 96×10^3^ platelets/µL, respectively) and four volunteers had mild (302N: 3.4×10^3^ cell/µL) to moderate leukopenia (306N, 310N, and 317N: 2.2×10^3^, 2.2×10^3^, 2.3×10^3^ cell/µL, respectively). In contrast, none of the semi-immunes presented with thrombocytopenia and only one presented with mild leukopenia (327P: 3.3×10^3^ cell/µL). Hematological and biochemical alterations resolved spontaneously after antimalarial treatment.

**Table 3:** Naïve and semi-immune volunteers with laboratory abnormalities at the time of malaria diagnosis.

	Volunteers No. (%)	
Parameter/unit	Grade^a^	Naïve	Semi-immune	p value^b^
**Blood chemistry**				
AST (IU/L)				
	None	4 (66.7)	7 (77.8)	1.000
	Mild	0 (0)	1 (11.1)	1.000
	Moderate	1 (16.7)	0 (0)	0.400
	Severe	1 (16.7)	1 (11.1)	1.000
ALT (IU/L)				
	None	3 (50.0)	7 (77.8)	0.329
	Mild	2 (33.3)	1 (11.1)	0.525
	Moderate	1 (16.7)	1 (11.1)	1.000
	Severe	0 (0)	0 (0)	NA
**Hematology**				
Hemoglobin (g/dL)				
	None	6 (100)	9 (100)	NA
	Mild	0 (0)	0 (0)	NA
	Moderate	0 (0)	0 (0)	NA
	Severe	0 (0)	0 (0)	NA
Platelets (×10^3^/µL)				
	None	3 (50.0)	9 100	0.044
	Mild	2 (33.3)	0 (0)	0.400
	Moderate	1 (16.7)	0 (0)	0.400
WBC (×10^3^/µL)				
	None	2 (33.3)	8 (88.9)	0.089
	Mild	1 (16.7)	1 (11.1)	1.000
	Moderate	3 (50.0)	0 (0)	0.044
	Severe	0 (0)	0 (0)	NA

a Grade according to FDA Guidelines [31]. ^b^p values calculated by Fisher's exact test. Significant p values are shown in bold. Abbreviations: AST, Aspartate Aminotransferase, ALT, alanine aminotransferase. NA: not applicable.

### Antibody responses

As shown in Table 4, IgG antibodies to sporozoites or blood stage proteins were confirmed by IFAT in all volunteers from the endemic area, although at low antibody levels (1∶20 to 1∶80). After parasite challenge, 5/7 naive and 8/9 semi-immune showed an increase in antibody titers against blood stages, which remained similar for three weeks post-treatment in semi-immune volunteers. Only three naïve volunteers showed minor increases in antibody levels after three weeks; three semi-immune participants remained positive after four months post-treatment (data not shown).

**Table 4:** Antibody titer of naive and semi-immune volunteers against blood-stage and sporozoite-stage antigens as assessed by immunofluorescence (IFAT) before challenge, on the day of diagnosis, and 3 weeks after receiving antimalarial treatment.

		IFAT	IFAT	IFAT		IFAT	IFAT	IFAT
		Sporozoites	Sporozoites	Sporozoites		Blood stages	Blood stages	Blood stages
Group	Code	Pre-challenge	Diagnosis	Post-treatment		Pre-challenge	Diagnosis	Post-treatment
	**302**	<1∶20^a^	<1∶20	<1∶20		<1∶20	1∶40	1∶40
	**304** ***^b^***	<1∶20	<1∶20	<1∶20		<1∶20	<1∶20	<1∶20
	**306**	<1∶20	<1∶20	1∶40		<1∶20	1∶40	1∶80
**Naïve**	**310**	<1∶20	1∶20	<1∶20		<1∶20	1∶20	<1∶20
	**314**	<1∶20	<1∶20	1∶80		<1∶20	1∶20	1∶80
	**317**	<1∶20	<1∶20	<1∶20		<1∶20	1∶20	<1∶20
	**319**	<1∶20	<1∶20	<1∶20		<1∶20	<1∶20	<1∶20
								
	**301**	<1∶20	<1∶20	<1∶20		1∶20	<1∶20	1∶20
	**302**	<1∶20	<1∶20	<1∶20		1∶20	1∶40	1∶80
	**310**	<1∶20	<1∶20	<1∶20		1∶20	1∶20	1∶20
	**324**	<1∶20	1∶80	1∶20		1∶20	1∶20	<1∶20
**Semi-immune**	**327**	<1∶20	<1∶20	<1∶20		1∶20	1∶20	1∶40
	**341**	<1∶20	<1∶20	<1∶20		1∶20	1∶20	1∶40
	**375**	<1∶20	<1∶20	1∶20		1∶20	1∶20	<1∶20
	**378**	<1∶20	<1∶20	<1∶20		1∶20	1∶20	1∶20
	**381**	1∶80	<1∶20	<1∶20		1∶20	1∶20	1∶20

a Negative at 1∶20 dilution; *^b^* this volunteer recovered from submicroscopic parasitemia without antimalarial treatment.

**Figure 3:**
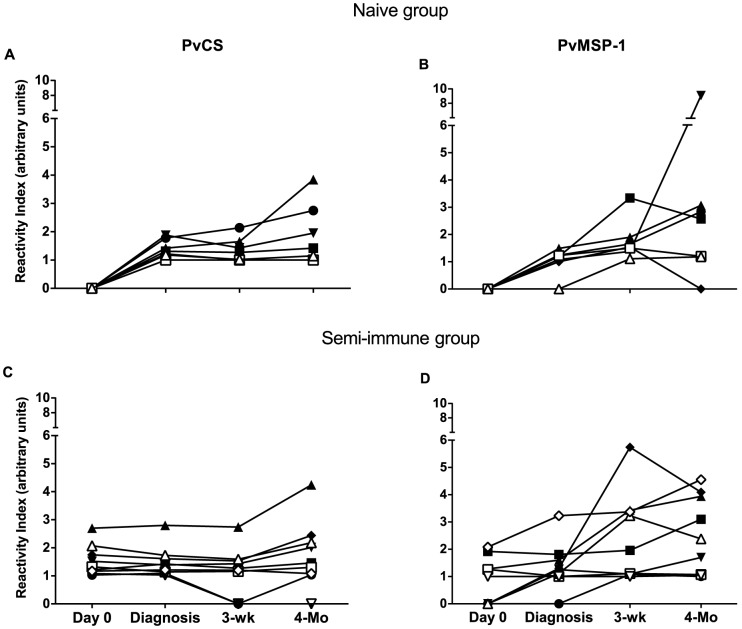
Specific antibody responses against *P. vivax* antigens in naïve and semi-immune volunteers. Antibodies are expressed as reactivity index defined as OD values of tested sample divided by the cut-off value. Reactivity indexes against (A) *Pvs*Cs and (B) *Pv*MSP-1 in naïve volunteers (n = 7). Reactivity indexes against (C) *Pvs*CS and (D) *Pv*MSP-1 in semi-immune volunteers (n = 9).

At enrollment, 100% of the semi-immune volunteers had antibodies to *Pv*CS and 45% to *Pv*MSP-1. In the group of naïve volunteers, 5/7 developed specific antibodies to *Pv*CS and *Pv*MSP-1 antigens by the day of parasite patency; the entire group (7/7) became positive to *Pv*MSP-1 by three weeks after challenge. A total of 6/9 (67%) and 8/9 (89%) of the semi-immune volunteers presented with or showed increases in anti-*Pv*MSP-1 antibody levels at diagnosis and three weeks after challenge, respectively. In contrast, 5/7 naïve (71%) and 7/9 (78%) semi-immune volunteers presented with antibodies to *Pv*CS three weeks after challenge. The percentage of positive naïve or semi-immune volunteers was similar after four months of follow-up. No differences in the frequency of responders were observed between the two groups after challenge. Antibody responses expressed as a reactivity index (RI) showed a significant increase between the pre-challenge day and four months after treatment in naïve volunteers with a mean value from 0 to 1.9, and 0 to 2.9 for *Pv*CS and *Pv*MSP-1, respectively. In semi-immune volunteers, the RI was similar for responses against the *Pv*CS (from 1.5 to 1.8), whereas it increased in response to *Pv*MSP-1 (0.8 to 2.5) at the same set points, although no significant differences were observed (Figure 3).

## Discussion

This study confirms the findings of previous trials in which naïve volunteers could be safely and reproducibly infected by the bites of a small number of *An. albimanus* mosquitoes carrying mature sporozoites [21], [22]. Sporozoite infectious challenge was also safe and reproducible in semi-immune volunteers. In previous trials mosquito biting doses ranged from 2-10, inducing pre-patent periods ranging between 9 and 15 days in naïve volunteers [21], [22]; whereas in this study at least 2–4 bites induced patent infections in all volunteers in 11–13 days as determined by microscopy. It was also reproducible in that infections could be confirmed by RT-qPCR beginning at day nine.

For logistical convenience, semi-immune volunteers were recruited in Buenaventura, a low malaria transmission region near Cali. All volunteers from this area reported to have experienced previous malaria episodes (2–5 times) and presented with low antimalarial antibody titers at the time of enrollment, suggesting that the frequency of previous malaria exposure and concomitant immunity were low. Therefore, it was not surprising that volunteers in both groups developed similar pre-patent periods, although it was interesting that both the clinical outcome of the infection and specific antibody responses in semi-immune and naïve volunteers were different. However, no association between onset of symptoms and antibody levels or parasitemia was found.

Both naïve and semi-immune volunteers presented with similar parasite densities (20–400 parasites/µL), but infections in naïve volunteers resulted in a significantly higher rate of severe headache and fever. In the scoring of AE, a bias may have occurred because symptoms were self-reported by volunteers; it is likely that both groups had different thresholds or responses to symptoms. It is intriguing that despite the low antibody levels and limited exposure to malaria, volunteers from Buenaventura had already developed a significant level of clinical immunity. Until a decade ago Buenaventura was among the regions with the highest malaria transmission in Colombia; however in the last few years, transmission has significantly decreased (∼80–90%) [36]. Because semi-immune volunteers had a mean age of 27 years, their potential exposure may have been limited [37]. In contrast to more highly endemic regions, where malaria is concentrated in children, exposure to malaria in Colombia is more frequent in young adults [38], which may explain why some volunteers had relatively low levels of immunity.

Due to the short duration of infection and possibly the low-density parasite inoculum, specific antibody responses to the parasite blood stages as well as to *Pv*CS and *Pv*MSP-1 proteins were low. The sporozoite inoculum during an *Anopheles* mosquito infectious-bite does not appear to depend on the mosquito sporozoite load. It is likely that 2–4 bites of *An. albimanus,* considered a *“*less efficient” vector than African or Asian *Anopheles* species, delivers only a limited number of sporozoites [39], [40], which in any case will have a brief exposure to the immune system. Nevertheless, this inoculum was sufficient to induce detectable anti-*Pv*CS and anti-sporozoite antibodies in all naïve volunteers, although not enough to significantly boost the pre-existing antibody titers in the semi-immune group. Likewise, boosting of antibody responses to asexual blood-stage parasites may have also been compromised due to the careful monitoring and follow-up of volunteers with prompt curative treatment. Therefore, it is most likely that the milder clinical manifestations in semi-immune volunteers may have been due to the lower initial parasitemias detected by RT-qPCR but also possible to immune responses to parasite components other than *Pv*CS and *Pv*MSP1, including cytokines and innate immune responses. C-reactive protein measurements at the time of malaria diagnosis showed higher acute inflammatory responses in naïve compared to semi-immune volunteers (57.7 mg/dL *vs* 17.6 mg/dL; p = 0.036), which appears to be associated with clinical findings in naïve volunteers. A comprehensive transcriptome analysis is being planned and would be the subject of further research. Antibody responses to asexual blood stage parasites and *Pv*MSP-1, which are associated with a reduction in clinical manifestations [41], developed a more homogeneous profile and were consistently stronger than those to sporozoite antigens (Figure 3). The potential impact on such protective responses in the evolution of parasitemia and clinical manifestations could not be determined, as the study protocol was designed to treat all volunteers as soon as parasitemias became patent upon microscopic examination.

It was also interesting to note that despite timely treatment, half of the naïve volunteers developed significant alterations in their hematological profiles, including thrombocytopenia and leukopenia, whereas only one semi-immune volunteer presented with mild leukopenia. It is known that *P. vivax* frequently induces thrombocytopenia which may be considered as predictive marker of malaria infection [42]. The hematological alterations observed were similar to those previously reported in malaria-naïve volunteers experimentally infected by the bite of *P. falciparum*-infected mosquitoes [16]. In contrast to previous challenge infection trials where a few volunteers had mild alterations in transaminase values [21], [22], the most frequent finding in the present trial was mild to severe transaminase abnormalities. However, none of the volunteers displayed clinical manifestations related to an alteration in liver function, except for a volunteer that presented hyperbilirubinemia, suggesting a degree of hepatocellular pathology.

According to FDA guidelines some laboratory test abnormalities could be classified as moderate or severe [31], but none were considered potentially life-threatening. These results are consistent with the safety of a previously reported infective challenge model [21], [22].

As in previous trials, parasitemias were cleared in most cases (13/15) within the first 24 hours of treatment in both groups [21], [22]. The case of the naïve volunteer who developed an unexpectedly low and transient parasitemia, and who had been previously classified as Fy+ by PCR, is unclear. We speculate that it might be explained by Duffy antigen polymorphism, but this requires further analysis [43]. We have no explanation for the AE reported by semi-immune volunteers upon antimalarial treatment, that in all cases were more frequent than in naïve volunteers. Regarding the volunteer who presented *P. vivax* infection three months after treatment, he reported to having been in Buenaventura, the same area where parasites used in the challenge were collected. Thus, whether this infection was a relapse or reinfection could not be confirmed.

Despite the great logistical challenges of this study, it's the potential usefulness of the mosquito challenge infection system could serve to accelerate the transition from Phase IIa to Phase IIb malaria vaccine trials. Furthermore, the model described here represents a cost- effective method for proof-of-principle malaria vaccine efficacy studies under conditions approximating Phase IIb. This phase is logistically and economically demanding as it requires well characterized endemic settings with sufficient malaria transmission to ensure that volunteers are exposed to natural mosquito challenge within a reasonable time. In addition, volunteers get exposed to undetermined genotypic/biological differences in circulating wild parasite isolates [44]–[46]. The model described here would substantially simplify and diminish the cost of transition from Phase IIa to Phase IIb vaccine trials, as well as facilitate the study of protective immune responses elicited by *P. vivax* vaccines in volunteers previously exposed to malaria.

In conclusion, the safety, reproducibility, and the narrow pre-patent window in this model would allow the use of relatively smaller-sized experimental groups to determine differences between control and immunized volunteers. The model should allow immediate testing of both pre-erythrocytic and asexual blood stages antigens in Phase II trials as well as human vaccination with *P. vivax* irradiated-sporozoites. A controlled, size-limited Phase IIb vaccine study to evaluate the protective efficacy of a *Pv*CS-based vaccine candidate is currently under development. Moreover, the model could contribute to efficacy studies on new antimalarial compounds, especially those with potential effects on *P. vivax* liver stages.

## Supporting Information

Checklist S1 **CONSORT 2010 checklist of information to include when reporting a randomised trial.**
(DOC)

Table S1 **Screening of common infectious agents for parasite donors and recruited volunteers.**
(DOCX)

Table S2 **Sporozoite challenge summary.**
(DOCX)

Protocol S1 **Comparison of the susceptibility of naïve and pre-immune volunteers to the Infectious challenge with viable** ***Plasmodium vivax*** **sporozoites.**
(DOCX)
