# Supplementary material for: Plasmodium vivax Sporozoite Challenge in Malaria-Naïve and Semi-Immune Colombian Volunteers
Source: PLoS One. 2014 Jun 25;9(6):e99754. doi: 10.1371/journal.pone.0099754 (PMC4070897; doi:10.1371/journal.pone.0099754)
Supplement: Table S1 — Screening of common infectious agents for parasite donors and recruited volunteers. (DOCX) [file pone.0099754.s002.docx]

**Table S1.** Screening of common infectious agents for parasite donors and recruited volunteers*^a^*.

| **Disease** | **Pathogen** | **Technique** | **Reference values** | **Commercial brand** |
| --- | --- | --- | --- | --- |
| Hepatitis B Ag | HB virus (HBs Ag) | Immunochromatography | Negative | Standard Diagnostics |
| Hepatitis C | HC virus | Immunochromatography | Negative | Standard Diagnostics |
| AIDS | HIV*^b^* | Immunochromatography | Negative | Standard Diagnostics |
| TSP | HTLV I/HTLV II | Inmunocomb | Negative | Orgenics/Biosystems |
| Syphilis | *Treponema pallidum* | RPR Carbon | No reactive | Biosystems |
| Chagas | *Trypanosoma cruzi* | Immunochromatography | Negative | Standard Diagnostics |

HBs Ag: Hepatitis B surface antigen; TSP, tropical spastic paraparesis.

*^a^*All tests have external monthly quality control by Progba/Cemic Bs Argentina. *^b^* confirmatory tests are performed in external laboratory using Western Blot.
