# Supplementary material for: Plasmodium vivax Sporozoite Challenge in Malaria-Naïve and Semi-Immune Colombian Volunteers
Source: PLoS One. 2014 Jun 25;9(6):e99754. doi: 10.1371/journal.pone.0099754 (PMC4070897; doi:10.1371/journal.pone.0099754)
Supplement: Table S2 — Sporozoite challenge summary. (DOCX) [file pone.0099754.s003.docx]

**Table S2.** Sporozoite challenge summary

| **Group** | **Code** | **Mosquito exposition*^a^*** | **Blood-feeding positive mosquitoes*^b^*** | **Sporozoites positive mosquitoes*^c^*** |
| --- | --- | --- | --- | --- |
| **Naive** | **304** | 4 | 4 | 75% |
|  | **306** | 4 | 3 | 100% |
|  | **302** | 4 | 4 | 100% |
|  | **310** | 4 | 3 | 100% |
|  | **317** | 4 | 4 | 100% |
|  | **314** | 4 | 4 | 100% |
|  | **319** | 4 | 4 | 100% |
| **Semi- immune** | **310** | 4 | 4 | 75% |
|  | **302** | 4 | 3 | 68% |
|  | **324*^d^*** | 7 | 7 | 57% |
|  | **375** | 4 | 4 | 100% |
|  | **381** | 4 | 4 | 75% |
|  | **327** | 4 | 4 | 75% |
|  | **341** | 4 | 3 | 100% |
|  | **378** | 4 | 4 | 100% |
|  | **301** | 4 | 4 | 100% |

^a^ Number of mosquitoes each participant was exposed; *^b^* number of mosquitoes that fed on each participant; *^c^* Percentage of mosquitos positive for sporozoites in salivary glands. *^d^* a second biting cycle was required
