# Supplementary material for: Plasmodium vivax Sporozoite Challenge in Malaria-Naïve and Semi-Immune Colombian Volunteers
Source: PLoS One. 2014 Jun 25;9(6):e99754. doi: 10.1371/journal.pone.0099754 (PMC4070897; doi:10.1371/journal.pone.0099754)
Supplement: Protocol S1 — Comparison of the susceptibility of naïve and pre-immune volunteers to the Infectious challenge with viable Plasmodium vivax sporozoites. (DOCX) [file pone.0099754.s004.docx]

**Protocol Title: Comparison of the Susceptibility of Naïve and Pre-immune Volunteers to the Infectious Challenge with Viable *Plasmodium vivax* Sporozoites**

Protocol number: **CIV-008-102010**

Principal Investigator: Sócrates Herrera Valencia, MD

Director

Malaria Vaccine and Drug Development Center (MVDC)

*Centro Internacional de Vacunas* (*CIV*)

Carrera 37 2Bis No. 5E-08, Cali, Colombia Phone: (57)-(2)-558 3921/37 Ext.102

Fax: (57)-(2)-5560141

[sherrera@inmuno.org](mailto:sherrera@inmuno.org)

Co-Investigators: Myriam Arévalo-Herrera, PhD

Scientific Director

Malaria Vaccine and Drug Development Center (MVDC)

*Centro Internacional de Vacunas* (*CIV*)

Carrera 37 2Bis No. 5E-08, Cali, Colombia

Phone: (57)-(2)-558 3921/37 Ext.102

Fax: (57)-(2)-5560141

[marevalo@inmuno.org](mailto:marevalo@inmuno.org)

Ernesto Martínez Buitrago, MD

*Centro Médico Imbanaco*

Carrera 38, 5A-100, Cali, Valle del Cauca

Phone: 5560086

José Millán Oñate, MD

*Centro Médico Imbanaco*

Carrera 38, 5A-100, Cali, Valle del Cauca

Phone: (57)-(2)-5560086

Clinical Monitor: Ricardo Palacios, MD, PhD

Meridional R&D

Rua Fernão Dias, 128*/*34A

São Paulo, SP, Brasil

CEP 05427-000

Phone: +55(11)939 40670 [rpalacios@meridionalrd.com](mailto:ricardopalacios@gmx.net)

Institutional Review Boards: Ethics Committee of MVDC

(*Cómite de Ética Centro Internacional de Vacunas*)

IRB# IRB00007039 - IRB00007040

FWA: FWA00016072

Phone: (57)-(2)-518-5677

Fax: (57)-(2)—554284

[rodryospi@gmail.com](mailto:rodryospi@gmail.com)

Ethics Committee of *Centro Médico Imbanaco* Carrera 38 A No. 5 A 100, Cali, Colombia

Phone: (57)-(2)-6821000

Fax (57)-(2)-5186000

Estimated number of admitted volunteers:

Step (A) Blood donation:

Minimum 5, Maximum 15

Step (B) Challenge:

19 (plus 4-6 alternative volunteers)

Clinical Laboratory: Asoclinic Inmunología Ltda.

Cra. 37 2 Bis 5E-08 Templete Cali, Colombia

Phone: (57)-(2) - 5574929

1. SYNOPSIS

| **Study Design** | This is a randomized clinical trial designed to compare the susceptibility of naïve and pre-immune volunteers to the infectious challenge with viable *Plasmodium vivax* sporozoites (*the term pre-immune hereafter denotes only previous infection with this parasite and not a defined immunity state of the volunteer to malaria infection*)*.* The development of this study will allow establishing a protocol to evaluate the efficacy of *P. vivax* vaccine candidates in subsequent Phase IIa and Phase IIb clinical studies. The study will be divided in two successive steps:  **Step A - Donation of Parasitized Blood:** The volunteers will be patients with active *P. vivax* infection that agree to donate infected blood. *P. vivax*-infected blood samples will be collected and analyzed according to internationally standardized blood bank procedures to determine the possible presence of co-infections. The parasitized blood will be used to feed *Anopheles albimanus* mosquitoes using an Artificial Membrane Feeding Assay (MFA) technique. Fourteen days (14) after blood feeding, a positive mosquito lot will be selected for Step B.  **Step B - Challenge:** A total of 19 healthy volunteers will be enrolled in the study after completing the informed consent (IC) process, formalized in writing through an IC form signed by the volunteer in the presence of two witnesses. The volunteers will be assigned to one of the following two groups: One group of 7 volunteers (n=7) without previous exposure to malaria (naïve volunteers) and another group of twelve volunteers (n=12) that have had previous episodes of malaria, as determined by clinical history and serological tests. These volunteers will be subjected to infectious challenge through the bites of 3±1 experimentally-infected *P. vivax* mosquitoes. Volunteers will be strictly followed up after the infectious challenge and will be treated as soon as infection is detected in peripheral blood by thick blood smear (TBS). The comparison of the results obtained in these two groups of volunteers will determine the reproducibility of the challenge model previously established by our group and will allow studying the effect of the natural immune response on parasite development. |
| --- | --- |
| **Study**  **objectives** | **Primary objective: Compare the susceptibility of naïve and pre-immune volunteers to infectious challenge with viable *Plasmodium vivax* sporozoites.**  **Secondary objectives:**  • Establish an experimental model of infection with viable *P. vivax* sporozoites in individuals previously exposed to malaria in endemic areas.  • Evaluate and compare the pre-patent period induced in naïve and pre-immune volunteers.  • Determine the clinical characteristics of the disease in the two groups of volunteers.  • Compare the immune response of the volunteers after experimental exposure to the parasite. |
| **Places where the study will be conducted** | **MVDC**  Carrera 37 2Bis No. 5E-08  Cali, Colombia  Phone: (57)-(2)-5574929-5574921  Fax: (57)-(2)-5560141  Clinical Trials Unit  • Site for volunteers recruitment.  • TBS malaria diagnosis.  Clinical Laboratory  • Laboratory screening tests: blood chemistry, hematology tests and urinalysis.  • Laboratory tests for malaria diagnosis by TBS.  • Laboratory tests for *P. vivax* serology (IFAT).  ***Centro de Biotecnología Aplicada* (*CBA)***  **Center of Applied Biotechnology (CAB)**  Km. 6 Vía Cali- Puerto Tejada.  Corregimiento el Hormiguero  Cali, Valle del Cauca  Phone: (57) (2) 521 6228/ 521 4060  Entomology Unit:  • Site for the challenge with infected mosquitoes. |
| **Study Duration** | 6 months. |
| **Purpose of the Study** | To compare the susceptibility of naïve and pre-immune volunteers to the infectious challenge with viable *P. vivax* sporozoites. |
| **Step A:**  **Eligibility Criteria for Blood Donors** | **Inclusion criteria**  • Men or non-pregnant women over 15 and under 60 years of age.  • Not diagnosed with chronic or acute diseases, whether infectious or not, which compromise any of the following systems: respiratory, cardiovascular, gastrointestinal, hepatic, renal, neurological, musculoskeletal, genitourinary, hematopoietic or psychiatric diseases diagnosed by a specialist.  • Diagnosis of *P. vivax* malaria by TBS.  • Negative diagnosis for *P. falciparum* and *P. malariae* malaria by either TBS or PCR.  • Parasitemia of ≥ 0.1%, as determined by TBS.  • Not having received anti-malarial treatment before TBS diagnosis.  • Be able of providing free and voluntary informed consent or informed assent (in the case of participants between 15 and 17 years of age).  **Exclusion criteria**  • To be under 15 and over 60 years of age.  • Have or have had a disease or clinical condition, which at the criteria of the medical researchers, increases the risk associated with participation in this study,  • Having received transfusion of any blood component within the 6 (six) months prior to the study.   - To have a hemoglobin (Hb) level < 9 g/dL. - To be participating as volunteer in other research studies. |
| **Step B: Eligibility Criteria for Volunteers of the Sporozoite Challenge** | **Inclusion criteria for naive volunteers:**  • Healthy men or non-pregnant women within 18–45 years of age.  • To have completed the IC process freely and voluntarily in the presence of two witnesses, who also signed the IC form.   - To show sufficient understanding of the clinical trial, as evidenced by the correct answer of a questionnaire.   • No history of malaria infection.   - Having no history of chronic or acute diseases, whether infectious or not, which affect one of the following systems: respiratory, cardiovascular, gastrointestinal, hepatic, renal, neurological, musculoskeletal, genitourinary, hematopoietic or psychiatric diseases diagnosed by a specialist. - Participating women must agree to use adequate contraception starting a month before the challenge for up to three months after the study completion.   • Accepting not to travel outside the study area from the seventh (7^th^) to the thirty first (31^st^) day post-challenge, which is the period at which there is higher risk to develop parasitemia.  • Accepting not to travel to areas considered endemic for malaria during the study period (i.e. Buenaventura, Tierralta, Quibdó, Tumaco, Urabá and Bajo Cauca).  • To be reachable by phone during the entire study period.  • To be Duffy positive.  • To have Hb levels > 11 g/dL.  • Being available to participate during the period in which the study is scheduled.  **Exclusion criteria for naive volunteers:**  • To be <18 or > 45 years of age.  • To be pregnant (as confirmed by laboratory test), breast-feeding, planning to be pregnant from the time of enrollment for up to six months after the challenge.  • To be Duffy negative red blood cell phenotype.  • To have G6PD deficiency.  • To have any hemoglobin pathology  • To have a personal and family history of allergies to drugs or insect bites.  • To have a history of malaria infection or have received vaccination against malaria.  • To have clinical or laboratory abnormalities identified by (the) investigator(s).  • *P. vivax* IFAT titers >1:20 in the screening tests.  • To have lived in an endemic area for malaria during the 6 months prior to the challenge.  • To have clinical or laboratory evidence of a systemic disease, including psychiatric, hepatic, cardiovascular, pulmonary, renal diseases or any other disease that can have a negative impact and affect the results of the study.   - To have evidence of active Hepatitis B, Hepatitis C or HIV infection. - To have received a transfusion of any blood component within the 6 (six) months prior to the study.   • Planning to have surgery within the time of recruitment and the post-challenge follow up period.  • To have an autoimmune disease (Lupus, rheumatoid arthritis, thyroiditis or other).  • To be splenectomized.  • To be under treatment with drugs that act on the immune system (steroids, immune-suppressive or immune-modulatory agents).  • To have a history of alcoholism or drug abuse, defined as a habit that interferes with normal social functioning.  • To have a condition that may interfere with the ability to provide free and voluntary informed consent.   - To be participating as a volunteer in other research studies.   **Inclusion criteria for pre-immune volunteers:**  • Healthy men and non-pregnant women within 18–45 years of age.  • To complete the IC process freely and voluntarily in the company of two witnesses, who also signed the IC form.  • Having a history of *P. vivax* infection within the last 12 months, as determined by medical history and serological tests.   - Having no history of chronic or acute diseases, whether infectious or not, which affect one of the following systems: respiratory, cardiovascular, gastrointestinal, hepatic, renal, neurological, musculoskeletal, genitourinary, hematopoietic or psychiatric diseases diagnosed by a specialist.   • Woman volunteers must agree to use adequate contraception starting a month before the challenge until three months after the challenge.  • Accepting not to travel outside the study area from day seven (7) to day 31 post-challenge (the period of highest risk of developing parasitemia).  • Accepting not to travel to areas considered endemic for malaria during the study period (Buenaventura, Tierralta, Quibdó, Tumaco, Urabá and Bajo Cauca).  • Being reachable by phone during the entire study period.  • Being available to participate during the period in which the study is scheduled. |

2. TABLE OF CONTENTS

[1. SYNOPSIS 3](#_Toc383288807)

[2. TABLE OF CONTENTS 9](#_Toc383288808)

[3. LIST OF ABBREVIATIONS 13](#_Toc383288809)

[4. BACKROUND AND RATIONALE 15](#_Toc383288810)

[4.1. BACKGROUND OF EXPERIMENTAL CHALLENGE WITH MALARIA SPOROZOITES 18](#_Toc383288811)

[4.1.1. Background of Experimental Infectious Challenges with *P. falciparum* sporozoites 18](#_Toc383288812)

[4.1.2. Background of Experimental Infectious Challenges with *P. vivax* sporozoites 20](#_Toc383288813)

[4.2. RATONALE FOR COMPARING THE *P. vivax* SPOROZOITE CHALLENGE IN NAIVE AND PRE-IMMUNE INDIVIDUALS 22](#_Toc383288814)

[4.3. RATIONALE FOR CONDUCTING THIS STUDY IN COLOMBIA 23](#_Toc383288815)

[4.3.1. Epidemiology 23](#_Toc383288816)

[4.3.2. Morbidity and Mortality of *P. vivax* infection in Colombia 24](#_Toc383288817)

[4.4. BACKGROUND OF THE SCIENTIFIC GROUP AND FACILITIES OF THE RESEARCH SITES 25](#_Toc383288818)

[4.4.1. Malaria Vaccine and Drug Development Center (MVDC) 25](#_Toc383288819)

[4.4.2. Caucaseco Scientific Research Center (Caucaseco) 26](#_Toc383288820)

[4.4.3. Center of Applied Biotechnology (CAB) 26](#_Toc383288821)

[5. PRELIMINARY DATA FROM THE MVDC ENTOMOLOGY UNIT 27](#_Toc383288822)

[6. HYPOTHESIS 27](#_Toc383288823)

[7. STUDY OBJECTIVES 27](#_Toc383288824)

[7.2. SECONDARY OBJECTIVES 27](#_Toc383288825)

[8. STUDY DESIGN 29](#_Toc383288826)

[8.1. CLINICAL TRIAL PLAN 30](#_Toc383288827)

[8.1.1. Step A: Donation of *P. vivax*-infected Blood 30](#_Toc383288828)

[8.1.1.1. Selection of Volunteers 30](#_Toc383288829)

[8.1.1.2. Sample Size 30](#_Toc383288830)

[8.1.1.3. Inclusion Criteria 30](#_Toc383288831)

[8.1.1.4. Exclusion Criteria 31](#_Toc383288832)

[8.1.1.5. Study Procedures 32](#_Toc383288833)

[8.1.1.5.1. Informed Consent 32](#_Toc383288834)

[8.1.1.5.2. Identification 32](#_Toc383288835)

[8.1.1.5.3. Blood Donation 32](#_Toc383288836)

[8.1.1.5.4. Treatment and Follow up of Volunteers 33](#_Toc383288837)

[8.1.1.5.5. Blood Analysis 34](#_Toc383288838)

[8.1.1.5.6. Duration of the Study Step A 35](#_Toc383288839)

[8.1.2. Step B - Sporozoite Challenge 36](#_Toc383288840)

[8.1.2.1. Selection of Volunteers 36](#_Toc383288841)

[8.1.2.1.1. Naïve Volunteers 36](#_Toc383288842)

[8.1.2.1.2. Pre-immune Volunteers 36](#_Toc383288843)

[8.1.2.2. Sample size 37](#_Toc383288844)

[8.1.2.3. Inclusion Criteria for Step B Volunteers 37](#_Toc383288845)

[8.1.2.3.1. Inclusion Criteria for Naïve Volunteers 37](#_Toc383288846)

[8.1.2.3.2. Inclusion Criteria for Pre-immune Volunteers 38](#_Toc383288847)

[8.1.2.4. Exclusion Criteria for Volunteers to be Challenged 38](#_Toc383288848)

[8.1.2.4.1. Exclusion Criteria for Naïve Volunteers 38](#_Toc383288849)

[8.1.2.4.2. Exclusion Criteria for Pre-immune Volunteers 41](#_Toc383288850)

[8.1.2.5 Selection 41](#_Toc383288851)

[8.1.2.6. Study Procedures 41](#_Toc383288852)

[8.1.2.6.1. Informed Consent 42](#_Toc383288853)

[8.1.2.6.2. Identification 42](#_Toc383288854)

[8.1.2.6.3. Initial Evaluation 42](#_Toc383288855)

[8.1.2.6.4 Serology and Immunology Tests 42](#_Toc383288856)

[8.1.2.6.5 Selection of *Anopheles albimanus* Mosquitoes for the Challenge 43](#_Toc383288857)

[8.1.2.6.6 Maintenance of Infected Mosquitoes 43](#_Toc383288858)

[8.1.2.6.7 Preparation for the Challenge 43](#_Toc383288859)

[8.1.2.6.8 Sporozoite Challenge 44](#_Toc383288860)

[8.1.2.6.9 Post-Challenge Medical Management and Follow up Visits 45](#_Toc383288861)

[8.1.2.6.10 Diagnosis 45](#_Toc383288862)

[8.1.2.6.10.1 TBS Diagnosis 45](#_Toc383288863)

[8.1.2.6.10.2 Xenodiagnostic and Assessment of Gametocyte Infectivity: 45](#_Toc383288864)

[8.1.2.6.11 Follow up 46](#_Toc383288865)

[8.1.2.6.12 Treatment 46](#_Toc383288866)

[8.1.2.6.12.1 Chloroquine Resistance 47](#_Toc383288867)

[8.1.2.6.13 Monitoring of Antimalarial Treatment Initiation 47](#_Toc383288868)

[8.1.2.6.14 Monitoring for Parasitemia Relapse and Recrudescence. 47](#_Toc383288869)

[8.1.2.6.15 Travel Restrictions. 48](#_Toc383288870)

[8.1.2.6.16 Duration of Step B Study*.* 48](#_Toc383288871)

[8.1.3 Sample Collection, Processing and Storage 49](#_Toc383288872)

[8.1.4 Data Management and Analysis 49](#_Toc383288873)

[8.1.5 Recordkeeping 49](#_Toc383288874)

[9. RISKS FOR VOLUNTEERS, RESEARCH GROUP AND ENVIRONMENT - PRECAUTIONS TO MINIMIZE RISK 50](#_Toc383288875)

[9.1 RISKS FOR VOLUNTEERS DONATING BLOOD. 51](#_Toc383288876)

[9.2 MALARIA CHALLENGE ASSOCIATED RISKS FOR THE VOLUNTEER 51](#_Toc383288877)

[9.3 ANTIMALARIAL TREATMENT ASSOCIATED RISKS FOR THE VOLUNTEERS 53](#_Toc383288878)

[9.4 RISK FOR THE STUDY STAFF 54](#_Toc383288879)

[9.5 RISKS AND PRECAUTIONS RELATED TO THE ENVIRONMENT 54](#_Toc383288880)

[10. BENEFITS 54](#_Toc383288881)

[10.1 BENEFITS FOR STEP A VOLUNTEERS 54](#_Toc383288882)

[10.2 BENEFITS FOR STEP B VOLUNTEERS 55](#_Toc383288883)

[11. COMPENSATION 55](#_Toc383288884)

[11.1 COMPENSATION FOR STEP A VOLUNTEERS 55](#_Toc383288885)

[11.2 COMPENSATION FOR STEP B VOLUNTEERS 55](#_Toc383288886)

[12. CRITERIA FOR WITHDRAWAL OF VOLUNTEERS FROM THE STUDY 55](#_Toc383288887)

[13. ADVERSE EVENTS (AE) 56](#_Toc383288888)

[13.1 SERIOUS ADVERSE EVENTS:(SAE) 61](#_Toc383288889)

[13.2 AE CLASSIFICATION – RELATION TO STUDY ACTIVITIES 61](#_Toc383288890)

[13.3 AE REPORT 62](#_Toc383288891)

[13.4 PERIOD OF AE MONITORING 62](#_Toc383288892)

[14. ETHICAL CONSIDERATIONS 63](#_Toc383288893)

[14.1 APPROVAL BY THE ETHICS COMMITTEES AND ORGANIZATION PLAN 63](#_Toc383288894)

[14.2 AFFILIATION OF THE ETHICS COMMITTEES TO THE UNITED STATES FEDERAL WIDE ASSRANCE (FWA) 63](#_Toc383288895)

[15. RESEARCH RELATED INJURY 63](#_Toc383288896)

[16. GOOD CLINICAL PRACTICES (GCPs) AND GOOD LABORATORY PRACTICES (GLPs) IN MVDC AND ASOCLINIC 64](#_Toc383288897)

[17. CONFIDENTIALITY 64](#_Toc383288898)

[18. RULES FOR THE INTERRUPTION OF STUDY. 64](#_Toc383288899)

[19. USE OF INFORMATION AND PUBLICATION OF STUDY RESULTS 65](#_Toc383288900)

[20. PROTOCOL DEVIATIONS AND AMENDMENTS 65](#_Toc383288901)

[21. WITHDRAWL OF VOLUNTEERS FROM THE STUDY. 65](#_Toc383288902)

[21.1 MONITORING OF VOLUNTEER WHO DO NOT CONTINUE IN THE STUDY. 65](#_Toc383288903)

[22. FUNDING. 66](#_Toc383288904)

[23. SCIENTIFIC AGREEMENTS 66](#_Toc383288905)

[24. REFERENCES 67](#_Toc383288906)

[ANNEX 1: STUDY PROCEDURES 76](#_Toc383288907)

[Step A Procedures 76](#_Toc383288908)

[Step B Procedures 77](#_Toc383288909)

3. LIST OF ABBREVIATIONS

| **ABBREVIATIONS** | **DEFINITIONS** |
| --- | --- |
| *An.* | *Anopheles* |
| CAB | Center of Applied Biotechnology (*Centro de Biotecnología Aplicada - CBA*) |
| AE | Adverse event |
| CRF | Case report form |
| Anti-HBc | Anti-hepatitis B core antibodies |
| Anti-HBsAg | Hepatitis B surface antigen |
| β-HCG | Beta-human chorionic gonadotropin |
| BUN | Blood urea nitrogen |
| CECIV | *Comité de Ética Centro Internacional de Vacunas* (Ethics Committee of MVDC |
| CRF | Case report form |
| CRUC | Clinical research unit coordination |
| DNA | Deoxyribonucleic acid |
| EKG | Electrocardiogram |
| ELISA | Enzyme-Linked Immunosorbent assay |
| EMVI | European Malaria Vaccine Initiative |
| EPS | Health Promoting Company (*Empresa Promotora de Salud EPS*) |
| ER | Emergency room |
| FDA | Food and Drug Administration |
| FUCEP | Primate Research Center Foundation (*Fundación Centro de Estudio de Primates*) |
| FWA | Federal-Wide Assurance |
| G6PD | Glucose 6 Phosphate Dehydrogenase |
| GCP | Good clinical practices |
| GLP | Good laboratory practices |
| HBV | Hepatitis B Virus |
| HCV | Hepatitis C Virus |
| HIV | Human Immunodeficiency virus |
| HTLV | Human T-lymphotropic virus |
| IC | Informed consent |
| IFAT | Indirect Immunofluorescence Assay Technique |
| INS | National Institute of Health of Colombia (*Instituto Nacional de Salud de Colombia*) |
| IPS | Health Care Provider Institution (*Institución Prestadora de Servicios de Salud IPS*) |
| IRB | Institutional Review Board - Ethics Committee |
| MFA | Membrane feeding assay |
| LDH | Lactate dehydrogenase |
| MVDC | Malaria Vaccine and Drug Development Center (*Centro Internacional de Vacunas CIV*) |
| NIAID | USA National Institute of Allergy and Infectious Diseases |
| NIH | A National Institute of Health |
| NHLBI | USA National Heart, Lung and Blood Institute |
| *P.* | *Plasmodium* |
| PI | Principal investigator |
| PvCS | *P. vivax* CS protein |
| PvMSP-1 | *P. vivax* Merozoite Surface Protein 1 |
| RBC | Red blood cell |
| RPR | Rapid plasma reagin |
| SAE | Serious adverse event |
| SIVIGILA | National System of Public Health Surveillance (*Sistema Nacional de Vigilancia en Salud Publica – SIVIGILA*) |
| SOP | Standard operating procedure |
| SP | Sulfadoxine pyrimethamine |
| spp*.* | Species |
| TBS | Thick blood smear |
| TDR | WHO Special Program for Research and Training in Tropical Diseases |
| TMRC | Tropical Medicine Research Center |
| WHO | World Health Organization |

4. BACKROUND AND RATIONALE

Malaria is the most important parasitic disease in humans. In 2004, 107 countries presented areas malaria transmission risk and an estimated of 3.2 billion people were living in those areas. Malaria is one of the three main causes of morbidity and mortality in developing countries, causing between 190 and 311 million clinical cases and more than 850.000 deaths per year (WHO, 2009). Nowadays multiple strategies are implemented to control malaria in endemic areas, all of them targeted to reduce mosquito populations or interrupt their contact with the human host; additionally, multiple anti-malarial drugs are used to avoid clinical manifestations of the infection, thus preventing risk of death, and are occasionally used as prophylactic therapy for people travelling to endemic areas. Although this control measures were initially very effective to handle the disease and eliminate malaria in many endemic regions, multiple factors have contributed to the development of mosquito resistance to insecticides and to *Plasmodium* spp. resistance to the therapeutic agents (Danis, 2003; Wilairatana *et al*., 2002; Talisuna *et al*.*,* 2004). During the last decades, parasite and mosquito resistance have increased and distributed throughout endemic areas, which stresses the importance of implementing other control measures including vaccines.

There are many studies that support the possibility of developing malaria vaccines that can effectively contribute to the control of this disease: first of all, in malaria endemic areas, in individuals that are continuously exposed to *Plasmodium* infection, there is progressive decrease in the intensity of the clinical manifestations until clinical immunity is achieved (McGregor *et al*., 1956); second, serum of individuals from endemic areas blocks parasite transmission to the mosquito (Hisaeda and Yasutomo, 2002); third, individuals and animals immunized with parasite sporozoites attenuated by irradiation can become immune to experimental challenge with viable sporozoites (Rieckmann, 1979; Clyde 1990; Hoffman *et al*.*,* 2002). During the last decade, there have been a number of clinical trials that show the feasibility of inducing protection against *P. falciparum* through immunization with RTS,S, a candidate vaccine formulation based on the circumsporozoite protein (PfCS) (Stoute *et al*.*,* 1997, Abdulla *et al*.*,* 2008 2008).

Natural transmission of malaria occurs through exposure of the host (human) to the bite of infected *Anopheles* mosquitoes. *Plasmodium* spp. sporozoites located in the salivary glands of mosquitoes are inoculated into the host’s peripheral circulation and travel through the blood stream to the liver. Sporozoites quickly invade hepatocytes and start asexual replication for a period of 2 to 10 days, during which every sporozoite produces an average of 30.000 uninuclear merozoites per infected hepatocyte (Meis *et al.*, 1990). During the hepatic stage, there is no evidence of clinical manifestations of the disease. Hepatic merozoites are subsequently released into the blood circulation and invade the red blood cells (RBCs), inside which they continue to multiply asexually, ultimately causing the rupture of the infected RBC. This stage of the infection is responsible for the clinical symptoms of malaria that present in a cyclic way, where the infected RBC containing mature schizonts ruptures every 48 hours, releasing 15 to 30 new merozoites that can adhere and penetrate other non-infected RBCs to restart the cycle. Later, some merozoites differentiate into micro and macro gametocytes that when ingested by a mosquito, exflagellate, fertilize and give rise the ookinete and later to the oocyst within which the sporozoites are formed. Sporozoites then migrate from the midgut to the salivary glands of the mosquito and are transmitted to the human host during a subsequent blood meal (Baton *et al*.*,* 2005).

Nowadays, there are approximately 190 to 331 million cases of clinical malaria, which results in close to one million deaths per year. It is estimated that in the Americas, 77% of clinical cases are caused by *P. vivax* (WHO, 2009). In 2001 it was estimated at *P. vivax* generated a burden of 100 million cases per year worldwide (Mendis *et al*., 2001). In many regions including Asia, Oceania and Latin America, the population is affected by either *P. vivax* or *P. falciparum*, and *P. vivax* is the predominant species in most regions. For this reason, *P. vivax* infection is still considered a thread that causes a significant impact in non-immune individuals, international tourists and national travelers. Although the disease caused by *P. vivax* is rarely lethal, it is clinically indistinguishable of severe malaria caused by *P. falciparum.* Also, *P. vivax* frequently produces hypnozoites in the liver, which can reactivate and emerge weeks or months later causing clinical relapses (Krotoski, 1989; Cogswell, 1992). Even though resistance to antimalarial drugs is way much lower in *P. vivax* than in *P. falciparum,* some parasite strains are less sensitive than others to conventional treatments; *P. vivax* resistance to chloroquine has been reported in Asia and South America and a lower response to primaquine has also been demonstrated in some *P. vivax* laboratory strains (Collins and Jeffrey, 1996; Duarte *et al*., 2001)

During the last two decades significant efforts have been made to develop a vaccine. Due to the increased presence of *P. falciparum* worldwide, with over 300 million clinical cases and the highest proportion of deaths per year (WHO, 2005), more efforts have been targeted to developing a vaccine to control this species, and multiple candidate vaccines against *P. falciparum* are being tested in clinical trials (Genton and Corradin, 2002, Ballou *et al*., 2004). Most of malaria vaccine candidates correspond to antigens from the pre-erythrocytic and erythrocytic stages of. *P. falciparum*

The availability of *P. falciparum* *in vitro* cultures have facilitated the development of reliable challenge models using sporozoites from genetically characterized parasites (*P. falciparum* NF-54 and 3D7 clone), allowing for the establishment of master reference cell banks in accordance with the standards of the Food and Drug Administration (FDA) of the United States. With this system of infectious challenge for *P. falciparum*, hundreds of volunteers were infected, in a safe and reproducible manner in 97% of cases, which have revealed key patterns for evaluating the protective efficacy of various pre-erythrocytic vaccine formulations against *P. falciparum* malaria (Hoffman, 1997). Additionally, taking advantage of the availability of this system, over the last two years significant efforts have been invested in developing a strategy based on the use of irradiation-attenuated *P. falciparum* sporozoites as vaccine (Luke and Hoffman, 2003).

As in most cases the mosquito vectors of *P. vivax* and *P. falciparum* are the same, mosquito resistance to insecticides increases the transmission of both parasites. In addition, the overall use of a mono-specific vaccine against malaria transmission in regions with more than one parasite species may raise ethical and epidemiological concerns. All these factors reinforce the need for a vaccine to prevent infection with both *P. falciparum* and *P. vivax*.

Considering the recent progress in the development of vaccines against *P. falciparum* and that some vaccine candidates against *P. vivax* that are being considered to be tested in clinical trials, it is critical to develop a reproducible challenge model to evaluate vaccines against *P. vivax* in phase ll clinical trials. Since the last decade, the Malaria Vaccine and Drug Development Center (MVDC) in Cali (Colombia) has devoted great efforts to the study of several preclinical vaccine candidates specific for *P. vivax* (Arévalo-Herrera *et al*. 2010). Among these studies, it has been of particular importance to determine the potential of the *P. vivax* CS protein (PvCS) as vaccine candidate (Herrera *et al.*, 2010).

Initially several groups conducted extensive immunological characterization of the PvCS protein led to the identification of multiple B, CD8+ T-cell and Th epitopes (George *et al*., 1990; Nardin *et al*., 1991; Herrera *et al*., 1992; Good *et al*., 1998; Arévalo-Herrera *et al*., 1998). Next, our group conducted preclinical studies in rodents and primates to study the toxicity and immunogenicity of various vaccine formulations designed based on previous studies (Herrera *et al*., 1994; Herrera *et al*., 1997). Trials were subsequently escalated in volunteers without malarial experience (Phase la trials), in which the safety, tolerability and immunogenicity of various vaccine formulations based long synthetic peptides containing multiple PvCS epitopes was evaluated. These studies have evaluated staggered doses of vaccines containing different regions of the protein (N, R, C fragments), individually formulated in Montanide ISA 720 adjuvant, and recently mixture of the three peptides (N, R, C) formulated both in Montanide ISA 720 adjuvant and in Montanide ISA 51 adjuvant. The two trials involving more than 100 naive volunteers showed good safety and tolerability, and a significant immunogenicity and reproducibility (Herrera *et al*., 2005, Herrera *et al.*, 2010).

Additionally, *P. vivax* vaccine candidates aimed at blocking parasite transmission to the mosquito (Pvs25) and to interrupt the erythrocytic development of the parasite (MSP-1/200L and DBP-RII) were evaluated in pre-clinical assays (Arévalo-Herrera *et al*., 2005a; Valderrama *et al*.,2005; Arévalo-Herrera *et al*., 2005c). It is expected that other candidates would be subjected to evaluation within the next few years (Chauhan and Bhardwaj, 2003; Genton and Corradin, 2002, Ballou *et al*., 2004). MVDC has initiated the preparation of other clinical trials, including Phase II Clinical Trials with both *P. vivax* CS, as well as with irradiated sporozoites, which require a reproducible sporozoite challenge system.

4.1. BACKGROUND OF EXPERIMENTAL CHALLENGE WITH MALARIA SPOROZOITES

4.1.1. Background of Experimental Infectious Challenges with *P. falciparum* sporozoites

The experimental infection of human volunteers with malaria parasites has been performed for > 100 years, when people were experimentally infected with *P. falciparum* to show that the parasite could be transmitted by mosquito bites (Grassi *et al*., 1899) and to understand the life cycle of malaria parasites in humans (Fairely, 1947). Similarly, routine infection with *P. vivax* was done as treatment for neurosyphilis (“malariotherapy”) during the 50’s-60’s (Glynn *et al*., 1994). In these studies, parasite blood was inoculated from patients with malaria to patients with syphilis to induce a high fever, to which it was attributed the destruction of *Treponema pallidum*, responsible for syphilis (Glynn *et al*., 1994; Glynn and Bradley, 1995). Later, a challenge model with *P. falciparum* and *P. vivax* sporozoites was developed and used to test the protective efficacy of sporozoites attenuated by radiation as vaccine (Egan *et al*., 1993; Rieckmann *et al*., 1979; Clyde *et al*., 1973, Clyde *et al*., 1975; Herrington *et al*., 1991).

During the last decade, the *P. falciparum* challenge model was widely used to determine the protective efficacy of several vaccines in in major research centers around the world: The Naval Medical Research Center (NMRC) in the United States (Rockville, MD) under Dr. S. Hoffman direction (Hoffman *et al*., 2002), the Center for Clinical Vaccinology and Tropical Medicine, Oxford University (Walther *et al*., 2005) and the Department of Medical Microbiology of the Radboud University Nijmegen Medical Centre (Hermensen *et al*., 2004). More recently, the group from Nijmegen conducted a study in which 15 healthy human volunteers (10 vaccinees and 5 controls) were vaccinated by exposure to bites of *P. falciparum*-infected mosquitoes once a month for 3 months during which they were under chloroquine prophylaxis. The volunteers were challenged by the bite of 5 infected mosquitoes, which confirmed the induction of protection (Roestenberg *et al*., 2009). Given the importance of infectious challenge models , in 2009 a consultative meeting was convened by funding agencies (PATH MVI, USAID, EMVI and WHO), in which scientists and regulatory and funding agencies discussed about the need to strengthen this kind of studies, particularly in endemic countries (Morthy *et al*., 2009).

In 1970, Powell and McNamara conducted an experimental challenge to evaluate the capacity of *P. falciparum* to induce infection in healthy volunteers exposed to 1–5 bites, compared to doses of >10 bites, which detected a significant difference in the average pre-patent period. After the establishment of the NF54 isolate of *P. falciparum* in continuous *in vitro* culture, this gametocyte-producing parasite has been widely used to infect *Anopheles stephensi* mosquitoes (Chulay *et al*., 1986).

In terms of the number of infective bites required to induce a reproducible infection, a study from Rieckmann (1990) demonstrated that malaria was transmitted to half of the volunteers after exposure to 1-2 bites of sporozoite-infected mosquitoes while Church *et al.* (1997) found that exposure to 1–5 *A. stephensi* or *A. freeborni* infected mosquitoes induced infections in all the exposed volunteers. Infectivity was not correlated with the number of sporozoites in the salivary glands of mosquitoes, but a negative correlation was observed between the duration of the pre-patent period and the number of infective mosquitoes.

In a review by Hoffman (1997), the results of a series of clinical trials in which about 200 volunteers were infected by exposure to bites of mosquitoes carrying *P. falciparum* sporozoites are summarized. From these experiments, a dose of 5 bites was adopted to induce a reproducible *P. falciparum* infection (Church *et al*., 1997). The level of parasitemia developed in these volunteers was low, 0.05% parasitemia were observed, corresponding to 100 times less parasitemia than the parasitemia presented in patients with severe malaria. Nearly all volunteers were symptomatic and recovered without sequels after full antimalarial treatment. In these studies, no relapses of infection were reported.

In brief, *P. falciparum* malaria induced in volunteers under highly controlled conditions and followed by complete treatment has had an excellent safety record, with no serious adverse events (SAE) occurring in the challenges made in hundreds of volunteers from the United States Navy.

These experiments have shown that the challenges in humans by exposure to infective mosquito bites under strict observation and control are possible, reliable and safe. The challenge model with *P. falciparum* sporozoites has been widely used in the development of antimalarial drugs and still plays a critical role in the evaluation of the efficacy of several vaccine candidates against this parasite species. These studies have been guided by important institutions of North America and Europe, engaged in research for vaccines against malaria (Ballou *et al*., 2004).

In contrast to *P. falciparum*, there is limited experience with the model of human infectious challenge with *P. vivax* sporozoites*.* During the 70s, this model was used to evaluate the protective efficacy of a vaccine of radiation-attenuated *P. vivax* sporozoites (Clyde, 1975; McCarthy and Clyde, 1977; Clyde, 1990). In these studies, in which a limited number of volunteers were exposed to the bites of infected *A. stephensi* mosquitoes (6 to 14 bites), it was observed that 2 of the volunteers exposed to 14 bites developed detectable parasitemia approximately 2 weeks after challenge (13 and 15 days, respectively), presenting a similar prepatency period with 6–7 bites. In a second clinical trial, 2 volunteers were exposed to a range of 8 to 12 bites. The prepatency period observed with the 8-bites regime (1 volunteer) was shorter (9 days) in comparison to the 14 days presented in the 12-bites regime (1 volunteer). Later, a third study was performed testing 6, 7 and 12 mosquito bites. In this study, it was observed that the prepatent period for 6-7 bites was 15 to 16 days, while for 12 bites it was 11 days. None of the above described studies provided information regarding relapses by *P.vivax* after the chloroquine/primaquine treatment.

4.1.2. Background of Experimental Infectious Challenges with *P. vivax* sporozoites

As *P.vivax* vaccine development progresses, particularly targeted to limiting the development of the pre-erythrocytic stages, the need for a safe and reproducible infectious challenge to test the protective efficacy of *P. vivax* vaccines increases (Moorthy *et al*., 2009)*.* During the last years, our group has made important progress in the development of vaccines against the *P. vivax,* and has dedicated particular efforts to the development of vaccines based on the PvCS protein, which has the potential of preventing the development of hepatic infection and consequently the subsequent phases of the parasite cycle.

Due to the lack of continuous *in vitro* cultures for *P. vivax*, we have standardized the procedure to infect *Anopheles albimanus* mosquitoes using wild parasites from infected patients (Salas *et al*., 1994). This procedure allows performing experimental infections regularly using the artificial membranes feeding assay (MFA) technique (Howard *et al*.1997). Infected mosquitoes obtained through this procedure have been used to optimize experimental infection with *P. vivax*, as well as the irradiation process and its use for immunization of primates (Jordan *et al*., 2006; Manzano *et al*., 2006). Research carried out by Manzano *et al.*, allowed to evaluate the model *P. vivax*–*An. albimanus* for its use in an experimental sporozoite infection in humans. A total of 138 blood samples collected from *P. vivax* infected donors, as confirmed by thick blood smear (TBS), were used to feed an equal number of mosquito batches and standardize mosquito infection predictable conditions to infect human volunteers. Using this system, our group has recently developed two clinical trials to determine the safety and reproducibility of infection with *P. vivax* sporozoites.

In the first trial, 17 out of a total of 18 human volunteers were successfully infected with *P. vivax* sporozoites administered through by the bites of 2-10 mosquitoes previously infected under controlled laboratory conditions. Except for one volunteer, all developed prepatent periods of 9-11 days, as determined by both TBS and PCR. All volunteers were treated immediately after diagnosed was confirmed, with a standard antimalarial therapeutic scheme and all responded quickly to treatment (Fernandez *et al*., 2005, Herrera *et al*., 2009). The safety of this assay was determined by closely monitoring the volunteers, according to good clinical practices (GCP) standards, in order to ensure early detection of adverse events (AEs). It was found that AE were mild, more frequently related to mosquito bites, and were usually resolved within 24 to 72 hours after challenge*.* During the paroxysm of the disease, 8 of the 17 volunteers displayed hematological abnormalities that normalized after completing the antimalarial treatment, as evidenced through safety assessments (Fernandez *et al*., 2005)

Subsequently, a second infectious challenge trial was conducted to verify the reproducibility of the model, in terms of safety and induction of patent infection, by using different *P. vivax* isolates obtained from acutely infected patients. Upon simultaneous infection of 3 groups of volunteers the study indicated that the inoculation of naive volunteers (n = 17) with doses of 2-4 infective bites completely reproduced the results obtained in the previous challenge (Herrera *et al*., 2010). In this second trial, the pre-patent periods maintained at an average of 11 days, and there were no AEs different from those expected from the transient *P.vivax* infection, which was rapidly cured after the administration of the standard antimalarial treatment (Herrera *et al*., 2010).

In the present infectious challenge trial, we aimed at to develop an additional model to evaluate the susceptibility to infection with viable *P. vivax* sporozoites in individuals that had been exposed to malaria in the past (pre-immune). This model would accelerate the future evaluation of multiple vaccine candidates and will provide continuity to the program for the development of a *P. vivax* vaccine, which has worldwide importance. This program has been supported over the last years by the Colombian Ministry of Health, WHO and NIH/NIAID.

The participation of individuals with previous *P. vivax* infection in this study, will contribute to the establishment of a less expensive system to assess the success of antimalarial vaccines in future Phase Ib and Phase IIb field trials. The possibility of reducing the number of individuals to dozens, e.g. 10-20 volunteers, will allow gathering preliminary information about the potential success of Phase IIb trials, which usually involved hundreds (300–500) of volunteers of endemic areas (UNDP/World Bank/TDR, 1997).

In this sense, this study will notably strengthen the research programs initiated by MVDC with the support of the WHO/TDR program (Contract: MVDC991006), and further supported by the WHO IVR program (Contract: LA-35735G). Moreover, it will contribute to accelerate the research program for the development of *P. vivax* vaccines currently sponsored by the Colombian government (Special Scientific Research #000253, 2005-2006). In this joined effort, USA National Institute of Health / National Institute of Allergies and Infectious Diseases (NIH/NIAID), has funded over the last decade a program initiated in 2002 for the establishment of a Tropical Medicine Research Center (Contract: AI49486-01), with a particular emphasis in the development of *P. vivax* vaccines*.* Additionally, this study will complement an ongoing trial sponsored by the National Heart, Lung and Blood Institute (contract: RHL086488A) for the establishment of a challenge model with radiation-attenuated *P. vivax* sporozoites. In that sense, this study will aim to produce immunological reagents (sera and cells) that provide information for the discovery of new antigens and genes through genomic and proteomic strategies.

Finally, this study has important significance as there is only a limited number of Centers around the world (2–3) suitable to develop this type of studies and only MVDC has reported challenge trials in recent years. Once the reproducibility of the model is confirmed, it will be available right away for Phase Ia/IIa and Phase Ib/IIb trials, for both pre-erythrocytic vaccines, for which PvCS is a suitable candidate in Phase II, as well as for other asexual blood stage candidates as PvMSP-1 (Pv200L) (Valderrama *et al*.*,* 2005) and la DBP (PvDBP-RII) (Arévalo-Herrera *et al*.*,* 2005a).

4.2. RATONALE FOR COMPARING THE *P. vivax* SPOROZOITE CHALLENGE IN NAIVE AND PRE-IMMUNE INDIVIDUALS

Each year, over 100 million cases of *P. vivax* malaria are reported around the world (Mendis, 2001; WHO, 2005). Efforts on vaccine development have mainly concentrated on *P. falciparum* malaria (Genton and Corradin, 2002; Ballou *et al*., 2004), in part due to availability of continuous *in vitro* cultures for this parasite species, which allows permanent access to parasite antigens for biochemical and immunological studies. These cultures have enabled also the development of more reliable challenge models with widely-characterized *P. falciparum* sporozoites, and have facilitated the establishment of master cell banks for *P. falciparum* under the FDA guidelines, which corresponds to the parasite clones of the NF-54 and 3D7 strains.

In contrast to *P. falciparum*, *P. vivax* can be only grown in culture for short periods of time, generally 4–6 days and for 1–2 cycles (Golenda *et al*., 1997; Chotivanich *et al*., 2001). This leaves laboratory work to depend on the continuous availability of parasites obtained from humans naturally infected in endemic areas with *P. vivax* malaria, or the use of non-human primates experimentally infected with *P. vivax* (Hurtado *et al*., 1997; Herrera *et al*., 2002).

Considering the progress described above in the development of *P. vivax* vaccine and that candidates such as PvCS have already began to be tested in clinical trials, it becomes critical to develop a challenge model with sporozoites to evaluate *P. vivax* vaccines in Phase IIa and IIb trials. In previous years, our group developed two Phase I trials that confirmed the safety, tolerability and high immunogenicity of PvCS. For this reason, MVDC is interested in moving forward to the Phase II assessment of this vaccine, during which its protective efficacy will be tested in an infective challenge with *P. vivax* sporozoites.

The proximity of the MVDC to areas where malaria is endemic, provides a unique opportunity to design Phase IIa and IIb trials in which the protective efficacy of a vaccine can be tested under controlled conditions individuals that have experienced *P. vivax* malaria during their life time. As described before, the lack of this model leads to the need to develop Phase IIb trials in a significant number of volunteers (hundreds) in endemic areas, which demands expensive installed logistic capacity. We consider that the development of this model will reduce the cost and time for the clinical evaluation of antimalarial vaccines in humans not only in Colombia but for research groups working on vaccine development.

4.3. RATIONALE FOR CONDUCTING THIS STUDY IN COLOMBIA

4.3.1. Epidemiology

*P. vivax* is the most frequently reported species causing malaria in the Americas, where it represents 77% of the cases (WHO 2009). By 2009, it was estimated that 18% of the Colombian population lived in areas where there are favorable conditions for the transmission of this disease (WHO, 2009).

Malaria transmission occurs in 9 countries of the Amazon Basin (Bolivia, Brazil, Colombia, Ecuador, French Guiana, Guyana, Peru, Suriname and Venezuela), 8 countries of Central America (Belize, Costa Rica, El Salvador, Guatemala, Honduras Nicaragua, Panama, Mexico) and two Caribbean countries (Haiti and Dominican Republic). Additionally, a small number of cases are reported from Argentina and Paraguay.

In Colombia, malaria cases have shown a significant increase over the past decades (Padilla, 1999). According to the WHO World Malaria Report FOR 2005, Colombia is among the Latin American countries with the highest incidence of malaria and higher proportion of *P. falciparum* cases (WHO, 2005). Approximately 65% of Colombian territory presents ecological conditions for malaria transmission; however, the fact that the majority of the population is concentrated in the mountains, prevents a more extensive transmission of this disease. About 40% of the Colombian population is concentrated in the main cities (Bogotá, Medellín and Cali), which are located in non-endemic areas. While this is a positive epidemiological trend, it also points out that malaria is highly concentrated in less developed populations.

Due to public order issues, policy interventions of the National Malaria Control Program are not in place in all regions and in 2003 more than 160,000 cases were reported, 58% of which corresponded to *P. vivax* malaria. The areas of greatest risk for transmission include the region of Bajo Cauca, the Pacific Coast and the upper region of Sinú river (WHO, 2005). The Pacific Coast, which stretches from the border with Panama to the border with Ecuador, consists of 4 departments which together contribute to approximately 30% of the total cases in the country. This proportion of cases is concentrated in geographic areas that represent only 3% of the Colombian population.

As previously mentioned, MVDC and Caucaseco represent a unique platform for the development and testing of vaccines against *P. vivax* malaria, given the long history of the groups, infrastructure and experience gained in recent years in clinical trials (Herrera 2005, Herrera 2009, Herrera 2011). MVDC is the leading group in this type of trials around the world (Herrera *et al*., 2009, Herrera *et al*., 2010 and Moorthy, V.S. *et al*., 2009).

4.3.2. Morbidity and Mortality of *P. vivax* infection in Colombia

According to data from SIVIGILA (epidemiology database of the Colombian National Institute of Health), 79,909 malaria cases were reported in 2009, 72% of which were infections caused by *P. vivax* and 27.1% by *P. falciparum*, for a 2.7:1 proportion of *P. vivax*:*P. falciparum* cases (Chaparro, 2009).

Until October 29, 2010, there were 101,360 new cases of malaria reported in the Colombia, with a predominance of *P. vivax* infections in 69.8% of cases, while *P. falciparum* caused 28.9% of infections (SIVIGILA, 2010).


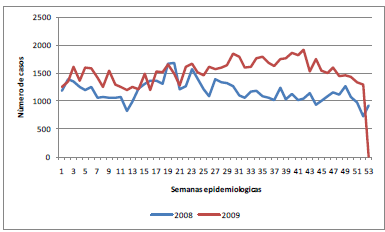


**Figure 1**. Malaria behavior in Colombia for 2008-2009.

Source: MALARIA BEHAVIOR IN COLOMBIA ACCORDING TO CASES REPORTED BY SIVIGILA IN 2009.


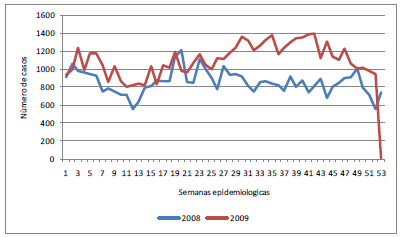


**Figure 2**. Behavior of *P vivax* malaria cases in Colombia for 2008-2009.

Source: MALARIA BEHAVIOR IN COLOMBIA ACCORDING TO CASES REPORTED BY SIVIGILA IN 2009.

4.4. BACKGROUND OF THE SCIENTIFIC GROUP AND FACILITIES OF THE RESEARCH SITES

4.4.1. Malaria Vaccine and Drug Development Center (MVDC)

MVDC is the result of over 25 years of scientific research. In 1985 a multidisciplinary research group for the study of malaria was established at the Department of Microbiology, Faculty of Health, Universidad del Valle in Cali, under the direction of Dr. Socrates Herrera, MD. Ten years later, research areas were established in the Colombian Pacific Coast, along with a center for primate studies, two insectaries, laboratories of applied and basic immunology*.* This allowed us to reproduce the full cycle of *P. falciparum* and *P. vivax* parasites and establish experimental models for the study of vaccines and antimalarials*.* In 1999, a clinical trial unit and the Malaria Vaccine and Drug Development Center (MVDC named in Spanish *Centro Internacional de Vacunas*) with the support of the WHO/TDR program, and which is linked to *Clínica Valle de Lili*, *Hospital Universitario del Valle* (HUV) and *Centro Médico Imbanaco*.

In 2000 the MVDC was funded by the NIH/NIAID to establish a Tropical Medicine Research center (TMRC), who developed the Translational Research in *P. vivax* protective immune mechanisms to vaccine development program, along with the NIAID Malaria Vaccine Design Unit (MVDU; Rockville,MD), the USA NAVY Medical Research Center (Silver Spring, MD) and other institutions around the world. Thanks to this program, MVDC has strengthened its relationship with institutes in the United States, such as NIAID/NIH MVDU, Navy Medical Research Center, Johns Hopkins Bloomberg School of Public Health, Tulane University, University of Miami, Fraunhofer IBMT Technology Center Hialeah (FTeCH), among others, and cooperation activities with the private industry such as: F Hoffmann -La Roche (Basel, Switzerland), Pablo Cassara Laboratories (Buenos Aires, Argentina) and other institutes in Europe, Latin America and Asia.

Our group has established itself as a worldwide leader in the development of a vaccine against *P. vivax*. Leadership recognized by the international scientific community through a significant number of scientific publications (Am J Trop Med Hyg 73 Vol*.* Suppl November 5, 2005), including studies conducted during the last decade to evaluate PvCS as a potential vaccine candidate in both preclinical and clinical trials (Arévalo-Herrera and Herrera, 2001). This has been achieved thanks to detailed immunological characterization studies developed by us and by other research groups around the world (Good *et al*., 1998; George *et al*., 1990; Arévalo-Herrera, 1998; Nardin *et al.*, 1991). In addition to clinical trials in rodents and primates (Herrera *et al*., 1994, 1997), the group has completed a Phase I clinical trial where the safety and immunogenicity of synthetic long peptides derived from PvCS formulated in Montanide ISA-720 adjuvant (Herrera *et al*., 2005) and one where the safety and immunogenicity of the mixture of three CS peptides was evaluated formulated in Montanide ISA-720 and Montanide ISA-51 adjuvants (Herrera *et al.*, 2010b). The planning and conduct of this research has been extensively consulted with the international scientific community and multiple ethics committees, as well as a continuous training of clinical researchers both in Colombia and abroad*.* During this process, MVDC has established Good Clinical Practices (GCP) and Good Laboratory Practices (GLP) standards. The group currently prepares for protective efficacy testing of CS in Phase II studies*.*

4.4.2. Caucaseco Scientific Research Center (Caucaseco)

Caucaseco was formally instituted in 2005 in response to the need of generating an appropriate scenario for biomedical scientific research in the South West region of Colombia. Since then, it has been working on establishing infrastructure and administrative capacities. Caucaseco Scientific Research Center currently hosts in their facilities the immunology laboratory **Asoclinic**, **MVDC**, Primate Research Center Foundation (**FUCEP**) and the Center of Applied Biotechnology (**CAB**). Recently, Caucaseco led a proposal for the establishment of an International Center of Excellence for Malaria Research (ICEMR) in response to an international call launched in 2009 by the NIAID/NIH.

Under this call, Caucaseco proposed the establishment of the Latin American Center for Malaria Research (*Centro Latinoamericano de Investigación en Malaria –* **CLAIM**), whose mission is to develop epidemiological research to generate the necessary information and malaria control strategies to support the governments of Colombia, Peru, Panama and Guatemala in their efforts to eliminate malaria. CLAIM is a new initiative of great value for characterizing endemic areas for the potential assessment of malaria vaccines in Phase IIb and Phase III trials. Caucaseco will provide all administrative and logistical support for the development of this study.

4.4.3. Center of Applied Biotechnology (CAB)

The Center of Applied Biotechnology (CAB) is a private institution created to implement the biotechnological processes in the production of reagents for scientific importance for both scientific research and for therapeutic and human diagnostic using animal species; additionally, the analysis of active ingredients with nutritional, pharmaceutical and industrial value in plant products.

Among the CAB units is the Entomology Unit, which houses colonies of *An. albimanus* mosquitoes originally established at between *Instituto de Inmunología del Valle* (IDIV) and MVDC. This colony of *Anopheles* has been used intensively for malaria research (Arevalo-Herrera *et al*., 2005). In recent years, this colony has facilitated the implementation of two infectious challenge trials in naive volunteers with *P. vivax* sporozoites (Herrera *et al*., 2009; Herrera *et al*., 2010). Additionally, this colony carries out studies for the identification of candidate vaccines designed to prevent malaria transmission from human to mosquitoes (TB vaccines) (Arevalo-Herrera *et al*., 2005).

5. PRELIMINARY DATA FROM THE MVDC ENTOMOLOGY UNIT

The Entomology unit has established colonies of *An. albimanus* mosquitoes in Cali (non-endemic area) and Buenaventura (endemic region for malaria), and in recent years has developed studies to improve the production of *P. vivax* sporozoites in both colonies. Both the insectary in Cali, as well as the one in Buenaventura will be used for the production of sporozoites for this study. The entomology group has established a standardized system to reproduce infections in *An. albimanus* mosquitoes in the laboratory and for the regular production system of *P. falciparum* and *P. vivax* sporozoites (Hurtado *et al*., 1997), and together with FUCEP have managed to produce infection of naïve monkeys (Herrera *et al*., 2002; Zapata *et al*., 2002: Perlaza *et al*., 2003). During the last 3 years conditions for breeding *An. albimanus* mosquitoes have been optimized, currently reaching a daily average production of 6,000 females. In addition, mosquito infections with *P. vivax*-infected blood collected from human volunteers have been optimized under ISO international quality standards. This has allowed conducting transmissions blocking studies in humans and monkeys (Arevalo-Herrera *et al*., 2005b,c) and to maintain a continuous monthly sporozoite production of about 8 million sporozoites. Such infrastructure proved its robustness in the two previous challenge trials, in which it was possible to establish conditions to predict the effectiveness of artificial membrane feeding procedures and production of viable sporozoites for this study (Herrera *et al*., 2009 sporozoites; Herrera *et al*., 2011).

6. HYPOTHESIS

Individuals without previous experience with malaria develop shorter prepatent periods than individuals who have previously had the infection (pre-immune).

7. STUDY OBJECTIVES

**7.1. PRIMARY OBJECTIVE**

Compare the susceptibility of naive and pre-immune volunteers to infectious challenge with viable *P. vivax* sporozoites.

7.2. SECONDARY OBJECTIVES

• Establish an experimental infection model using viable *P. vivax* sporozoites in individuals previously exposed to malaria in endemic areas.

• Evaluate and compare the pre-patent period induced in naive and pre-immune volunteers.

• Determine the clinical characteristics of the disease in the two groups of volunteers.

• Compare the immune response of volunteers after experimental exposure to the parasite.

8. STUDY DESIGN

The study is an open-label, randomized clinical trial designed to compare the susceptibility of naive and pre-immune volunteers to infectious challenge with viable *P. vivax* sporozoites. This comparison will facilitate the evaluation of the *P. vivax* vaccine efficacy in Phase IIb clinical trials. Specifically, the study will investigate if infection of naive and pre-immune volunteers through the bites of 3 ± 1 infected mosquitoes is reliable, reproducible and shows difference in the prepatent period. Groups of volunteers will be exposed to infection with mosquitoes infected with the same parasite strain.

**Step A - Blood donors infected with *P. vivax*:** Volunteers will be recruited passively from the group of patients attending to the MVDC malaria diagnosis laboratories (Pacific office) in Buenaventura, with active *P. vivax* infection, as determined by TBS. Initially, volunteers will be sufficiently informed about the study, in order to decide freely whether to participate in the study, in which case the participation will be formalized by signing the IC form. Once this process is completed, 35 mL of whole blood will be obtained from the volunteer by venipuncture. Blood will be distributed as follows: **5 mL** to be sent for infectious disease screening and **30 mL** to feed *An. albimanus* mosquitoes colonized in the CBA insectary in Cali and*/*or Buenaventura.

To obtain a batch of mosquitoes with appropriate characteristics for the experimental challenge, 5 to 15 lots of mosquitoes infected from the blood of the parasite donor patient will be examined to ensure that the lot for the challenge has the highest infection rate and is suitable to be used on the date scheduled for the challenge.

**Step B - Sporozoite Challenge:** The aim of this step is to determine whether exposure to 3 ± 1 bites of *P. vivax*-infected mosquitoes produces reproducible infections in human volunteers and reproduces the results obtained in previous challenge studies with *P. vivax* in human volunteers (Herrera *et al*., 2009, Herrera *et al*. 2011). Additionally, to identify whether there is any difference between naive and pre-immune volunteers in regards to parasitemia developing, specifically to the length of the prepatent period and*/*or clinical manifestations.

This step will involve a total of nineteen (19) volunteers plus four to six (4–6) alternative volunteers recruited in Cali and Buenaventura and that satisfy the inclusion criteria and who have accepted to participate in the study by signing freely and voluntarily the informed consent (IC) form*.* The 19 volunteers will be placed in two (2) groups: one of seven (7) naive volunteers and another one of 12 pre-immune volunteers. Each group will be exposed to 3 ± 1 bites of infected mosquitoes, as described below. A maximum of three (3) challenge exposure sessions per participant will be used to achieve this number of infective bites. From then on, volunteers will be clinically followed by the medical personnel assigned to the clinical trial and will be treated as soon as infection is detected in blood by TBS. The comparison of the characteristics of the infection induced in the two groups of volunteers will help determine the optimal conditions to test *P. vivax* vaccines in Phase IIa and IIb studies.

8.1. CLINICAL TRIAL PLAN

8.1.1. Step A: Donation of *P. vivax*-infected Blood

8.1.1.1. Selection of Volunteers

Volunteer parasite-donors will be recruited from patients attending MVDC in Cali or Buenaventura (MVDC Pacific Office), which are both certified by the Colombian government for malaria diagnosis. A bacteriologist or certified microscopists will perform the TBS diagnosis. If the result is positive for *P. vivax* and negative for other concomitant *Plasmodium* species the result will be informed to the patient. If parasitemia is ≥ 0.1%, the volunteer will be explained about the study purposes and procedures and will be invited to participate. If interested in participating, volunteers enrollment will be formalized by the voluntary and free signing of the IC form. The volunteer should sign freely and voluntarily two informed consents: the first is for the selection and participation in the study and the second for HIV testing. If the patient is a minor (15-17 years) the information will be explained to both, the minor and the parent or legal guardian. If both agree to participate, the minor should agree to sign an informed assent and the IC must be signed by a parent or legal guardian. A copy of the signed documents will be provided the volunteer at the end of the IC process.

8.1.1.2. Sample Size

A minimum of 5 and a maximum of 15 volunteers will be included for Step A. Samples of the volunteers will be evaluated to determine if they meet the inclusion criteria. Once having identified the samples of volunteers that are suitable for the challenge, the mosquito lot with higher infectivity will be chosen.

8.1.1.3. Inclusion Criteria

• Men or non-pregnant women >15 and <60 years of age.

• Not diagnosed with chronic diseases or acute diseases other than malaria diagnosed, whether infectious or not, that compromise any of the following systems: respiratory, cardiovascular, gastrointestinal, hepatic, renal, neurological, musculoskeletal, genitourinary, hematopoietic or psychiatric diseases diagnosed by a specialist.

• Diagnosis of *P. vivax* malaria by TBS.

- Negative diagnosis for *P. falciparum* and *P. malariae* malaria by either TBS or PCR.

• Parasitemia of ≥ 0.1%, as determined by TBS.

• Not having received anti-malarial treatment before TBS diagnosis.

• Be able to provide freely and voluntary IC, in the presence of two witnesses who should also sign the IC form. Volunteers aged between 15 and 17 years old will sign an informed assent and a parent or legal guardian should sign the informed consent.

8.1.1.4. Exclusion Criteria

- To be under 15 and over 60 years of age.

Justification: According to Colombian legislation people <14 are unable to provide IC. The age of 60 was chosen as the limit to avoid exposure of older people who may have additional health problems and be adversely affected by blood donation.

• Have or have had a disease or clinical condition, which at the criteria of the medical researchers increases the risk associated with participation in this study.

Justification: If necessary volunteers will be excluded to ensure the safety of the procedure and ensure a consistent blood supply.

• Having received transfusion of any blood component within the last 6 (six) months prior to the study.

Justification: A blood transfusion may be a risk factor for infectious diseases, which could be in window period at the time of transfusion.

• To have a Hb level of < 9 g/dL.

Justification: We have determined not include patients with low hemoglobin levels to avoid exposing them to some mild and transient hemodynamic changes that could occur by donation.

- To be participating as volunteer in other research studies.

8.1.1.5. Study Procedures

8.1.1.5.1. Informed Consent

People who come to MVDC headquarters in Cali or its Pacific Office in Buenaventura with a positive TBS for *P. vivax* will be informed of the study objectives, procedures, risks and benefits by one the study researchers. Then, to ensure full understanding of the study, the volunteer will be invited to ask questions to the researcher or may be asked additional questions by the study researcher. This process will be formalized by signing the IC form. If the volunteer is under 18 years of age, the explanation of the study will be given in the presence of at least one of parents or the legal guardian, the child will be asked to sign an informed assent and the informed consent will be sign by the parent or legal guardian. The IC forms and Case Report Forms (CRFs) will be documented in the folder of the participant. The volunteer is also asked to sign an additional IC form for HIV testing. For this, the volunteer is previously informed about the purpose of the test, how results will be informed and that initial counseling would be provided once the results are informed, if necessary. All volunteers will receive a copy of the IC form, which they have signed. If a potential volunteer cannot read and is interested in participating, a member of the research team will read the IC form to the volunteer, the volunteer will then explain it in its own words in the presence of two witnesses chosen by the volunteer and that are able to read and write. If the potential volunteer agrees to participate, he/she may place the right index fingerprint and the two appointed witnesses will sign the IC form.

.

8.1.1.5.2. Identification

Two identification codes will be established for Step A. The first one will be assigned for the laboratory screening tests (Screening code) and the second one for the MFA procedure (MFA code). The screening code will consist of three digits, the first one will correspond to the study Id number and the next two will be assigned consecutively according to the order of volunteer recruitment (01, 02, etc). The MFA code will consist of the consecutive code assigned by the Entomological Unit, preceded by the letters: BPV or HPV, where B means that the infection assay was done in Buenaventura and H in Cali (Hormiguero); P to the type of donor, which in this case is a person, and V to the type of malaria infection, i.e. vivax. The consecutive code of the MFA code is assigned according to the infection records of the Entomological Unit, which have been keep for the last 5 years. The coding procedure described above will be done in accordance to the ISO international standard guidelines for quality assurance.

8.1.1.5.3. Blood Donation

Once the volunteer has signed the IC and has been assigned a screening code, one of the doctors in the research team will gather the clinical history and performed a physical exam. This procedure will produce two possible outcomes:

• The volunteer does not fulfill the inclusion criteria: In this case, drug treatment for malaria will be provided according to the Colombian guidelines for treatment of malaria. The volunteer will be explained the reasons why he will not continue to participate in the study and no blood will be collected. Even though the volunteer is excluded from the study, he/she will be asked to come back two weeks later (on day 15 after initiating malaria treatment) for TBS examination to ensure complete cure. If the TBS is positive at day 15, drug treatment will be repeated. Cases of chloroquine resistance will be handled as described in the section: Treatment and Follow up of Volunteers (see below).

• The volunteer fulfils the inclusion criteria: In this case, 35 mL of blood will be collected by venipuncture, of which 0.5 mL will be used for infectious diseases screening and the other 30 mL will be used for MFA at the Entomological Unit. Upon completing the blood donation, volunteers will receive antimalarial drug treatment, according to recommendations of the Colombian Ministry of Health.

Volunteers will be asked to come back approximately one week later to receive the results of the screening tests. In the case of a positive result for any of the blood screening tests, included HIV, the volunteer will be provided with a copy of the test results and referred for counseling and medical assistance to the health care provider institution (denoted in Colombia as *Institución Prestadora de Servicios de Salud - IPS*) that corresponds to the healthcare regimen the volunteer is subscribed to. If the volunteer has a private physician, he will be referred with a copy of all his laboratory results, and if he is not subscribed to any healthcare regimen, he will be referred to one of the hospitals of the public health network ascribed to the Department’s Health Secretariat*.*

8.1.1.5.4. Treatment and Follow up of Volunteers

• Chloroquine: 600 mg (4 tablets, 150 mg each) on the first day, 450 mg (3 tablets) on the second day and 450 mg (3 tablets) on the third day.

• Primaquine: 15 mg (1 tablet) once a day for 14 days.

• Follow up: Volunteers will be asked to come back two weeks after initiating the drug treatment (on day 15 after the first dose) for TBS examination in order to ensure complete cure. Drug treatment will be repeated if TBS is positive.

Cases of chloroquine resistance (*) be handled with a combination of sulfadoxine/pyrimethamine (Falcidar®: 25 mg pyrimethamine/ 500 mg sulfadoxine), as alternative treatment, administered in 3 tablets on a single dose.

If the patient has a condition that contraindicates the administration of Falcidar® (e.g. allergy to sulfa drugs), he will be administered 3 tables of amodiaquine (200 mg per tablet, 600 mg/day) for 3 days (Bosman, *et al.*, 2001) and will be asked to come back one week later for TBS testing to confirm complete cure.

(*) In case chloroquine resistance is confirmed, the lot of mosquitoes fed on that blood sample will be discarded. If such lot was used for the challenge, the drug treatment of Step B volunteers will be modified accordingly.

8.1.1.5.5. Blood Analysis

**Plasmodium PCR**: A 500 μL blood sample will be used to test for *P. vivax*, *P. falciparum* and *P. malariae* by PCR in order to rule out mixed infections.

PCR testing has specificity and sensitivity values higher than any other diagnostic test currently available (WHO, 2000) and therefore is considered the “Gold Standard” for malaria diagnosis in research studies. The test reaches sensitivities and specificities of 100% compared to other diagnostic methods available (Pöschl B. *et al*., 2009).

In the clinical scenario, however, PCR is not the chosen diagnostic method because it requires of specialized equipment and expensive reagents that are not available at many places. Moreover, since test results are not readily available, this will delay initiation of treatment for the patient. This will not be a difficulty for this study since TBS will be used for diagnosis, therefore ensuring that adequate treatment is initiated right away.

PCR testing will be performed on blood collected from the Step A volunteer to rule out infection by other *Plasmodium* species, after using this blood to fed mosquitoes, but before using this lot to challenge healthy volunteers in Step B. In case a sample is positive by PCR for a *Plasmodium* species other than *P. vivax*, the lot of mosquitoes fed on this blood will be discarded according to the corresponding biosafety guidelines and will not be used to challenge healthy volunteers in Step B.

Even though the possibility of a false negative PCR result for *P. falciparum* is very low, if there is a mixed malarial infection that was not diagnosed by PCR, this will be detected during the post-treatment follow up since the symptoms will not improve with the drug treatment provided for *P. vivax*, as it is not effective against *P. falciparum*. At this time, TBS and PCR testing will be performed and adequate treatment for *P. falciparum* will be provided. Mosquitoes fed on such blood will be discarded.

**Blood bank screening tests:** A 5-mL blood sample will be used to perform the following tests: two rapid HIV diagnostic tests from different manufacturers, antibodies against HTLV 1 and 2, Hepatitis B surface antigen (HBsAg), rapid diagnostic test for Hepatitis C, rapid diagnostic test for Chagas and Rapid Plasma Reagin (RPR) for syphilis.

**Confirmatory tests:** If any of the rapid diagnostic tests for HIV is positive, samples will be tested by Western blot to confirm diagnosis. Samples positive for HBsAg will be tested for antibodies to HBV core antigen for confirmation. Similarly, if the RPR tests is positive at any dilution, the sample will be tested by Fluorescent Treponemal Antibody-Absorption (FTA-ABS) test.

Although it is theoretically possible for mosquitoes to transmit Hepatitis B virus within the 72 hours after taking a blood meal (Blow *et al*., 2002), there is currently no evidence that any of these diseases is transmitted by *Anopheles* mosquitoes. Furthermore, mosquitoes will be reared for 15–18 days prior to the challenge, period after which transmission of any of these diseases is highly unlikely.

**Additional analyses:** In addition to the routine blood bank screening tests, other pathogens that may be inadvertently transmitted by *Anopheles* mosquitoes during the parasite challenge are considered.

Below is a description of diseases reported in Colombia that could be potentially transmitted by *Anopheles* mosquitoes and a description of discussions held about each one before conducting the first challenge trials (Herrera *et al*., 2009) with experts in vector-borne transmitted diseases.

**Filariae:** Dr. Augusto Corredor, ex-director of the Parasitology Unit of the Colombian Institute of Health, and Dr. David Botero (parasitologist), professor of Universidad de Antioquia (Medellín, Colombia) confirmed that the is no evidence of transmission for any filarial species in Colombia and that screening for this parasites is not necessary. These opinions where confirmed at that time by Dr. Duane J. Gubler, Division of Vector-Borne Infectious Diseases, USA National Center for Infectious Diseases, Centers for Disease Control and Prevention (CDC).

**Other parasites:** Although *Leishmania* spp., are endemic in this region, they are not transmitted by *Anopheles* mosquitoes.

**Viruses:** Same as described above for parasitic disease, before conducting the first challenge trial, Dr. Jorge Boshell (virologist), ex-director of the Colombian National Institute of Health, and Dr. Robert B. Tesh (MD), professor of the Department of Parasitology at the Center for Tropical Diseases of the University of Texas (Galveston, Texas), were consulted to determine the possible need of testing blood donations for viruses different from the ones tested for by the blood blank screening tests. Both consultants considered it unnecessary.

Additionally, volunteers of the two previous challenge trials followed up for 1-3 years (Herrera *et al.*, 2009; Herrera *et al.*, 2011), have not referred any pathology that could be associated to the experimental challenge.

8.1.1.5.6. Duration of the Study Step A

Step A is scheduled to last two (2) months. During the first month, the community of the endemic area will be informed about the study through posters, flyers, press releases and group talks. These talks will be held twice per week and will explain the study objectives, procedures, risks and benefits, while the other types of the information will be provided continually. During this time, volunteer recruitment activities will be conducted at the MVDC office in Buenaventura (INSALPA), which will consist of explaining the study and performing the inform consent process to patients that are diagnosed with *P. vivax* malaria by TBS and collecting blood samples (35 mL) from patients that agree to participate in the study by signing the IC. After completing the blood donation process, drug treatment will be provided according to the guidelines of the Colombian Ministry of Health. These blood samples will be screened for infectious diseases (HIV, Chagas, HTLV 1-2, syphilis, Hepatitis B, Hepatitis C and malaria other than *P. vivax* malaria), and used for MFA. If the sample is negative for these infectious diseases, the lot of mosquitoes fed on such blood sample will be considered optimal for challenging healthy volunteers in the study Step B. Volunteers will be followed up by phone every other day until day 7 after the initiation of the antimalarial treatment.

Mosquitoes fed on *P. vivax*-infected blood will be kept at the Entomological Unit until used.

8.1.2. Step B - Sporozoite Challenge

8.1.2.1. Selection of Volunteers

Volunteers for this step of the study will be from Cali, a non-endemic region for malaria, and from Buenaventura, a malaria-endemic region. Volunteers will be invited to participate through different activities such as conferences, group meeting and other media, previously approved by the IRB, such as posters and slide presentations. Volunteers will be selected based on the study inclusion criteria. Volunteers for the Step B will be included in the study before initiating inclusion of volunteers for Step A.

8.1.2.1.1. Naïve Volunteers

Naive volunteers will be sought for among people from Cali that have no history of malaria infection. Volunteers that are willing to participate in the study will be screened to determine the presence and titers of antibodies against *P. vivax* parasites and against specific parasite proteins such as PvCS and Pv200L, by IFAT and ELISA, respectively. Such volunteers should also fulfill the inclusion criteria.

8.1.2.1.2. Pre-immune Volunteers

Pre-immune volunteers will be recruited in Buenaventura a malaria endemic area, among individuals with clinical history of *P. vivax* malaria. Volunteers willing to participate in the study will be screened to determine the presence of antibodies to whole *P. vivax* parasites by IFAT and/or against specific parasite proteins such as PvCS and Pv200L, by ELISA,. Such volunteers should also fulfil the inclusion criteria.

8.1.2.2. Sample size

This study proposes to compare the susceptibility of naïve and pre-immune volunteers to *P. vivax* malaria. A total of 19 volunteers (plus 4-6 optional volunteers) will be recruited in Cali and Buenaventura. This number will guarantee having a group of 7 naïve volunteers and 12 pre-immune volunteers, so that each group is challenged with the same parasite isolate through an infection scheme that uses in average three infective mosquito bites. The sample size, seven (7) naïve volunteers and twelve (12) pre-immune volunteers, is adequate to test the study hypothesis and conduct reliable challenge trials in the future, without exposing too many volunteers in the present study to unnecessary risks.

8.1.2.3. Inclusion Criteria for Step B Volunteers

8.1.2.3.1. Inclusion Criteria for Naïve Volunteers

- Healthy men or non-pregnant women within 18–45 years of age.
- To have completed the IC process freely and voluntarily in the presence of two witnesses, who will also sign the IC form.
- To show sufficient understanding of the clinical trial, as evidenced by correct answer of a questionnaire.
- No history of malaria infection.
- To have no history of chronic or acute diseases, whether infectious or not, that affect one of the following systems: respiratory, cardiovascular, gastrointestinal, hepatic, renal, neurological, musculoskeletal, genitourinary, hematopoietic or psychiatric diseases diagnosed by a specialist.
- Woman participants must agree to use adequate contraception starting a month before the challenge until three months after the study completion.
- Accepting not to travel outside the study area from the seventh (7^th^) to the thirty first (31^st^) day post-challenge, which is the period at which there is higher risk to develop parasitemia.
- Accepting not to travel to areas considered endemic for malaria during the study period (Buenaventura, Tierralta, Quibdó, Tumaco, Urabá and Bajo Cauca).
- To be reachable by phone during the entire study period.
- To be Duffy positive.
- To have a Hb >11 g/dL.
- To be available to participate during the period in which the study is scheduled.

8.1.2.3.2. Inclusion Criteria for Pre-immune Volunteers

- Healthy men and non-pregnant women within 18–45 years of age.
- To complete the IC process freely and voluntarily in the company of two witnesses, who also signed the IC form.
- To have a history of *P. vivax* infection within the last 12 months, as determined by medical history and serological tests.
- To have no history of chronic or acute diseases, whether infectious or not, that affect one of the following systems: respiratory, cardiovascular, gastrointestinal, hepatic, renal, neurological, musculoskeletal, genitourinary, hematopoietic or psychiatric diseases diagnosed by a specialist.
- Volunteer women must agree to use adequate contraception starting a month before the challenge until three months after the challenge.
- Accepting not to travel outside the study area from day seven (7) to day 31 post-challenge (the period of highest risk of developing parasitemia).
- Accepting not to travel to areas considered endemic for malaria during the study period (Buenaventura, Tierralta, Quibdó, Tumaco, Urabá and Bajo Cauca).
- To be reachable by phone during the entire study period.
- To be available to participate during the period in which the study is scheduled.

8.1.2.4. Exclusion Criteria for Volunteers to be challenged

8.1.2.4.1. Exclusion Criteria for Naïve Volunteers

- To be <18 or >45 years of age.

Justification: Volunteers<18 years of age can be exposed to an increased risk related to their growth and development, or loss of body weight in the presence of malarial infection. People > 45 years of age are at higher risk of unknown cardiovascular, cerebrovascular diseases or other relevant unknown diseases not detected by blood screening that can become evident during this trial due to the physical stress induced by the clinical presence of malaria.

- To be pregnant (as confirmed by laboratory test), breast-feeding, planning to be pregnant from the time of enrollment until six months after the challenge.

Justification: Infection with *P. falciparum* or *P. vivax* during pregnancy can be harmful for the mother and the fetus (Phillips-Howard, 1999). *P. vivax* infection during pregnancy has been associated with high levels of parasitemia (Singh *et al*., 1999), maternal anemia and low weight at birth (Nosten, 1999; Singh *et al*., 1999). Moreover, the immunological changes that take place during pregnancy and breast-feeding can affect the results of the study.

- To be Duffy negative red blood cell phenotype.

Justification: Duffy negative individuals are refractory to *P. vivax* infection.

- To have G6PD deficiency, as determined by quantitative, ultraviolet and kinetic analysis of glucose-6-phosphate dehydrogenase in whole blood, according to an internal standard operating procedure (AS-02-POE-083).

Justification: Primaquine, the first line of treatment against *P. vivax* malaria, cannot be administered to people with G6DP deficiency. The reactive oxygen species produced by the oxidative metabolism of this drug accumulate in red blood cells and cannot be eliminated due to the lack of the G6PD enzyme, and induces hemolytic anemia.

- To have any hemoglobin pathology.

Justification: Hb pathologies can also influence the development of *P. vivax*.

- To have a personal and family history of allergies to drugs or insect bites.

Justification: The parasite challenge involves exposure to mosquito bites and administration of drugs that, in susceptible individuals, can cause allergic reactions of diverse degrees of severity. Any potentially more serious risk for the volunteer, including the risk of anaphylaxis, should be avoided.

- To have a history of malaria infection or have received vaccination against malaria.

Justification: For naïve volunteers, any type of preexisting immunity against the parasite can alter the outcome of the malarial infection, which could lead to falsely assuming that the volunteer was not challenged with viable sporozoites.

- To have received a transfusion of any blood component within the 6 (six) months prior to the study.

Justification: The transfusion of any blood component can be a risk factor for infectious diseases, which could had been at a window period at the time of transfusion.

- To have clinical or laboratory abnormalities identified by (the) investigator(s).

Justification: Any abnormality in the baseline laboratory values could be indicative of a serious medical condition and could also affect the evaluation of AE during the study.

- *P. vivax* IFAT titers > 1:20 in the screening tests for naïve volunteers.
- To have lived in an endemic area for malaria during the 6 months prior to the challenge.
- Justification: Any type of pre-existing immunity to malaria could affect the outcome of the experimental infection, which could lead to falsely assuming that the volunteer was not challenged with viable sporozoites.
- To have clinical or laboratory evidence of a systemic disease, including psychiatric, hepatic, cardiovascular, pulmonary, renal disease or any other disease that can have a negative impact and affect the results of the study.
- To have evidence of active Hepatitis B, Hepatitis C or HIV infection.
- To have an autoimmune disease (Lupus, rheumatoid arthritis, thyroiditis or other).
- To be splenectomized
- To be under treatment with drugs that act on the immune system (steroids, immunosuppressive or immunomodulatory agents).

Justification: Immune deficiencies, immunosuppressive therapy, autoimmune diseases, chronic infections or any other relevant medical condition that could affect the immune response of the volunteer and thus the results of the experimental infection evaluated through this protocol. Moreover, exposure to infective mosquito bites and to malaria could increase the risks or severity of AE in people with preexisting diseases.

- Planning to have surgery within the time of recruitment and the post-challenge follow-ups.

Justification: Malaria infection could be an unnecessary risk for individuals that are to undergo surgery.

- To have a history of alcoholism or drug abuse, defined as a habit that interferes with normal social functioning.

Justification: The participation in this study requires the collaboration of the volunteers and therefore any condition that may alter the social functioning of the volunteer will interfere with the development of the study and expose participants to unnecessary risks.

- To have a condition that may interfere with the ability to provide free and voluntary IC.

Justification: All volunteers should be capable of completing the IC process freely and voluntarily, as declared by two witnesses that should also sign the IC form.

- To be participating as a volunteer in other research studies.

8.1.2.4.2. Exclusion Criteria for Pre-immune Volunteers

- *P. vivax* IFAT negative results (<1:20) in the screening tests.
- All other criteria for naive volunteers apply, except for to have lived in an area endemic for malaria for the past 6 months.

8.1.2.5 Selection

Individuals who meet each of the criteria for admission, as described in the inclusion and exclusion criteria, will be distributed according to their history of exposure to malaria in each of the (2) subsets (naive and pre-immune). Candidates who have been assessed and are eligible to participate will be randomly allocated in each group. Six people, 3 pre-immune and 3 naive volunteers will be included in the study as alternative volunteers, to ensure that a statistically appropriate number of volunteers participate in the day of the experimental challenge.

8.1.2.6. Study Procedures

**See the Study Procedures Diagram (Annex 1)**

8.1.2.6.1. Informed Consent

Volunteers willing to participate will be informed of the study objectives, procedures, risks and benefits, as well as about the rights and responsibilities of the study volunteers and participants. Each volunteer will be allowed to read the IC form and to ask questions to the study researchers to ensure their informed free and voluntary consent. The IC process will be registered in the folder of the participant. All volunteers need to be tested for HIV infection. Information about this test will be provided in a separate IC form, which the volunteer should sign before being enrolled in the study. All the volunteers will receive a copy of the IC form, which they have signed.

8.1.2.6.2. Identification

Volunteers will be assigned an identification code consisting of 4 digits. The first digit corresponds to the study number, the next two digits correspond to the sequential number of study entry and the fourth digit to the pre-immune or naive immune status of the volunteer, for which N (naive) or P is assigned (pre-immune), as applicable.

8.1.2.6.3. Initial Evaluation

Selection procedures (medical history, physical examination, and laboratory tests) will be made after the volunteers have signed the IC form. If volunteers have been evaluated and the challenge is not performed within the next 8 weeks, all screening tests will be repeated. At the screening visit, the relevant medical history and any concomitant therapy will be documented by one of the study physicians. A complete physical examination including examination of head, ears, neck, throat, eyes, cardiovascular, pulmonary, neurological, gastrointestinal, and dermatological systems will be performed. Twenty milliliters (20 mL) of blood and spontaneous urine will be collected for all laboratory tests described in this protocol, including urine pregnancy test.

8.1.2.6.4 Serology and Immunology Tests

Volunteers participating in the challenge will be tested for Hb level, hematocrit, reticulocyte and platelets count, blood group, G6PD and Hb electrophoresis, as well as renal function (BUN, creatinine, urinalysis), liver function (ASL, ALT, bilirubin), blood glucose, C-reactive protein, coagulation and β-HCG in women. Volunteers will be also screened for the following infectious diseases: HIV, HBV, HCV, HTLV-1 and 2, Chagas and RPR for syphilis. For malaria studies, volunteers will be tested by TBS and PCR for *Plasmodium* spp. In addition, anti-*P.vivax* antibody titers will be evaluated by indirect immunofluorescence (IFAT) was evaluated. ELISA tests will be performed with PvCS and Pv200L antigens to detect antibodies specific for these proteins. A fraction of leukocytes collected from each volunteer will be used to study the changes induced in the human transcriptome by the contact with the parasite.

8.1.2.6.5 Selection of *Anopheles albimanus* Mosquitoes for the Challenge

*An. albimanus* mosquitoes are reared in the insectary of the Entomological Unit in Cali and Buenaventura. About 5 and 15 lots of 2,000 mosquitoes (3-4 day-old) will be infected by the MFA technique (Internal SOP: EN-02-POE-001) 15 to 18 days prior to the experimental challenge, under strictly controlled conditions. Seven days after blood feeding, the rate of infection will be estimated by midgut dissection for oocyst count (EN-02-POE-002). Mosquito batches showing more than 50% of infection rate with an average of >1 midgut oocyst will be considered suitable for the challenge. The selected lots will be evaluated 14 days after blood feeding to determine the load of sporozoites, by dissection of salivary glands (EN-03-POE-001). The lot of mosquitoes for the challenge will be chosen from all batches fed on infected blood that meet above described criteria. The lot with the best rate of infection (sporozoite rate) will be selected. The production of several lots of infected mosquitoes on a weekly basis will ensure that the challenge is performed simultaneously. However, only one of the lots available will be used for the experimental challenge and the remaining lots will serve as alternative lots.

8.1.2.6.6 Maintenance of Infected Mosquitoes

Each lot of mosquitoes fed on infected blood will be maintained on the premises of the Entomology Unit in accordance with the provisions of the internal SOP EN-02-POE-001. Mosquitoes will be kept in cages that will be labeled as shown below:

| INFECTED MOSQUITOES FOR THE EXPERIMENTAL CHALLENGE  Donor volunteer ID:_________________________________________  Blood feeding Date:_________________________________________  Infectious Diseases Screening  Positive: ____________________ Negative: ____________________  Positive for: _______________________________________________  _________________________________________________________  Negative for other *Plasmodium* spp. different from *P. vivax*:_________  # mosquitoes per lot: ________________________________________  # mosquitoes with midgut dissection:____________________________  # infected mosquitoes: _______________________________________  # Oocysts: ________________________________________________  # mosquitoes with salivary gland dissection: _____________________  Sporozoite density:__________________________________________  Lot expiration day:___________________________________________ |
| --- |

8.1.2.6.7 Preparation for the Challenge

Volunteers will be invited to visit the Entomology Unit two (2) days prior to the challenge. This visit will allow them to become familiar with the place where the challenge will take place, thereby reducing the anxiety on the day of the challenge. **At this time, 35 mL of blood will be collected to get a baseline of their immune status.**

Volunteers women will be advised to immediately contact one of the study physicians if they become pregnant during the time spanning between the selection visit and the time of the experimental challenge. One day before the challenge, volunteer women will be asked to visit MVDC to take a urine pregnancy test. If the test is positive, the volunteer will be immediately excluded from the study. If any of the volunteers is pregnant, the volunteer will be replaced by one of the alternative volunteers.

On the day of the challenge, the volunteers will be evaluated by one of the study physicians, who will gather a clinical history and perform a short assessment that will include taking vital signs. If any of the volunteers is detected to have an acute illness, which in the opinion of the evaluator requires the exclusion from the study, the volunteer will be replaced by one of the alternative volunteers.

Mosquitoes of the lot chosen for the challenge will be distributed in small feeding cages (7 × 7 × 7 cm). Prior to the challenge, these cages will be prepared with three mosquitoes from the same lot per cage.

8.1.2.6.8 Sporozoite Challenge

The challenge will be conducted strictly under the protocol parameters and conditions set forth herein. It will take place in a safety room within the CAB Entomology Unit, specifically prepared for the study. The feeding cages will be placed on the forearm of the volunteer for 10 minutes, allowing for the feeding window, which is covered by a net, to be in contact with the skin of the volunteer.

Once the feeding time has elapsed, the volunteers will remain in the room, while the technicians of the Entomology Unit verify the number of mosquitoes that fed on blood and determine, by dissection of salivary glands and microscopic examination, the number of infected mosquitoes per cage and sporozoite load in the salivary glands. If the bite rate (determined by the presence or absence of blood meal) and the infectivity rate (determined by the load of sporozoites in the salivary glands) is below the minimum number of infective bites expected, an additional feeding cycle will be performed with additional mosquitoes until completing 3 ± 1 sporozoite-infected fed mosquitoes. For example, if only two mosquitoes fed on a volunteer’s blood and both were positive for sporozoites in the salivary glands, no more bites would be required. If on the contrary the volunteer is found have been bitten by only one infective mosquito, two new mosquitoes will be used in the second feeding cycle.

Volunteers will be observed directly by one of the study physicians for about an hour after the challenge. This will allow detecting any adverse reactions induced by the mosquito bites immediately. Approximately eight hours after the challenge, volunteers will be contacted by phone to document the evolution. Volunteers will be provided all the necessary information to contact the researchers at any time of the day (including a cell phone number) and will be encouraged to call if they have questions or need guidance. This direct contact will continue for three weeks.

8.1.2.6.9 Post-Challenge Medical Management and Follow up Visits

**Follow up during the period of pre-patent parasitemia:** From day one until day 6 post-challenge, volunteers will be followed by phone by the study staff. Volunteers will be instructed on malaria symptoms such as fever, headache, chills, myalgia and malaise, which may occur between days 7 and 23 after the challenge. Although it is unlikely for blood parasitemia to appear before the ninth day, the study team will be available to provide medical attention to any one presenting early symptoms of malaria or other discomfort.

8.1.2.6.10 Diagnosis

8.1.2.6.10.1 TBS Diagnosis

Malaria will be diagnosed by TBS from finger-prick blood. TBS slides will be stained by the Giemsa method and examined under the microscope independently by two expert microscopists whowill have no knowledge of the volunteers, in order to ensure a blinded diagnosis procedure. Slides will be labeled with the volunteer’s ID code and kept in case a new examination is necessary. At the time of *P. vivax* diagnosis, a complete physical exam will be performed to each of the volunteers and 30 mL of blood will be collected for hematological, chemical and immunological tests.

8.1.2.6.10.2 Xenodiagnostic and Assessment of Gametocyte Infectivity:

Infectivity of *P. vivax* gametocytes in the blood of the challenged volunteers will be studied by exposure to healthy *Anopheles* mosquitoes to test the hypothesis that these parasite stages (gametocytes) develop early during the blood cycle, even before being microscopically detectable in peripheral blood, after day 7 post-infection. For this purpose, direct mosquito feeding will be compared with membrane feeding. Mosquitoes will be used since these have been reported to detect parasites (xenodiagnosis) earlier than diagnostic laboratory tests for other communicable diseases such as Chagas disease, trypanosomiasis, leishmaniasis and Harbovirus infections (Mourya, Gokhale et al. 2007; Wombou Toukam, Solano et al. 2011).

Volunteers will be exposed to the direct bites of 20 uninfected *An. albimanus* mosquitoes according to SOP EN-02-POE-003. Briefly, mosquitoes are placed in 7 × 7 × 7 cm feeding cages, which are placed on the forearm of each individual for a period of 5 minutes. Mosquitoes are then evaluated for the presence of oocysts on day 7 according to SOP EN-02-POE-002, and on day 14 for the presence of sporozoites according SOP EN-03-POE-001. Direct exposure to mosquitoes will take place every third day starting on day 7 until the microscopic diagnosis is confirmed, e.g., day 7, 9, 11, etc.

8.1.2.6.11 Follow up

Volunteers will be examined daily by a study physician at MVDC headquarters in Cali, from day 7 after the challenge until day 23. After this day, they will be examined every other day until the 29th. During follow-up examination, volunteers will be questioned about the presence of the previously mentioned symptoms, a complete medical evaluation and a TBS will be performed and the data obtained from these evaluations will be kept in the corresponding CRF. If the volunteer has fever (axillary temperature >38°C), TBS samples will be taken twice a day. If the volunteer has symptoms compatible with malaria, but the TBS is repeatedly negative (2-3 times), a PCR test will be perform to detect Plasmodium spp. (Snounou *et al*., 1993), which will clarify the diagnosis immediately. However, the gold standard for malaria diagnosis will be TBS. Simultaneously to TBS testing, blood samples will be collected on filter paper and stored at -20°C to perform quantitative PCR analysis of parasitemia for comparative purposes at the end of the trial.

During the first 28 days following the experimental challenge, the research team will be available 24 hours a day in order to ensure medical attention to the volunteers at any time of day. If by day 28 after the challenge, a volunteer has not developed infection, he will be treated by day 29 with chloroquine and primaquine as described below. A complete physical exam, hematology and chemistry laboratories will be taken 30 days after completing the drug treatment.

If contact with any of the volunteers is lost prior to treatment administration, an active search will be carry out by calling his/her contact numbers, the guardian appointed at study enrollment, and as a last resource, visit by study staff to the residence or work place of the volunteer.

If any of the volunteers requires intra-hospital care, this will be provided at a level III facility in Cali for the duration of the study and for 6 months after its completion, in order to ensure timely treatment of any possible malaria relapses.

8.1.2.6.12 Treatment

Once malaria infection is detected, volunteers will be treated with antimalarial drugs approved by the Colombian Ministry of Health. This treatment consists of chloroquine (total dose of 1500 mg divided into three (3) doses: an initial dose of 600 mg, followed by 450 mg administered 24 and 48 hours later, together with primaquine (30 mg/day, two tablets per day for 14 days).

All drugs will be provided and supervised by MVCD staff and administered with food to avoid gastrointestinal disturbances such as nausea and vomiting, which may occur when these drugs are administered on an empty stomach.

Although the standard dose of primaquine in Colombia is 15 mg/day, this is not always sufficient to prevent the activation of hypnozoites; therefore, primaquine treatment will be given at a dose of 30 mg/day. Volunteers who do not show parasitemia by day 29 will be treated with the same drug scheme starting on that day. This drug regimen was proven effective in the two previous challenges.

If a volunteer woman becomes pregnant during the duration of the study, treatment will be limited only to chloroquine, in the same doses established above. Neither primaquine nor Falcidar® should be administered to pregnant women. In the event that resistance to chloroquine is reported, amodiaquine treatment will be administered as described before in this protocol.

8.1.2.6.12.1 Chloroquine Resistance

There are no reports of resistance to the combined treatment of *P. vivax* malaria with chloroquine plus primaquine in Colombia, (Soto *et al*., 2001). However, in the unlikely event of a positive TBS sample at any time during the post-treatment follow up (on days 7, 14, or 28 after the first dose), the volunteer will receive alternative treatment with Falcidar® (SP), administered in three tablets in a single dose (25 mg pyrimethamine/500 mg sulfadoxine per tablet). If the volunteer has any contraindication to the administration of Falcidar® (e.g. sulfa allergy), treatment should be given with amodiaquine as described before, and will be followed by additional TBS to confirm complete cure.

8.1.2.6.13 Monitoring of Antimalarial Treatment Initiation

Samples for TBS will be taken daily samples from the start of treatment with chloroquine until three consecutive negative results are obtained. Follow-up with TBS will continue on days 7, 14 and 28 after initiation of treatment to ensure that the infection had been resolved. If any of the volunteers develops fever or other symptoms of malaria, TBS will be performed on every other day to document the persistence of infection. Additionally, 3 weeks and 6 months after completion of treatment a complete physical examination will be performed by the study physician and 30 mL of blood will be collected for hematological, chemical and immunological laboratories.

8.1.2.6.14 Monitoring for Parasitemia Relapse and Recrudescence.

There have not been documented cases of a relapse with supervised treatment under high doses of primaquine (30 mg/day for 14 days) (Baird *et al*., 2002*.* Hoffman *et al*., 2002). Volunteers participating in this study will receive this treatment scheme on the premises of MVDC*.* In previous challenge trial, the 18 volunteers were monitored for 1 year. Only an individual, who traveled to a malaria endemic area during this period, showed malaria symptoms and was positive for TBS. The patient responded satisfactorily to treatment (Herrera *et al.*, 2009). Any *P. vivax* relapse will be treated with a new course of chloroquine and primaquine (identical to the first cycle of treatment), follow-up will be conducted on the same days as for the first cycle, as explained before*.* Long-term monitoring for possible relapses of *P.vivax* hypnozoites will be performed. Once the supervised primaquine treatment is completed (2 weeks), with negative post-treatment TBS, all volunteers will be contacted by telephone like defined in Table 1.

| Post-treatment week for telephone contact | Accepted window |
| --- | --- |
| Week 1, 2, 3 and 4 | ± 3 days |
| Week 6 and 8 | ± 5 days |
| Week 10 and 12 | ± 7 days |
| Week 16, 20, 24 | ± 10 days |

**Table 1**: Telephone follow-up after treatment

On the other hand, all the volunteers who have been challenged will be instructed to contact any of the study physicians in case of fever or other symptoms of malaria outside the time allocated for telephone monitoring or during a year and a half after the challenge. If the volunteer were to consult a health care provider institution, communication with the attending physician will be established to guide the diagnosis and treatment, if necessary. If during the year and a half after the challenge a volunteer develops fever, chills, or other malaria related symptoms, the physician who is evaluating the volunteer must be properly informed about the experimental exposure to malaria and it would be recommendable to perform TBS diagnosis to the volunteer.

8.1.2.6.15 Travel Restrictions.

Volunteers will receive instructions not to travel to malarious areas from the day of the challenge up to four weeks after treatment.

This time interval can be divided into two periods of low and high risk. If a volunteer must travel during the low-risk period (day 1 to day 6 or 22 to 28) all efforts will be made to ensure maximum contact between the volunteer and researchers (by cellphone, pagers, etc.). If the destination where the volunteer is traveling is located far away, the local Health Promoting Company (namely in Colombia *Entidad Prestadora de Salud – EPS*) will be contacted so that the volunteer receives medical attention during the trip. If the study physician considers that adequate coverage cannot be ensure, the volunteer will be withdrawn from the study, and given antimalarial treatment before the trip, according to the scheme described in the protocol for people who completed the study. If any of the volunteers must travel for emergency reasons during the high risk period (day 7 to day 21), they will be treated for malaria immediately before traveling (medical follow up will be made as described above) unless a close medical monitoring can be ensured.

8.1.2.6.16 Duration of Step B Study*.*

This step of the study will have a total duration of 6 months from the time of enrollment of volunteers. The recruitment of volunteers for the challenge (Step B) will be performed before enrollment of *P.vivax*-infected blood donors (Step A).

8.1.3 Sample Collection, Processing and Storage

A maximum of 30 mL of blood will be obtained before the infectious challenge, the day of the positive malaria diagnosis, at 3 weeks and 6 months after treatment completion, which will be used for immunological studies in accordance with MVDC SOPs. Once labeled with the identification code of each volunteer, samples will be immediately transported to the MVDC laboratories for analysis. Samples will be stored at MVDC, if authorized by the volunteer. All TBS slides will be taken and examined at MVDC.

8.1.4 Data Management and Data Analysis

All data will be registered into a database designed with Redcap (Vanderbilt University, 2012), which has password access restricted. Data will be uploaded by the study staff on an Electronic Case Report Form, verified by the clinical monitor according to the standard operating procedures, and corrected if necessary by the authorized investigator. CRF data verification will be done using the source documents as reference and comparing it to the information printed from the database. The electronic CRF will not be considered as a source document in any case. The Clinical Monitor will report the inconsistencies to be checked and corrected by authorized personnel*.* After QC is completed, information will be analyzed in Stata^TM^ 9.1. This analysis will be performed using appropriate statistical tests according to the type and distribution of the variables. The significance level of α = 0.05 will be considered for statistical tests*.*

Differences between comparison groups when the variable is dichotomous will be analyzed by the Chi^2^ test (Fisher's exact test was used when the number of data is less than 5). Group comparison of continuous variables will be done using student t test. Comparison between various groups will be made by ANOVA analysis (Scheffe’s assessment for post-hoc analysis).

8.1.5 Record-keeping

During the course of the study, the CRFs, source documents, IC forms, inclusion questionnaires and all relevant information of the volunteers will be kept in a safe place at MVDC. The electronic database will be stored in non-rewritable optical media. The records of the participants will be transported by authorized staff in a portable, secure and water-proof case (MVDC: Carrera 37 2Bis No.5E -08, Cali, Colombia). Once these documents have been used, they will again be kept at MVDC. At the end of the study, all reports, consent forms, questionnaires and other relevant records of the protocol will be filed at MVDC for a period of 10 years, after which they will be identified as dead file.

9. RISKS FOR VOLUNTEERS, RESEARCH GROUP AND ENVIRONMENT - PRECAUTIONS TO MINIMIZE RISK

Through the *P. vivax*-sporozoite challenge system described here, our group has exposed 35 healthy naive volunteers to infection in two consecutive trials, which proved to be safe at doses of 2–10 infective bites. Infections showed prepatent periods ranging from 9–18 days with an average of about 11 days. The duration of symptoms was similar in all volunteers (1.5–4.5 days) and treatment responses were rapid and similar in all volunteers. All volunteers cleared parasitemia within the first 48 hours after treatment (Herrera *et al*., 2009; Herrera *et al*., 2010). In our previous studies, the prepatent period was evaluated by PCR and TBS from day 7. In some cases, PCR was able to detect parasitemia before TBS but in neither case did PCR detected parasitemia before day 9 post-challenge. TBS was sensitive, detecting parasitemia levels as low as those described above (geometric mean of 46 parasites/µL). Likewise with *P. falciparum*, hundreds of volunteers have been infected in a safe and reproducible way in the USA, 97% of these volunteers developed mild symptoms of short duration (median duration: 3 days) (Hoffman, 1997). These volunteers were treated without complications due to the timely initiation of treatment, when parasitemia was still low (geometric mean of 46/μL parasites) and because parasite sensitivity the administered antimalarial was known.

Spleen rupture is a rare event, which has been observed only in patients with chronic infection (Yagmur *et al*., 2000). In the previous challenge study, only one of 17 volunteers showed splenomegaly as an adverse event related to the infection (Herrera *et al*., 2010). In the proposed study, volunteers will be followed closely and treated immediately as soon as parasitemia is detected.

9.1 RISKS FOR VOLUNTEERS DONATING BLOOD.

The potential risks associated with blood donation may include redness, itching, infection at the puncture site or vasovagal symptoms such as dizziness and fainting. Blood samples will be collected by venipuncture under aseptic and antiseptic conditions, using new, disposable sterile material. A study doctor will be present to provide primary care, if necessary, e.g. to treat vasovagal episodes (fainting).

A short delay (10–15 minutes) in receiving the first dose of antimalarial drugs may be associated with blood donation, but this risk does not significantly affect the recovery of the volunteer. Every effort will be made to expedite procedures so that antimalarial treatment can be started as quickly as possible. A complete blood count will be performed in order to detect early blood disorders related to malaria.

Improper handling of an HIV positive result that results on a negative impact in the volunteer’s personal environment and/or occupational hazard is a potential risk. To minimize this risk, strict compliance to confidentiality policies will be enforced. Volunteers will personally receive a copy of laboratory results one week after blood analysis is completed. Whenever there is a positive result for any of the infectious diseases screened for in this study, the volunteer will be referred for counseling and medical care to the healthcare provider institution as stipulated in Law 100, Article 179, 1993 or to the Departmental Health Secretariat in accordance with Law 1543, 1997, Chapter II of the Ministry of Health. If a volunteer already has a health insurance, he/she will be referred to their private physician with the test results. These results will only be delivered to the volunteer.

9.2 MALARIA CHALLENGE ASSOCIATED RISKS FOR THE VOLUNTEER

The risks associated with the challenge include a very low risk of anaphylaxis, the possible transmission of other infectious agents by mosquito bites and the risk associated with the use of antimalarial drugs.

**Precautions to minimize the risks associated with malaria challenge:**

- **Anaphylaxis management:** At the facilities where the challenge is to take place, there will be medicines available for the management of anaphylaxis, such as epinephrine 1:1000, diphenhydramine cimetidine and methyl-prednisolone, which will be administered by the research doctor who will be accompanying the volunteers during the mosquito bite procedure*.* An ambulance will be available and will be used whenever necessary to transport the volunteer from the Entomology Unit to an Acute Care Center, which takes about 30 min*.*
- **Blood screening:** Donors of *P. vivax-*infected blood will be screening for infectious diseases as described above.
- **Volunteer selecting and monitoring:** Volunteers selected for the challenge have to meet each of the criteria. Volunteers will be closely monitored after the challenge and will be treated once the infection is detected in blood, as described before in this protocol. The early initiation of antimalarial treatment will minimize the risk of developing serious complications, which are usually rare in *P. vivax* infections*.* In order to ensure proper follow up of volunteers, each one of them will have access to the contact information of the research team*.* A delay in malaria diagnosis is very unlikely to occur under the carefully controlled condition of this study. During infection, some transient abnormalities may present such as fever, headache, myalgia, nausea, vomiting, mild anemia, leucopenia, thrombocytopenia and fatigue, which are highly unlikely when infection is diagnosed early. In healthy adults, the only serious risk directly associated with *P. vivax* infection is splenic rupture (Yagmur *et al.*, 2000), which is highly unlikely if treatment is given immediately as soon as the disease is diagnosed. However, as a precaution, volunteers will be informed of this risk and should be advised to avoid contact-sports or any strenuous activities that may result in abdominal trauma especially during week two, following initiation of treatment.
- **Pregnancy and *Plasmodium vivax* infection:** Although the effects of *P. vivax* malaria during pregnancy are less severe than those caused by *P. falciparum* (Nosten *et al*., 1999), *P. vivax* infection during pregnancy is associated with high maternal parasitemia (compared with parasitemia in nonpregnant women), maternal anemia and low weight at birth (Nosten *et al.*, 1999*.* Singh *et al*., 1999). Women will be instructed to use appropriate contraception until three months after the challenge. Women will be prompted to inform to the study physician if they became aware of being pregnant during the study. If any of the women has a relapse by *P. vivax* while pregnant, she will be given immediate treatment, which significantly reduces the risk for the mother and fetus to have an adverse outcome during pregnancy. Chloroquine is safe during pregnancy (McGready *et al*., 2002), same as amodiaquine in case alternative antimalarial therapy is necessary*.* Primaquine treatment will be administered after pregnancy.
- **Relapses:** Antimalarial drug therapy consisting of chloroquine and high doses of primaquine will be administered under medical supervision. No cases of *P.vivax* relapses have been documented with the administration of a chloquine/primaquine combined regime (Baird, et al. 2002; Hoffman, et al., 2002). Volunteers will be follow-up closely after treatment.

9.3 ANTIMALARIAL TREATMENT ASSOCIATED RISKS FOR THE VOLUNTEERS

Potential side effects resulting from the use of antimalarial drugs include nausea, vomiting, diarrhea, abdominal pain, dizziness, headache, sleep disturbances, blurred vision, itching, tinnitus, and photosensitivity*.* The following adverse reactions have been reported by the FDA in regard to the use of antimalarial medication:

- **Chloroquine phosphate:** Gastrointestinal reactions (vomiting, nausea, diarrhea, cramps), mild transient headache, auditory effects such as nerve deafness, tinnitus and decreased hearing acuity in those with a history of it. There may also be visual effects, dermatologic reactions (pruritus and alopecia) and cardiovascular effects (hypotension or changes in the EKG). Chloroquine is advise in patients with psoriasis or other dermatological disease.
- **Primaquine:** Side effects most frequently encountered are those related to gastrointestinal disturbances such as nausea and abdominal discomfort, especially if the drug is administered on an empty stomach. Primaquine would be administered with food intake in this study*.* Primaquine has been reported to cause leucopenia and in most individuals induces slight methemoglobinemia*.* Concomitant use of substances that predispose to this side effect such as sulfonamides should be avoided. Primaquine is not recommended for pregnant women. Administering a dose of 30 mg primaquine per day for more than a year in healthy adults has been shown to be well tolerated provided it is accompanied by food intake. No significant effects related to kidney or liver damage, as evidenced by serum creatinine, BUN, AST, ALT, LDH, alkaline phosphatase. Methemoglobinemia is reversible and asymptomatic (Fryauff *et al*., 1995).
- **Falcidar®** **(sulfadoxine–pyrimethamine):** Toxic manifestations are rare and usually attributable to the sulfadoxine component*.* Severe skin reactions (erythema multiforme, Stevens-Johnson syndrome and toxic epidermal necrosis) have been reported in individuals using a weekly schedule for prophylaxis. The safety of this combination during pregnancy has not been established, but the drug has been used to treat a large number of pregnant women.
- **Amodiaquine:** Adverse reactions of amodiaquine are generally similar to those of chloroquine. The most common are nausea, vomiting, abdominal pain, diarrhea and itching; a less common effect is bradycardia. There is evidence that pruritus is less common with amodiaquine than with chloroquine.

**Treatment or precautions:** Volunteers will receive antimalarial treatment under medical supervision, which will allow for close monitoring to detect side effects*.* AE will be documented as well as the potential association to the treatment, which will receive a score of causality.

In the previous challenge, gastrointestinal-related events were the more frequent AE associated with the antimalarial treatment (nausea, dizziness and epigastric pain). Symptoms reported by the volunteers did not affect normal daily activity.

9.4 RISK FOR THE STUDY STAFF

There is a low risk for workers who are responsible for sample collection and processing due possible accidents with volunteer’s needles.

- **Precautions for staff:** To reduce the risk, all employees who have contact with blood samples must strictly follow standard precautions. In addition, volunteers who are positive for HIV, hepatitis B or hepatitis C will be excluded from the study.

9.5 RISKS AND PRECAUTIONS RELATED TO THE ENVIRONMENT

The risk of accidental transmission of malaria to anyone in the community is negligible. Infected mosquitoes will be kept at restricted areas of the insectary and will not be removed from it at any time. Infections in the study volunteers will be treated quickly before gametocytes can develop (this is usually 10 days after parasites first appear in the blood). Volunteers may only be staying in Cali, which is not an endemic area so that natural transmission of malaria does not occur*.* If any member of the group is accidentally bitten by an infected mosquito or develop symptoms of malaria, he/she will be evaluated immediately by TBS to confirm the presence of infection. If the result is positive, the standard treatment, chloroquine and primaquine will be given under medical supervision.

10. BENEFITS

10.1 BENEFITS FOR VOLUNTEERS OF STEP A

There will be no direct benefits for the volunteers participating in this study. However, volunteers can receive some indirect benefits as result of having their blood screened for infectious diseases. If a volunteer is detected to be positive for any infectious disease other than malaria, including HIV, the volunteer will be provided with a copy of the test results and referred for counseling and medical assistance to his health care provider institution (*Institución Prestadora de Salud*) according to the healthcare regimen to which the volunteer is subscribed. Volunteers will not receive any economic benefit as result of their participation in the study.

10.2 BENEFITS FOR VOLUNTEERS OF STEP B

There are no direct benefits for the volunteers participating in this study. However, volunteers can receive some indirect benefits as result of being screened for infectious diseases. If during the selection phase a volunteer is detected to be positive for any infectious disease, including HIV, the volunteer will be provided with a copy of the test results and referred for counseling and medical assistance to his health care provider institution (*Institución Prestadora de Salud*) according to the healthcare regimen to which the volunteer is subscribed to, in accordance with Law 100, Article 179 of 1993, or to the Departmental Health Secretariat, according to Law 1543 of 1997, Chapter II, issued by the Colombian Ministry of Health.

11. COMPENSATION

11.1 COMPENSATION FOR VOLUNTEERS OF STEP A

Volunteers will not incur in economic expenses as result of the participation in this study; however, as stipulated by the Colombian Ministry of Health, no monetary compensation should be provided to the volunteers. The study physicians will offer medical counseling to volunteers that are diagnosed with a disease different from malaria. Volunteers will be reimbursed for transportation expenses, received a complete medical evaluation and counseling if any additional pathological condition is detected. Volunteers will be provided with a snack after blood donation procedures.

11.2 COMPENSATION FOR VOLUNTEERS OF STEP B

There will be no direct compensation for the volunteers due to the participation in this study. However, volunteers will receive indirect benefits such as being screening for infectious diseases and blood conditions. The volunteers will not incur in any economic expenses as result of the participation in this study, therefore transportation costs will be reimbursed and snacks will be provided on the day of the challenge and on the days of medical follow up. Additionally, each time a volunteer visits MVDC for study related procedures, he/she will be given an amount of money corresponding to the daily legal minimum wage as a symbolic compensation for the time committed to the development of the study.

**12. CRITERIA FOR WITHDRAWAL OF VOLUNTEERS FROM THE STUDY**

Volunteers may withdraw freely from the study at any time. If a challenged volunteer chooses to leave the study, he/she will receive antimalarial treatment at that time as described in this protocol. If a volunteer is excluded from the study for any reason, a final evaluation (physical and laboratory tests) will be performed. The reason for the withdrawal of any of the volunteers will be reported in the corresponding CRF and will be accompanied by supporting information.

13. ADVERSE EVENTS

An AE is considered as any sign, inconvenience, damage and dysfunction, adverse reaction to any medication or other undesirable outcome that occurs in any of the volunteers participating in the study, including those that have already been defined as expected risks. Each of these events will be reported in a CRF, and will be assigned a degree of severity and causality related to the study activities (e.g. blood donation or malaria challenge).

The intensity of the AEs registered in the CRF will correspond to the higher degree reached during an episode. For example, if a person has fever, the degree of intensity is assigned according to the highest temperature that was reached.

AE will be divided into two groups: asked and unasked

• **Asked AEs:** Researchers will ask for these AEs in all contacts with the volunteers and the information will be registered in the CRF of the corresponding study interval as described below.

o **Local AEs:** AEs occurring in the part of the body that was exposed to mosquitoes during the sporozoite challenge. These will be asked for from the time of the challenge until 7 days post-challenge.

All local AEs will be registered in the corresponding CRF and its intensity will be classified according to the table shown below, which was adapted from "Guidance for Industry - Toxicity Grading Scale for Healthy Adult and Adolescent Volunteers Enrolled in Preventive Vaccine Clinical Trials (FDA, 2007) ".

| **Local AEs** | | | | |
| --- | --- | --- | --- | --- |
| **Local Reaction** | **Grade 1** | **Grade 2** | **Grade 3** | **Grade 4** |
| Pain | Does not interfere with activity | Repeated use of non-steroidal anti-inflammatory drug >24 hrs or interferes with activity | Any use of narcotic pain reliever or prevents daily activity | Emergency room (ER) visit >12 hrs or hospitalization |
| Tenderness | Mild discomfort to touch | Discomfort with movement | Significant discomfort to rest | ER visit for > 12 hrs or hospitalization |
| Erythema | 2.5 – 5 cm | 5.1 –10 cm | > 10 cm | Necrosis or exfoliative dermatitis |
| Induration | 2.5 – 5 cm and does not interfere with activity | 5.1 – 10 cm or interferes with activity | > 10 cm or prevents daily activity | Necrosis |

o **Systemic AEs:** These will be asked for from the time of the challenge until 7 days after completing the antimalarial treatment. Systemic AEs may be due to the body's reaction to the challenge or the administration of antimalarial drugs. AEs occurring from the time of the challenge to the time of malaria diagnosis will be attributed to *P. vivax* infection. AEs occurring from the time of antimalarial drug treatment until 7 days after completing the drug treatment will be attributed to the drug. This 7-day margin after completing the drug treatment was chosen given that during this time there are sub-therapeutic drug levels circulating in the body.

All asked systemic AEs will be registered in the corresponding CRF according to the table shown below, which was adapted from "Guidance for Industry - Toxicity Grading Scale for Healthy Adult and Adolescent Volunteers Enrolled in Preventive Vaccine Clinical Trials (FDA, 2007)".

| **Systemic Asked AEs** | | | | |
| --- | --- | --- | --- | --- |
| **Systemic Reaction** | **Grade 1** | **Grade 2** | **Grade 3** | **Grade 4** |
| Disease or clinical AE | No interference with activity | Some interference with activity but does not require medical intervention | Interference with daily activity and requires medical attention | ER visit for > 12 hrs or hospitalization |
| Nausea | No interference with activity | Interference with activity | Interferes with daily activity | ER visit for > 12 hrs or hospitalization |
| Vomiting | 1 – 2 episodes | > 2 episodes | Requires outpatient IV hydration | ER visit for > 12 hrs or hospitalization |
| Diarrhea | 2 – 3 loose stools | 4 – 5 loose stools | 6 or more watery stools or requires outpatient IV hydration | ER visit for > 12 hrs or hospitalization |
| Headache | No interference with activity | Repeated use of non-steroidal anti-inflammatory drugs > 24 hrs or some interference with activity | Any use of narcotic pain reliever or interference with daily activity | ER visit for > 12 hrs or hospitalization |
| Fatigue | No interference with activity | Some interference with activity | Significant; prevents daily activity | ER visit for > 12 hrs or hospitalization |
| Myalgia | No interference with activity | Some interference with activity | Significant; prevents daily activity | ER visit for > 12 hrs or hospitalization |

**Vital Signs**

| **Vital Signs** | **Grade 1** | **Grade 2** | **Grade 3** | **Grade 4** |
| --- | --- | --- | --- | --- |
| Fever (°C) | 38 – 38.4 | 38.5 – 38.9 | 39 – 40 | > 40 |
| Tachycardia  (beats/min) | 101 – 115 | 116 – 130 | > 130 | ER visit for > 12 hrs or hospitalization |
| Bradycardia (beats/min) | 50 – 54 | 45 – 49 | < 45 | ER visit for > 12 hrs or hospitalization |
| Hypertension (systolic) mm Hg | 141 – 150 | 151 – 155 | > 155 | ER visit for > 12 hrs or hospitalization |
| Hypertension (diastolic) mm Hg | 91 – 95 | 96 – 100 | > 100 | ER visit for > 12 hrs or hospitalization |
| Hypotension (systolic) mm Hg | 85 – 89 | 80 – 84 | < 80 | ER visit for > 12 hrs or hospitalization |
| Respiratory rate (breaths/min) | 17 – 20 | 21 – 25 | > 25 | Intubation |

**Serum**

| **Serum** | **Grade 1** | **Grade 2** | **Grade 3** | **Grade 4** |
| --- | --- | --- | --- | --- |
| Glucose – Hypoglycemia (mg/dL) | 65 – 69 | 55 – 64 | 45 – 54 | < 45 |
| Glucose – Hyperglycemia  Random (mg/dL) | 110 – 125 | 126 – 200 | > 200 | Insulin requirements or hyperosmolar coma |
| Blood Urea Nitrogen  BUN (mg/dL) | 23 – 26 | 27 – 31 | > 31 | Requires dialysis |
| Creatinine (mg/dL) | 1.5 – 1.7 | 1.8 – 2.0 | 2.1 – 2.5 | Requires dialysis |
| ALT, AST  increase by factor | 1.1 – 2.5 × ULN | 2.6 – 5.0 × ULN | 5.1 – 10 × ULN | > 10 × ULN |
| Bilirubin – when accompanied by any AST/ALT increase by factor | 1.1 – 1.25 ×ULN | 1.26 – 1.5 × ULN | 1.51 – 1.75 × ULN | > 1.75 × ULN |
| Bilirubin – when AST/ALT is normal; increase by factor | 1.1 – 1.5 × ULN | 1.6 – 2.0 × ULN | 2.0 – 3.0 × ULN | > 3.0 × ULN |

**Hematology**

| **Hematology** | **Grade 1** | **Grade 2** | **Grade 3** | **Grade 4** |
| --- | --- | --- | --- | --- |
| Hb (Woman) – gm/dL | 11 – 12 | 9.5 – 10.9 | 8.0 – 9.4 | < 8 |
| Hb (Man) – gm/dL | 12.5 – 13.5 | 10.5 – 12.4 | 8.5 – 10.4 | < 8.5 |
| Leucocytosis – cells/mm^3^ | 10,800 – 15,000 | 15,001 – 20,000 | 20,001 – 25,000 | > 25,000 |
| Leucopenia – cells/mm^3^ | 2,500 – 3,500 | 1,500 – 2,499 | 1,000 – 1,499 | < 1,000 |
| Lymphopenia – cells/mm^3^ | 750 – 1,000 | 500 – 749 | 250 – 499 | < 205 |
| Neutropenia – cells/mm^3^ | 1,000 – 1,499 | 500 – 999 | 499 – 300 | < 300 |
| Eosinophils – cells/mm^3^ | 650 – 1,500 | 1,501 – 5,000 | > 5,000 | Hypereosinophilia |
| Thrombocytopenia – cells/mm^3^ | 125,000 – 140,000 | 100,000 – 124,000 | 25,000 – 99,000 | < 25,000 |
| PT – increase by factor  (prothrombin time) | 1.0 – 1.1 × ULN | 1.11 – 1.20 × ULN | 1.21 – 1.25 × ULN | > 1.25 × ULN |
| PTT -– increase by factor (thrombosplastin time) | 1.0 – 1.2 × ULN | 1.21 – 1.4 × ULN | 1.41 – 1.5 × ULN | > 1.5 × ULN |

**Note**: The lower cutoff point for neutrophils is set below the reference range because of the association between benign ethnic neutropenia in African-descent populations.

**Urine**

| **Urine** | **Grade 1** | **Grade 2** | **Grade 3** | **Grade 4** |
| --- | --- | --- | --- | --- |
| Proteinuria | Trace | 1+ | 2+ | Hospitalization or dialysis |
| Glucosuria | Trace | 1+ | 2+ | Hospitalization or hyperglycemia |
| Hematuria (microscopic) – red blood cells/field | 1-10 | 11-50 | > 50 or macroscopic | Hospitalization or packed red blood cells transfusion |

- **Non solicited AEs:** These are AEs that were not foreseen among the asked AEs. All these AE will be registered on a CRF from the time of the challenge until 7 days after the end of the antimalarial treatment. After this time, only AEs related to the antimalarial treatment or suspected to be related to malaria will be registered in the corresponding CRF.

For volunteers visiting a malaria endemic area, a blood sample will be collected in filter paper, in addition to the routine TBS diagnosis, in order to define whether it is a new malaria infection or a relapse due to the clinical trial.

The severity of the non-solicited AEs will be graded according to the values assigned to symptoms, signs and laboratory test results in common toxicity criteria. AEs will be graded according to the criteria of the attending physician and the principal investigator (PI) based on “Guidance for Industry – Toxicity Grading Scale for Healthy Adult and Adolescent Volunteers Enrolled in Preventive Vaccine Clinical Trials (FDA, 2007). If there is any alteration that requires of additional paraclinical or clinical studies, these will be performed, same as for controls already established, and will be monitored until parameters return to normal values. The severity of the symptoms will be graded according to the definitions shown below:

• Grade 1= Mild

• Grade 2= Moderate

• Grade 3= Severe

• Grade 4= Potentially life threatening

Mild: It is a transitory, self-limited event accompanied by the presence of minor symptoms that do not interfere with the normal activity of the individual (e.g. the volunteer is able to work or study) and does not require any medical intervention. For example: pain and erythema (site of mosquito bite); myalgia (malaria).

Moderate: Events requiring minimal medical intervention to improve the health conditions of the volunteer. In these cases, once the intervention is completed, the volunteer is expected to perform routine activities, although there may be a degree of functional limitation. For example: itching and/or sufficient pain to limit normal moment (site of mosquito bite); fever that improves with anti-inflammatory drugs (malaria).

Severe: Symptoms that require treatment and prevent normal performance of daily activities. Volunteers with serious AEs usually cannot work, but can be managed safely as outpatients. For example: flu-like symptoms or fever that results in prostration (malaria).

**Potentially life threatening: Any event that results in visit to the emergency room (ER) for more than 12 hours or requires hospitalization. For example: bronchospasm requiring parenteral medication in ER or seizures attended in ER but not requiring hospitalization.**

13.1 SERIOUS ADVERSE EVENTS:

SAE will be reported according the classification guidelines for the Americas (WHO/PAHO 2005) as follows:

1. Results in death

2. Life threatening, requires hospitalization of the patient or prolongation of existing hospitalization

3. Results in persistent or significant disability, or congenital anomaly at birth

13.2 AE CLASSIFICATION – RELATION TO STUDY ACTIVITIES

According to GCP standards, AEs may occur during any interaction with the volunteer, including the time of screening and selection processes as well as during the study procedures or subsequent clinical follow-up. Each of the events will be classified as probably related, probably not related, possibly related or not related to the study activities (e.g. blood sampling, challenge or antimalarial treatment). This classification will be done according to the medical criteria of the PI in coordination with other evaluators of adverse medical events. Any AE that may arise during the course of the study will be reported, whether or not considered to be related to the infective mosquito bites. These definitions include concomitant diseases, injuries and exacerbations of preexisting conditions.

Degrees of causality:

1. *Not related*: The event does not have a temporal relationship to the participation of the volunteer in this study and is definitely associated with other etiology.
2. *Probably not related*: Time of onset and the nature of the event are not temporally related to the research intervention.
3. *Possibly related*: The time and nature of the AE may be the result of the participation in this study, but another explanation is more likely.
4. *Probably related*: The time and nature of the AE suggest that it is related to the participation in the study (e.g. arm erythema after exposure to mosquitoes). A different potential etiology is apparent but less likely.
5. *Definitively related*: Those AEs that have a temporal relationship to the intervention under study, which cannot be attributed to another cause.

AE will be classified as expected or unexpected. No serious AEs are expected to occur during the execution of this study.

13.3 AE REPORT

Each AE occurring in the volunteers either related or unrelated to the study procedures, will be registered in a CRF according to GCP standards.

Any SAE or those that threaten the lives of the volunteers and that are classified as possibly or probably related to participation in the study, will be reported electronically or by phone or any other appropriate media to the Ethics Committees and the clinical monitor Ricardo Palacios (phone: 55-11-939-40670) within the first 24 hours after AE occurrence. Detailed information including severity and potential impact on the other participants should be included.

A written report, which will be sent to the aforementioned entities, will be also elaborated. This report shall include the following information:

• Date of report for the AE.

• Voluntary study ID.

• Date of birth, gender and ethnicity of the volunteer.

• Name of the principal investigator.

• Step of the study at which the serious AE occurred.

• Procedures performed to the volunteers during the study and dates on which such procedures were performed.

• Date of onset of the SAE.

• Full description of the SAE.

• Signs or symptoms during the SAE and causality.

• Interventions performed to the volunteers to treat the SAE, including medication doses, route of administration, date of the first and last dose.

• Date of termination of the SAE or death.

• Implications to the volunteer’s health and stay in the study.

• Grade and categorization of the SAE, in relation to the study activities.

• Specific recommendations to ensure the safety of the volunteers, which may result in changes in the protocol.

The written report will be reviewed with the local clinical safety monitor and then sent to the presidents of the Ethics Committees. This will be done within the first 3 business days after the onset of the serious AE. All AE and interventions shall be registered in the folder of each volunteer and included in the reports made to the Ethics Committees.

13.4 PERIOD OF AE MONITORING

All adverse events will be followed until the outcome is classified into one of the following options:

1. Fatal

2. Unsolved

3. Solved

4. Solved with sequels

5. Under resolution

6. Unknown

Pregnancies that occurred during the period spanning from the time of the infectious challenge until day 7 after completing the antimalarial treatment will be followed up by the study research team until completed.

14. ETHICAL CONSIDERATIONS

14.1 APPROVAL BY THE ETHICS COMMITTEES AND ORGANIZATION PLAN

The protocol will be submitted for approval in Colombia to the Human Ethics Committees. This protocol contains the IC forms that must be signed by the participants (Annex A, B and C), which include the conditions on the nature and scientific integrity of the research, and information on the guarantees provided for the volunteers participating in the study. During the course of the study, the PI is responsible for reporting on all events that may affect the safety of individuals and the continuation of the trial. Recruitment activities must not begin until the local Ethics Committees issued the approval.

14.2 AFFILIATION OF THE ETHICS COMMITTEES TO THE UNITED STATES FEDERAL WIDE ASSRANCE (FWA)

The MVDC Ethics Committee named CECIV (for its initials in Spanish: *Comité de Ética del Centro Internacional de Vacunas*) is registered at the Federalwide Assurance (FWA) for the protection of human subjects of the United States Department of Health and Human Services (HHS), Office of Human Research Protection (OHRP), under regulation 45CFR46.103 (CECIV: FWA: FWA00016072). The activities of these institutions with human subjects and all the activities of the Ethics Committee will be conducted in accordance with the provisions of the Declaration of Helsinki (as adopted in 1996 or 2000).

15. RESEARCH RELATED INJURY

Once volunteers are enrolled in Step B of the study, they will be affiliated to the General Social Security System for Health (denoted in Colombia as *Empresa Promotora de Salud*) and to a life insurance policy. Both services will be provided at no cost to the volunteers and throughout the duration of the study. Participants who are injured as result of the participation in the study will receive medical care at no cost to them at a Level III health facility. Volunteers will not receive any other compensation as result of being injured. This situation does not preclude the volunteer from obtaining legal assistance to which he/she is entitled.

16. GOOD CLINICAL PRACTICES (GCPs) AND GOOD LABORATORY PRACTICES (GLPs) IN MVDC AND ASOCLINIC

The MVCD headquarters in Cali will be the site of post-challenge follow-up sessions of the volunteers. MVDC was created in 2000 under the guidance of the World Health Organization (WHO) through the Tropical Disease Research and Training Program (TDR) and currently develops a training program on Good Laboratory Practice (GLP) (M. Arevalo -Herrera PhD). Additionally, the WHO TDR Program has given support for the establishment of GCPs at MVDC. The WHO TDR Program chose Dr. Ricardo Palacios as clinical monitor for the Phase I clinical trials developed by MVDC between 2005 to 2008 to assess the safety and immunogenicity of the *P. vivax* vaccine candidate PvCS. Dr. Ricardo Palacios has keep on working with MVDC and participated as an external monitor of the two previous challenge trials (Herrera *et al.*, 2009; Herrera *et al.*, 2010). The company Meridional R&D founded and directed by him in São Paulo (Brazil) has adapted these monitoring procedures and is enabled to act as Research Organization by Contract (CRO) by the National Council of Scientific and Technological Development (CNPq) of Brazil. During the last 5 years, most group members in Cali have participated in GCP workshops organized and sponsored by NIAID in Brazil and USA, and by other agencies in Colombia. Quality controls related to the study materials will be approved both by the clinical monitor and by the CUIC.

The screening will be done according to standard blood bank screening parameters and in agreement with the standards required by the Ministry of Health. Screening procedures will be carried out in a clinical laboratory duly authorized to perform these activities.

17. CONFIDENTIALITY

All information collected from volunteers will be stored in strict confidentiality. Each person involved in the selection process will be assigned an identification code of 5 digits. Although the names of the participants will be available in the inclusion form, this information will be kept under lock as noted above. All data of the volunteers will be entered into an electronic database in Redcap. The list of names and codes of the volunteers will have a password and only personnel authorized by the Principal Investigator will have access to it. If there are any alterations in the laboratory test results, the volunteers will be contacted as soon as possible to be personally delivered the laboratory reports and received medical counseling*.* Records may be inspected by monitors, auditors and*/*or regulatory authorities. All access to individual records is bounded by strict rules of confidentiality.

18. RULES FOR THE INTERRUPTION OF STUDY.

This study will have duration of 6 months from the time of enrollment of volunteers. The clinical monitor and the PI will review all SAE according to GCP guidelines. The occurrence of AEs possibly related to study procedures will be taken for consideration, by the PI and the clinical monitor, to suspend the study*.* All the reviewing institutions and Ethics Committees will be informed of the progress of these AE by the Principal Investigator and clinical monitor. The CRFs will be reviewed by the clinical monitor and sent to the chairmen of the IRBs in a period no longer than 3 business days. Ethics Committees will review the EAs and decide whether the study can continue or not. MVDC will also review the reports and recommendations of the IRBs, and make the final decision regarding the continuation of the study. The PI will be informed by MVDC on the final decision.

19. USE OF INFORMATION AND PUBLICATION OF STUDY RESULTS

The results of this study are confidential and will be published after approval of the PI and the sponsor institution. It is anticipated that the results of this protocol will be presented to the scientific community through oral presentations at meetings and in written publications in scientific journals. Researchers who are not appointed from the start in this protocol will be required to sign an Underwriting Agreement.

20. DEVIATIONS AND AMENDMENTS TO THE PROTOCOL

Inadvertent breach of this protocol will be considered as a deviation to the protocol and will be reported to each of the monitors as well as to the Ethics Committees. Any changes to the protocol will be informed and put to consideration of the Ethical Committees of each of institution*.*

21. WITHDRAWL OF VOLUNTEERS FROM THE STUDY.

Volunteers participating in any of the two steps (A and B) of the clinical trial may withdraw at any time from the study. A memorandum for record will be written during the course of the study to document withdrawals of volunteers. There will be a specific CRF for volunteer retirement.

21.1 MONITORING OF VOLUNTEER WHO DO NOT CONTINUE IN THE STUDY.

If a volunteer is excluded from the study for any reason after the challenge has taken place but before detection of the disease, the volunteer will be treated according to the protocol immediately and every effort will be made to monitor the volunteer adequately.

If the volunteer presents clinical manifestations secondary to malaria, every effort will be made to provide appropriate treatment and conduct the necessary monitoring evaluations for up to 1 year after the challenge. The reason for the withdrawal of any volunteer shall be registered in the CRF and will be accompanied by a supporting memorandum.

22. FUNDING.

Funding for this study will be obtained through a cooperative agreement between MVDC and the Caucaseco Research Center in Cali, who may also obtain additional funding from other sources if necessary.

23. SCIENTIFIC AGREEMENTS

The signing of this document confirms that each of the undersigned has understood the protocol in its entirety and agree to follow it strictly.

Principal Investigator ____________________

Sócrates Herrera, MD. Date/signature

Co-Investigator ____________________

Myriam Arévalo-Herrera, PhD. Date/signature

Co-Investigator ____________________

Ernesto Martínez Buitrago, MD. Date/signature

Co-Investigator ____________________

Jose Millán Oñate, MD. Date/signature

Independent Clinical monitor ____________________

Ricardo Palacios, MD. Date/signature

# 24. REFERENCES

1. Abdulla S, Oberholzer R, Juma O, Kubhoja S, Machera F, Membi C, Omari S, Urassa A, Mshinda H, Jumanne A, Salim N, Shomari M, Aebi T, Schellenberg DM, Carter T, Villafana T, Demoitie MA, Dubois MC, Leach A, Lievens M, Vekemans J, Cohen J, Ballou WR, Tanner M, 2008. Safety and immunogenicity of RTS,S/AS02D malaria vaccine in infants. *N Engl J Med 359:* 2533—2544
2. Arévalo-Herrera M, Roggero MA, Gonzalez JM, Vergara J, Corradin G, López JA, Herrera, S. Mapping and comparison of the B-cell epitopes recognized on the *Plasmodium vivax* circumsporozoite protein by immune Colombians and immunized *Aotus* monkeys. Ann. Trop. Med. Parasitol.1998. 92(5):539-51.
3. Arévalo-Herrera, M. and Herrera, S. *Plasmodium vivax* malaria vaccine development. Mol Immunol. 2001. 38(6):443-55.
4. Arévalo-Herrera, M., Castellanos, A., Yazdani, S., Rushdi, A., Chitnis, C., Dominik, R., Herrera, S. Immunogenicity and protective efficacy of recombinant vaccine base on the receptor-binding domain of the *Plasmodium vivax* duffy binding protein in *Aotus* monkeys. Am J Trop Med Hyg. 2005a. 73(5):25-31.
5. [Arévalo-Herrera](http://www.ncbi.nlm.nih.gov/entrez/query.fcgi?db=pubmed&amp;cmd=Search&amp;itool=pubmed_Abstract&amp;term=%22Arevalo%2DHerrera%2BM%22%5BAuthor%5D) M, [Solarte Y](http://www.ncbi.nlm.nih.gov/entrez/query.fcgi?db=pubmed&amp;cmd=Search&amp;itool=pubmed_Abstract&amp;term=%22Solarte%2BY%22%5BAuthor%5D), [Zamora F](http://www.ncbi.nlm.nih.gov/entrez/query.fcgi?db=pubmed&amp;cmd=Search&amp;itool=pubmed_Abstract&amp;term=%22Zamora%2BF%22%5BAuthor%5D), [Mendez F](http://www.ncbi.nlm.nih.gov/entrez/query.fcgi?db=pubmed&amp;cmd=Search&amp;itool=pubmed_Abstract&amp;term=%22Mendez%2BF%22%5BAuthor%5D), [Yasnot MF](http://www.ncbi.nlm.nih.gov/entrez/query.fcgi?db=pubmed&amp;cmd=Search&amp;itool=pubmed_Abstract&amp;term=%22Yasnot%2BMF%22%5BAuthor%5D), [Rocha L](http://www.ncbi.nlm.nih.gov/entrez/query.fcgi?db=pubmed&amp;cmd=Search&amp;itool=pubmed_Abstract&amp;term=%22Rocha%2BL%22%5BAuthor%5D), [Long](http://www.ncbi.nlm.nih.gov/entrez/query.fcgi?db=pubmed&amp;cmd=Search&amp;itool=pubmed_Abstract&amp;term=%22Long%2BC%22%5BAuthor%5D) C, [Miller LH](http://www.ncbi.nlm.nih.gov/entrez/query.fcgi?db=pubmed&amp;cmd=Search&amp;itool=pubmed_Abstract&amp;term=%22Miller%2BLH%22%5BAuthor%5D), [Herrera S](http://www.ncbi.nlm.nih.gov/entrez/query.fcgi?db=pubmed&amp;cmd=Search&amp;itool=pubmed_Abstract&amp;term=%22Herrera%2BS%22%5BAuthor%5D). *Plasmodium vivax*: transmission-blocking immunity in a malaria-endemic area of Colombia. Am J Trop Med Hyg. 2005b. 73(5):38-43.
6. [Arévalo-Herrera](http://www.ncbi.nlm.nih.gov/entrez/query.fcgi?db=pubmed&amp;cmd=Search&amp;itool=pubmed_Abstract&amp;term=%22Arevalo%2DHerrera%2BM%22%5BAuthor%5D) M, [Solarte Y](http://www.ncbi.nlm.nih.gov/entrez/query.fcgi?db=pubmed&amp;cmd=Search&amp;itool=pubmed_Abstract&amp;term=%22Solarte%2BY%22%5BAuthor%5D), [Yasnot MF](http://www.ncbi.nlm.nih.gov/entrez/query.fcgi?db=pubmed&amp;cmd=Search&amp;itool=pubmed_Abstract&amp;term=%22Yasnot%2BMF%22%5BAuthor%5D), [Castellanos A](http://www.ncbi.nlm.nih.gov/entrez/query.fcgi?db=pubmed&amp;cmd=Search&amp;itool=pubmed_Abstract&amp;term=%22Castellanos%2BA%22%5BAuthor%5D), [Rincon A](http://www.ncbi.nlm.nih.gov/entrez/query.fcgi?db=pubmed&amp;cmd=Search&amp;itool=pubmed_Abstract&amp;term=%22Rincon%2BA%22%5BAuthor%5D), [Saul A](http://www.ncbi.nlm.nih.gov/entrez/query.fcgi?db=pubmed&amp;cmd=Search&amp;itool=pubmed_Abstract&amp;term=%22Saul%2BA%22%5BAuthor%5D), [Mu](http://www.ncbi.nlm.nih.gov/entrez/query.fcgi?db=pubmed&amp;cmd=Search&amp;itool=pubmed_Abstract&amp;term=%22Mu%2BJ%22%5BAuthor%5D) J, [Long C](http://www.ncbi.nlm.nih.gov/entrez/query.fcgi?db=pubmed&amp;cmd=Search&amp;itool=pubmed_Abstract&amp;term=%22Long%2BC%22%5BAuthor%5D), [Miller L](http://www.ncbi.nlm.nih.gov/entrez/query.fcgi?db=pubmed&amp;cmd=Search&amp;itool=pubmed_Abstract&amp;term=%22Miller%2BL%22%5BAuthor%5D), [Herrera S](http://www.ncbi.nlm.nih.gov/entrez/query.fcgi?db=pubmed&amp;cmd=Search&amp;itool=pubmed_Abstract&amp;term=%22Herrera%2BS%22%5BAuthor%5D). Induction of transmission-blocking immunity in *Aotus* monkeys by vaccination with a *Plasmodium vivax* clinical grade PvS25 recombinant protein. Am J Trop Med Hyg. 2005c;73(5):32-37.
7. Arévalo-Herrera, M., Chitnis, C., Herrera, S., 2010, Current status of *Plasmodium vivax* vaccine. 2010. Human Vaccines 6:1, 1-9. Ed. Landes Bioscience.
8. Baird, JK., Tiwari, T., Martin, GJ., Tamminga, CL., Prout, TM., Tjaden, J., Bravet, P., Rawlins, S., Ferrel, M., Carucci, D., Hoffman, SL. Chloroquine for the treatment of uncomplicated malaria in Guyana. Ann Trop Med Parasitol. 2002. 96(4):339-48.9.
9. Ballou, WR., Arévalo-Herrera, M., Carucci, D., Richie, TL., Corradin, G., Diggs, C., Druilhe, P., Giersing, BK., Saul, A., Heppner DG., Kester, KE., Lanar, DE., Lyon, J., Hill, AV. Pan, W., Cohen, JD. Update on the clinical development of candidate malaria vaccines. Am J Trop Med Hyg. 2004 71(2):239-47.
10. Baton, LA., Ranford-Cartwright, L.C. How do malaria ookinetes cross the mosquito midgut wall? Trends Parasitol. 2005 1(1):22-8.
11. Blair, S., Lopez, ML., Pineros, JG., Alvarez, T., Tobon, A., Carmona J. Therapeutic efficacy of treatment protocols for non-complicated *Plasmodium falciparum* malaria, Antioquia, Colombia, 2002. Biomedica. 2003. 23:318-27.
12. Blow, JA., Turell, MJ., Walker, ED.,Silverman, AL. Post-blood meal diuretic shedding of hepatitis B virus by mosquitoes (Diptera: Culicidae). J Med Entomol. 2002. 39(4):605-12.
13. Bosman, A., Delacollette, C., Olumese, P., Ridley, RG., Rietveld, R., Shretta, R., Teklehaimanot, A. The use of antimalarial drugs. Report of an Informal Consultation. Roll Back Malaria. WHO. 2001.
14. Chaparro PE. *Comportamiento de la malaria en Colombia según los casos notificados al SIVIGILA en 2009. INS*. 2009. Available at: [http://www.ins.gov.co/?idcategoria=49752#](http://www.ins.gov.co/?idcategoria=49752)
15. Chauhan, VS., and Bhardwaj, D. Current status of malaria vaccine development. Adv Biochem Eng Biotechnol 2003.84:143-82.
16. Chappel, JA., Rogers, WO., Hoffman, SL., Kang, AS. Molecular dissection of the human antibody response to the structural repeat epitope of *Plasmodium* *falciparum* sporozoite from a protected donor. Malar J. 2004. 29:3-28.
17. Chotivanich, K., Silamut, K., UdWHOangpetch, R., Stepniewska, KA., Pukrittayakamee, S., Looareesuwan, S., White, NJ. Ex-vivo short-term culture and developmental assessment of *Plasmodium vivax.* Trans R Soc Trop Med Hyg. 2001. 95(6):677-80.
18. Chulay, JD., Schneider, I., Cosgriff, TM., Hoffman, SL., Ballou, WR., Quakyi, IA., Carter, R., Trosper, JH., Hockmeyer, WT. Malaria transmitted to humans by mosquitoes infected from cultured *Plasmodium falciparum*. Am J Trop Med Hyg 1986. 35(1):66-68.
19. Church, LW., Le, TP., Bryan, JP., Gordon, DM., Edelman, R., Fries, L., Davis, JR., Herrington, DA., Clyde, DF., Shmuklarsky, MJ., Schneider, I., McGovern, TW., Chulay, JD., Ballou, WR., Hoffman, SL. Clinical manifestations of *Plasmodium falciparum* malaria experimentally induced by mosquito challenge. J Infect Dis.1997. 175(4):915-20.
20. Clyde, DF., Most, H., McCarthy, VC.,Vanderberg, JP. Immunization of man against sporozoite-induced falciparum malaria. Am J Med Sci 1973. 266:169-77.
21. Clyde, DF. Immunization of man against *falciparum* and *vivax* malaria by use of attenuated sporozoites. Am J Trop Med Hyg. 1975. 24(3):397-01.
22. Clyde, DF. Immunity to *falciparum* and *vivax* malaria induced by irradiated sporozoites: a review of the University of Maryland studies, 1971-75. Bull World Health Organ. 1990 (68):9-12.
23. Cogswell FB. The hypnozoite and relapse in primate malaria. Clin Microbiol Rev. 1992. 5(1):26-35.
24. Collins, WE, and Jeffery, GM. Primaquine resistance in *Plasmodium vivax*. Am J Trop Med Hyg 1996. 55(3):243-49.
25. Danis, M. Therapeutic advances against malaria in 2003. Med Trop (Mars) 2003. 63:267- 70.
26. Doolan, DL., Hoffman, SL. Nucleic acid vaccines against malaria. Chem Immunol. 2002. 80:308-21.
27. Duarte, EC., Pang, LW., Ribeiro, LC, Fontes, CJ. Association of subtherapeutic dosages of a standard drug regimen with failures in preventing relapses of vivax malaria. Am J Trop Med Hyg. 2001. 65(5):471-76.
28. Edelman, R. Long-term persistence of sterile immunity in a volunteer immunized with X-irradiated *Plasmodium falciparum* sporozoites*.* J Infect Dis, 1993. 168:1066-1070.
29. Egan JE, Hoffman SL, Haynes JD, Sadoff JC, Schneider I, Grau GE, Hollingdale MR, Ballou WR, Gordon DM. Humoral immune responses in volunteers immunized with irradiated *Plasmodium falciparum* sporozoites. Am J Trop Med Hyg, 1993. 49(2):166-73.
30. Fairley, NH. Sidelights on malaria in man obtained by subinoculation experiments. Trans Royal Soc Trop Med Hyg. 1947. 40: 621-76.
31. Fernández, O., Manzano, M.R, Murrain, B., Blanco P., Zamora F., Jordan, A. Palacios, R., Velez, D., Arévalo-Herrera, M., Herrera, S. Development of a sporozoite challenge model for *Plasmodium vivax* in human volunteers. ASTMH. Annual Meeting 54th. 2005.
32. Fryauff, DJ., Baird, JK., Basri, H., Sumawinata, IW., Purnomo., Richie, TL., Ohrt, C., Mouzin, E., Church, CJ., Richards, AL., Subianto, B., Sandjaja, B., Wignall, FS., Hoffman, SL. Randomized placebo-controlled trial of primaquine for causal prophylaxis against vivax and falciparum malaria. Lancet. 1995. 346(8984):1190-93.
33. Genton, B, and Corradin, G. Malaria vaccines: from the laboratory to the field. Curr Drug Targets Immune Endocr Metabol Disord. 2002(3):255-67.
34. George F W, 4th, Law, JL., Rich, KA., Martin, WJ. Identification of a T cell epitope on the circumsporozoite protein of *Plasmodium vivax*. Infect. Immun. 1990,58(2):575-78.
35. Glynn, JR. Infecting dose and severity of malaria: a literature review of induced malaria. J Trop Med Hyg. 1994. 97(5):300-16.
36. Glynn, JR. and Bradley, DJ. Inoculum size, incubation period and severity of malaria. Analysis of data from malaria therapy records. Parasitology 1995.110:7-19.
37. Glynn, JR., Collins, WE., Jeffery, GM., Bradley, DJ. Infecting dose and severity of falciparum malaria. Trans R Soc Trop Med Hyg 1995. 89:281-83.
38. Golenda, CF., Li, J., and Rosenberg, R. Continuous in vitro propagation of the malaria parasite *Plasmodium vivax.* Proc Natl Acad Sci U. S. A. 1997.94:6786-91.
39. Good, MF., Pombo, D., Quakyi, IA., Riley, EM., Houghten, RA., Menon, A., Alling, DW., Berzofsky, JA., Miller, LH. Human T-cell recognition of the circumsporozoite protein of *Plasmodium falciparum*: immunodominant T cell domains map to the polymorphic regions of the molecule. Proc. Natl. Acad. Sci. U. S. A. 1988. 85(4):1199-203.
40. Grassi, B., Bignami, A., Bastianelli, G. Ulteriori ricerche sul ciclo dei parassiti malarici umani nel corpo del zanzarone. Atti Reale Accademia dei Lincei. 1899.5:8-21.
41. Haddy, TB., Rana, SR., Castro O. Benign ethnic neutropenia: What is a normal absolute neutrophil count?. J Lab Clin Med. 1999, 133(1):15-22
42. Hermsen, CC., De Vlas, SJ., Van Gemert, GJ., Telgt, DS., Verhage, DF., Sauerwein, RW. Testing vaccines in human experimental malaria: statistical analysis of parasitemia measured by a quantitative real-time polymerase chain reaction. Am J Trop Med Hyg. 2004.71(2):196-201.
43. Herrera S., Fernandez, O.L., Vera O., Cardenas, W., Ramirez O., Palacios, R., Chen-Mok, M., Corradin, G., Arévalo-Herrera M. Phase I Safety and Immunogenicity Trial of *Plasmodium vivax* CS Derived Long Synthetic Peptides Adjuvanted with Montanide ISA 720 or Montanide ISA 51. Am. J. Trop. Med. Hyg., 84(Suppl 2), 2011, pp. 12–20.
44. [Herrera S](http://www.ncbi.nlm.nih.gov/entrez/query.fcgi?db=pubmed&amp;cmd=Search&amp;itool=pubmed_Abstract&amp;term=%22Herrera%2BS%22%5BAuthor%5D), [Bonelo](http://www.ncbi.nlm.nih.gov/entrez/query.fcgi?db=pubmed&amp;cmd=Search&amp;itool=pubmed_Abstract&amp;term=%22Bonelo%2BA%22%5BAuthor%5D) A, [Perlaza BL](http://www.ncbi.nlm.nih.gov/entrez/query.fcgi?db=pubmed&amp;cmd=Search&amp;itool=pubmed_Abstract&amp;term=%22Perlaza%2BBL%22%5BAuthor%5D), [Fernandez OL](http://www.ncbi.nlm.nih.gov/entrez/query.fcgi?db=pubmed&amp;cmd=Search&amp;itool=pubmed_Abstract&amp;term=%22Fernandez%2BOL%22%5BAuthor%5D), [Victoria L](http://www.ncbi.nlm.nih.gov/entrez/query.fcgi?db=pubmed&amp;cmd=Search&amp;itool=pubmed_Abstract&amp;term=%22Victoria%2BL%22%5BAuthor%5D), [Lenis](http://www.ncbi.nlm.nih.gov/entrez/query.fcgi?db=pubmed&amp;cmd=Search&amp;itool=pubmed_Abstract&amp;term=%22Lenis%2BAM%22%5BAuthor%5D) AM, [Soto L](http://www.ncbi.nlm.nih.gov/entrez/query.fcgi?db=pubmed&amp;cmd=Search&amp;itool=pubmed_Abstract&amp;term=%22Soto%2BL%22%5BAuthor%5D), [Hurtado H](http://www.ncbi.nlm.nih.gov/entrez/query.fcgi?db=pubmed&amp;cmd=Search&amp;itool=pubmed_Abstract&amp;term=%22Hurtado%2BH%22%5BAuthor%5D), [Acuna L](http://www.ncbi.nlm.nih.gov/entrez/query.fcgi?db=pubmed&amp;cmd=Search&amp;itool=pubmed_Abstract&amp;term=%22Acuna%2BLM%22%5BAuthor%5D)M, [Velez](http://www.ncbi.nlm.nih.gov/entrez/query.fcgi?db=pubmed&amp;cmd=Search&amp;itool=pubmed_Abstract&amp;term=%22Velez%2BJD%22%5BAuthor%5D) JD, [Palacios](http://www.ncbi.nlm.nih.gov/entrez/query.fcgi?db=pubmed&amp;cmd=Search&amp;itool=pubmed_Abstract&amp;term=%22Palacios%2BR%22%5BAuthor%5D) R, [Chen-Mok](http://www.ncbi.nlm.nih.gov/entrez/query.fcgi?db=pubmed&amp;cmd=Search&amp;itool=pubmed_Abstract&amp;term=%22Chen%2DMok%2BM%22%5BAuthor%5D) M, [Corradin G](http://www.ncbi.nlm.nih.gov/entrez/query.fcgi?db=pubmed&amp;cmd=Search&amp;itool=pubmed_Abstract&amp;term=%22Corradin%2BG%22%5BAuthor%5D), [Arévalo-Herrera](http://www.ncbi.nlm.nih.gov/entrez/query.fcgi?db=pubmed&amp;cmd=Search&amp;itool=pubmed_Abstract&amp;term=%22Arevalo%2DHerrera%2BM%22%5BAuthor%5D) M. Safety and elicitation of humoral and cellular responses in colombian malaria-naive volunteers by a *Plasmodium vivax* circumsporozoite protein-derived synthetic vaccine. Am J Trop Med Hyg. 2005. 73(5):3-9.
45. Herrera, MA., De Plata, C., Gonzalez, JM., Corradin, G.,Herrera, S. Immunogenicity of multiple antigen peptides containing *Plasmodium vivax* CS epitopes in BALB/c mice. Mem Inst Oswaldo Cruz 1994. 89:71-76.
46. Herrera, S., De Plata, C., Gonzalez, JM., Perlaza, BL., Bettens, F., Corradin, G., Arévalo-Herrera, M. Antigenicity and immunogenicity of multiple antigen peptides (MAP) containing *P. vivax* CS epitopes in *Aotus* monkeys. Parasite Immunol 1997.19:161-170.
47. Herrera, S., Escobar, P., De Plata, C., Avila, GI., Corradin, G., Herrera MA. Human recognition of T cell epitopes on the *Plasmodium vivax* circumsporozoite protein. J. Immunol. 1992. 148:3986-3990.
48. Herrera, S., Manzano, M., Fernandez, O., Solarte, Y., Rocha, L., Vergara, J., Bermans, M., Acuña, L., Londoño, C., Palacios, R., Rincon, A., Yansot, MF., Arévalo-Herrera, M. Successful sporozoite challenge model in human volunteers with *Plasmodium* vivax strain derived from human donors. Am J Trop Med Hyg. 2009;81(5):740-6.
49. [Herrera, S](http://www.ncbi.nlm.nih.gov/entrez/query.fcgi?db=pubmed&amp;cmd=Search&amp;itool=pubmed_Abstract&amp;term=%22Herrera%2BS%22%5BAuthor%5D)., [Perlaza, BL](http://www.ncbi.nlm.nih.gov/entrez/query.fcgi?db=pubmed&amp;cmd=Search&amp;itool=pubmed_Abstract&amp;term=%22Perlaza%2BBL%22%5BAuthor%5D)., [Bonelo,](http://www.ncbi.nlm.nih.gov/entrez/query.fcgi?db=pubmed&amp;cmd=Search&amp;itool=pubmed_Abstract&amp;term=%22Bonelo%2BA%22%5BAuthor%5D) A., [Arévalo-Herrera,](http://www.ncbi.nlm.nih.gov/entrez/query.fcgi?db=pubmed&amp;cmd=Search&amp;itool=pubmed_Abstract&amp;term=%22Arevalo%2DHerrera%2BM%22%5BAuthor%5D) M. *Aotus* monkeys: their great value for anti-malaria vaccines and drug testing. Int J Parasitol. 2002. 32(13):1625-35.
50. Herrera, S and Arévalo-Herrera M., Progress Toward the Development of a *Plasmodium Vivax* Malaria Vaccine, 2010a. *Immune Response to Parasitic Infections,* 43-54. Bentham Science Publishers Ltd.
51. Herrera S, Solarte Y, Jordán-Villegas A, Echavarría JF, Rocha L, Palacios R, Ramírez O, Vélez JD, Epstein JE, Richie TL, Arévalo-Herrera M. Consistent Safety and Infectivity in Sporozoite Challenge Model of *Plasmodium vivax* in Malaria-Naïve Human Volunteers. Am. J. Trop. Med. Hyg., 2011; 84(Suppl 2): 4-11.
52. Herrington D, Davis J, Nardin E, Beier M, Cortese J, Eddy H, Losonsky G, Hollingdale M, Sztein M, Levine M. Successful immunization of humans with irradiated malaria sporozoites: humoral and cellular responses of the protected individuals*.* Am J Trop Med Hyg. 1991. 45(5):539-47.
53. Hisaeda, H. and Yasutomo, K. Development of malaria vaccines that block transmission of parasites by mosquito vectors. J Med Invest 2002. 49(3-4):118-23
54. Hoffman, SL. Experimental Challenge of Volunteers with Malaria. Ann Intern Med. 1997:127(3): 233-35.
55. Hoffman, SL., Goh, LM., Luke, TC., Schneider, I., Le, TP., Doolan, DL., Sacci, J., de la Vega, P., Dowler, M., Paul, C., Gordon, DM., Stoute, JA., Church, LW., Sedegah, M., Heppner, DG., Ballou, WR., Richie, TL. Protection of humans against malaria by immunization with radiation- attenuated *Plasmodium falciparum* sporozoites. J Infect Dis. 2002:185(8):1155-64.
56. Hurtado, S., Salas, ML., Romero, JF., Zapata, JC., Ortiz, H., Arévalo- Herrera, M., Herrera, S. Regular production of infective sporozoites of *Plasmodium falciparum* and *P. vivax* in laboratory-bred *Anopheles albimanus.* Ann. Trop. Med. Parasitol. 1997. 91:49-60.
57. Jordan, A., Bonelo A., Epstein, JE., López, J., Castellanos, A,. Manzano, MR., Hernández, MA., Soto, L., Méndez, F., Richie, TL., Hoffman., SL., Arévalo-Herrera, M. and Herrera, S. Cellular and humoral response of *P vivax* irradiated sporozoites in *Aotus* monkeys Am. J. Trop. Med. Hyg., 84(Suppl 2), 2011, pp. 43–50.
58. Krotoski, WA. The hypnozoite and malarial relapse. Prog Clin Parasitol. 1989. 1:1-19.
59. Luke, TC. and Hoffman, SL. Rationale and plans for developing a non- replicating, metabolically active, radiation-attenuated *Plasmodium falciparum* sporozoite vaccine. J Exp Biol. 2003.206:3803-08
60. Herrera S, Fernández O, Manzano MR, Murrain B, Vergara J, Blanco P, Palacios R, Vélez JD, Epstein JE, Chen-Mok M, Reed ZH, Arévalo-Herrera M. Successful sporozoite challenge model in human volunteers with *Plasmodium vivax* strain derived from human donors. Am J Trop Med Hyg. 2009;81(5):740-6.
61. McCarthy,VC., and Clyde, DF. *Plasmodium vivax*: correlation of circumsporozoite precipitation (CSP) reaction with sporozoite-induced protective immunity in man. Exp Parasitol. 1977. 41(1):167-71.
62. McGready, R., Thwai, KL., Cho, T., Samuel., Looareesuwan, S., White, NJ., Nosten, F. The effects of quinine and chloroquine antimalarial treatments in the first trimester of pregnancy. Trans R Soc Trop Med Hyg. 2002. 96(2):180-84.
63. McGregor, IA., Gilles, HM., Walters, JH., Davies, AH., Pearson, FA. Effects of heavy and repeated malarial infections on Gambian infants and children; effects of erythrocytic parasitation. Br Med J 1956. 32(4994): 686-92.
64. Meis, JF., Ponnudurai, T., Mons, B., Van Belkum, A., Van Eerd, PM., Druilhe, P. Schellekens, H. *Plasmodium falciparum*: studies on mature exoerythrocytic forms in the liver of the chimpanzee, *Pan troglodytes*. Exp Parasitol. 1990(70):1-11.
65. Mendis, K., Sina, BJ., Marchesini, P.,Carter, R. The neglected burden of *Plasmodium vivax* malaria. Am J Trop Med Hyg. 2001. 64(1-2):97-106.
66. Ministerio de Salud, Republica de Colombia. 2000. Guía de atención de la malaria. Available at: <http://www.metrosalud.gov.co/Paginas/Protocolos/MinSalud/guias/31-MALARIA.htm>.
67. Moorthy, V.S., Diggs, C., Ferro,S., Good, M.F., Herrera, S., Hill, A.V., Imoukhuedeh, E.B., S. Kumari, Loucq, C., Marsh, K. Ockenhouse, C.F.,. Richie T.L., Sauerwein R.W. Report of a Consultation on the Optimization of Clinical Challenge Trials for Evaluation of Candidate Blood Stage Malaria Vaccines,18–19 March 2009, Bethesda, MD, USA. (2009). Vaccine 27 5719–5725.
68. Nardin, E., Clavijo, P., Mons, B., van Belkum, A., Ponnudurai, T., Nussenzweig, RS. T cell epitopes of the circumsporozoite protein of *Plasmodium vivax*. Recognition by lymphocytes of a sporozoite-immunize chimpanzee. J. Immunol. 1991. 146(5): 1674-78
69. NCI., NIH., DCTD., DHHS. Common Terminology Criteria for Adverse Events v3.0. 2003. <http://ctep.cancer.gov/forms/CTCAEv3.pdf>
70. Nosten, F., McGready, R., Simpson, JA., Thwai, KL., Balkan, S., Cho, T., Hkirijaroen, L., Looareesuwan, S., White, NJ. Effects of *Plasmodium vivax* malaria in pregnancy. Lancet. 1999. 354(9178):546-49.
71. WHO. 2005. World Malaria Report 2005. Prepared by Roll Back Malaria, WHO and Unice[f. Available at: http://www.rbm.who.int/wmr2005/pdf/WMReport_lr.pdf](http://www.rbm.who.int/wmr2005/pdf/WMReport_lr.pdf).
72. WHO. 2009. World Malaria Report 2009. [Available at: http://whqlibdoc.who.int/publications/2009/9789241563901_eng.pdf](http://whqlibdoc.who.int/publications/2009/9789241563901_eng.pdf)
73. Padilla, JC., Guhl, F., Soto, J., Alvarez, G. *Diagnóstico and terapéutica de las enfermedades transmitidas por vectores en Colombia*.1999, v.2500. p.128
74. Perlaza, BL., Zapata, C., Valencia, AZ., Hurtado, S., Quintero, G., Sauzet, JP., Brahimi, K., Blanc, C., Arévalo-Herrera, M., Druilhe, P.,Herrera, S. Immunogenicity and protective efficacy of *Plasmodium falciparum* liver-stage Ag-3 in *Aotus lemurinus griseimembra* monkeys. Eur J Immunol. 2003. 33:1321-27
75. Phillips-Howard PA. Epidemiological and control issues related to malaria in pregnancy. Ann Trop Med Parasitol. 1999.93(1):S11-17
76. Powell, RD. and McNamara, JV. Infection with chloroquine-resistant *Plasmodium falciparum* in man: prepatent periods, incubation periods, and relationships between parasitemia and the onset of fever in nonimmune persons. Ann N and Acad Sci. 1970. 174:1027-41.
77. Rieckmann, KH. Human immunization with attenuated sporozoites. Bull World Health Organ. 1990. 68Suppl:13-16.
78. Rieckmann, KH., Beaudoin RL., Cassells JS, Sell KW. Use of attenuated sporozoites in the immunization of human volunteers against falciparum malaria*.* Bull World Health Organ. 1979. 57 (1):261-65.
79. Roestenberg, M., McCall, M., Hopman, J., Wiersma, J,. Luty, A., van Gemert G.J,, van de Vegte-Bolmer, M., van Schaijk, B., Teelen, K., Arens, T., Spaarman, L., de Mast, Q., Roeffen,W., Snounou, G., Renia, L., van der Ven, A., Hermsen, C., Sauerwein, R. Protection against a Malaria Challenge by Sporozoite Inoculation, 2009. N Engl J Med;361:468-77.
80. Salas M.L., Romero J., Solarte Y., Olano V., Herrera, M., Herrera, S., Development of sporogonic cycle of *Plasmodium vivax* in experimentally infected *Anopheles albimanus* mosquitoes. Mem. Inst. Oswaldo Cruz. 1994:115-19
81. Singh, N., Shukla, MM., Sharma, VP. Epidemiology of malaria in pregnancy in central India. Bull World Health Organ. 1999; 77(7):567-72.
82. SIVIGILA. *Boletin vigilancia de la malaria en Colombia. Boletín: octubre 29 de 2010*. Elaborated by Grupo ETV (INS). Available at: [http://new.paho.org/col/index.php?option=com_docman&task=doc_download&gid](http://new.paho.org/col/index.php?option=com_docman&amp;task=doc_download&amp;gid)=663&Itemid=
83. Snounou G, Viriyakosol S, Jarra W, Thaithong S, Brown KN. Identification of the four human malaria parasite species in field samples by the polymerase chain reaction and detection of a high prevalence of mixed infections. Mol Biochem Parasitol. 1993. 58(2):283-92.
84. Soto, J., Toledo, J., Gutiérrez, P., Luz, M., Llinas, N., Cedeno, N., Dunne, M.,Berman, J. *Plasmodium vivax* clinically resistant to chloroquine in Colombia. Am J Trop Med Hyg 2001.65 (2):90-93.
85. Stoute JA, Slaoui M, Heppner DG, Momin P, Kester KE, Desmons P, Wellde BT, Garcon N, Krzych U, Marchand M, 1997. A preliminary evaluation of a recombinant circumsporozoite protein vaccine against *Plasmodium falciparum* malaria. RTS,S Malaria Vaccine Evaluation Group. *N Engl J Med 336:* 86--91.
86. Talisuna, A., Bloland, P. D’Alessandro, U. History, dynamics, and publichealth importance of malaria parasite resistance. Clin Microbiol Rev. 2004; 17(1):235–54
87. Trigg, PI., and Kondrachine, AV. 1998. The current global malaria situation, p. 11-22. *In* I. Sherman (ed), Malaria: Parasite biology, pathogenesis and protection. AMS Press, Washington DC.
88. UNDP/World Bank/WHO Special Programme for Research and Training in Tropical Diseases (TDR). Guidelines for the evaluation of *Plasmodium falciparum* vaccines in populations exposed to natural infection. Available at: [http://apps.who.int/tdr/svc/publications/training-guideline- publications/evaluation-of-*Plasmodium*-falciparum](http://apps.who.int/tdr/svc/publications/training-guideline-publications/evaluation-of-plasmodium-falciparum)
89. Valderrama, A., Quintero, G., Gómez, A., Castellanos, A., Pérez, Y., Méndez, F., Arévalo-Herrera, M., Herrera, S. Antigenicity, immunogenecity and protective efficacy of *Plasmodium vivax* MSP1 PV200L: A potential malaria vaccine subunit. Am J Trop Med Hyg. 2005. 73(5):16-24.
90. WHO/PAHO, Buenas Prácticas Clínicas: Documento de las Américas. 2005, p101.
91. Wilairatana, P., Krudsood, S., Treeprasertsuk, S., Chalermrut, K., and Looareesuwan, S. The future outlook of antimalarial drugs and recent work on the treatment of malaria. Arch Med Res 2002. 33:(4)416-21.
92. Walther, M., Dunachie, S., Keating, S., Vuola, JM., Berthoud, T., Schmidt, A., Maier, C., Andrews, L., Andersen, RF., Gilbert, S. *et al*. Safety, immunogenicity and efficacy of a pre-erythrocytic malaria candidate vaccine, ICC-1132 formulated in Seppic ISA 720. Vaccine. 2005. 23(7):857-64.
93. Yagmur, Y., Kara, IH., Aldemir, M., Büyükbayram, H., Tacyildiz, IH., Keles, C. Spontaneous rupture of malarial spleen: two case reports and review of literature. Crit Care 2000. 4(5):309-13
94. Zapata, J., Perlaza, BL., Hurtado, S., Quintero, GE., Jurado, D., Gonzáles, I., Druilhe, P., Arévalo- Herrera, M., Herrera, S. Reproducible infection of intact *Aotus Lemorinus Griseimembra* monkeys by *Plasmodium falciparum* sporozoite inoculation*.* J Parasitol 2002. 88(4): 723-9.

# ANNEX 1: STUDY PROCEDURES

## Step A Procedures

| Step A | | |
| --- | --- | --- |
| Procedure | Visit 1 (Day 0) | Visit 2 (Day 12 ± 2) |
| Recruitment | x |  |
| Informed Consent | x |  |
| Medical Examination | x |  |
| Treatment Administration | x |  |
| Infectious Diseases Screening | x |  |
| Appointment for Medical Control AppointmentCitacontrol medico |  | x |
| Delivery of Screening Results |  | x |

## Step B Procedures

| Step B | | | | | | | | | |
| --- | --- | --- | --- | --- | --- | --- | --- | --- | --- |
| Procedure | S1 Visit  (C0Day -30) | S2 Visit  (C0Day -15) | S3 Visit  (C0Day -1) | Challenge Visit (C0Day) | C1-28 Visit  (C7-C28 Day*) | T1-14 Visit  (TTO-TT Day 14**) | T15 Visit  (TT Day 15) | T16 Visit  (TT +3wks) | T17 Visit  (TT +6months) |
| Recruitment | X |  |  |  |  |  |  |  |  |
| Informed Consent | X |  |  |  |  |  |  |  |  |
| Medical Examination | X |  |  |  |  |  |  |  |  |
| Pregnancy test (women) | X |  |  |  |  |  | X |  |  |
| Urinalysis | X |  |  |  |  |  |  |  |  |
| Duffy | X |  |  |  |  |  |  |  |  |
| Infectious diseases screening | X |  |  |  |  |  |  |  |  |
| Hemogram | X |  |  |  |  |  |  | X | X |
| Blood Chemistry | X |  |  |  |  |  |  | X | X |
| G6PD deficiency | X |  |  |  |  |  |  |  |  |
| Delivery of results |  | X |  |  |  |  |  |  |  |
| Electrocardiogram |  | X |  |  |  |  |  |  |  |
| Volunteer selection |  | X |  |  |  |  |  |  |  |
| EPS Affiliation/Medical Insurance |  | X |  |  |  |  |  |  |  |
| Medical control appointment/Physical Exam |  |  | X |  |  |  |  |  |  |
| Blood sampling for immunological studies |  |  | X |  |  | X |  | X | X |
| Pregnancy test (women) |  |  | X |  |  |  |  |  |  |
| Infectious Challenge |  |  |  | X |  |  |  |  |  |
| Individual follow up |  |  |  |  | X | X | X |  |  |
| TBS |  |  |  |  | X | X |  |  |  |
| Filter paper |  |  |  |  | X | X |  |  |  |
| Medical examination |  |  |  |  | X | X |  |  |  |
| Treatment |  |  |  |  |  | X |  |  |  |
